# Exploring the Potential of Coumarin Derivatives on Serotonin Receptors 5-HT_1A_ and 5HT_2A_

**DOI:** 10.3390/ijms26051946

**Published:** 2025-02-24

**Authors:** Kinga Ostrowska, Gabriela Horosz, Karolina Kruk, Bartłomiej Sieroń, Anna Leśniak, Zofia Czartoryska, Magdalena Bujalska-Zadrożny, Dejan Milenkovic, Bartosz Trzaskowski

**Affiliations:** 1 Department of Organic and Physical Chemistry, Faculty of Pharmacy, Medical University of Warsaw, Banacha 1, 02-097 Warsaw, Poland; gabriela.horoszz@gmail.com (G.H.); s080615@student.wum.edu.pl (K.K.); s080954@student.wum.edu.pl (B.S.); 2 Faculty of Pharmacy, Department of Pharmacotherapy and Pharmaceutical Care, Medical University of Warsaw, Banacha 1, 02-097 Warsaw, Poland; anna.lesniak@wum.edu.pl (A.L.); s080853@student.wum.edu.pl (Z.C.); magdalena.bujalska@wum.edu.pl (M.B.-Z.); 3 Department of Science, Institute for Information Technologies, University of Kragujevac, Jovana Cvijića bb, 34000 Kragujevac, Serbia; dejanm@uni.kg.ac.rs; 4 Centre of New Technologies, University of Warsaw, 2C Banacha Str., 02-097 Warsaw, Poland; b.trzaskowski@cent.uw.edu.pl

**Keywords:** coumarin derivatives, 5HT_1A_, 5HT_2A_ receptors ligands, microwave-assisted synthesis, molecular docking

## Abstract

A series of 2- and 3-methoxyphenylpiperazine derivatives in combination with a 2-hydroxypropoxy linker and coumarins containing various substituents was synthesized and evaluated for antidepressant-like activity. Microwave-assisted synthesis was used, and the structures of all compounds were confirmed by ^1^H, ^13^C NMR, and HRMS spectrometry. The affinity toward the 5-HT_1A_ and 5-HT_2A_ receptors was determined using radioligand binding assays and analyzed by molecular docking studies. Among the compounds evaluated, four demonstrated high affinity for the 5-HT_1A_ receptor with the following Ki values: 5-(2-hydroxy-3-(4-(2-methoxyphenyl)piperazin-1-yl)propoxy)-4,7-dimethyl-2*H*-chromen-2-one (**5**) (90 nM), 6-acetyl-5-(2-hydroxy-3-(4-(2-methoxyphenyl)piperazin-1-yl)propoxy)-4,7-dimethyl-2*H*-chromen-2-one (**7**) (90 nM), 7-(2-hydroxy-3-(4-(3-methoxyphenyl)piperazin-1-yl) propoxy)-4-methyl-2*H*-chromen-2-one (**10**) (87 nM), and 8-acetyl-7-(2-hydroxy-3-(4-(2-methoxy phenyl)piperazin-1-yl)propoxy)-4-methyl-2*H*-chromen-2-one (**11**) (96 nM), and four demonstrated high affinity for the 5-HT_2A_ receptor with the following Ki values: 6-acetyl-7-(2-hydroxy-3-(4-(3-methoxyphenyl)piperazin-1-yl)propoxy)-4-methyl-2*H*-chromen-2-one (**2**) (83 nM), 8-acetyl-7-(2-hydroxy-3-(4-(3-methoxyphenyl)piperazin-1-yl)propoxy)-4-methyl-2*H*-chromen-2-one (**12**) (67 nM), 7-(2-hydroxy-3-(4-(2-methoxyphenyl)piperazin-1-yl) propoxy)-2*H*-chromen-2-one (**13**) (18 nM), and 7-(2-hydroxy-3-(4-(3-methoxyphenyl)piperazin-1-yl)propoxy)-2*H*-chromen-2-one (**14**) (68 nM). In functional assays, 8-acetyl-7-(2-hydroxy-3-(4-(2-methoxyphenyl) piperazin-1-yl)propoxy)-4-methyl-2*H*-chromen-2-one (compound **11**) exhibited a significant 5-HT_1A_ antagonistic profile. Computational studies revealed the structural details responsible for the high affinity of selected derivatives, which were compared to known 5HT_1A_ partial agonists.

## 1. Introduction

According to predictions from the World Health Organization, by 2030, depression will become the leading disorder worldwide [1]. Currently, depression, as a common mental illness, is considered the main cause of disability and death from suicide, and it can also cause a significant socioeconomic burden [2]. It is widely accepted that depression is a multifactorial disorder resulting from the interaction of psychological, biological, and social aspects, and its etiology remains unclear. Despite significant advances in the development of antidepressant drugs for the clinical treatment of depression, most of them still cause various side effects [3]. Therefore, the development of additional potential antidepressant drug molecules has become a major focal point in pharmaceutical research.

Serotonin, also known as 5-hydroxytryptamine (5-HT), belongs to a group of compounds called biogenic amines. It has been proven that a decrease in serotonin levels in the central nervous system contributes to a lowered mood and the onset of depressive states. Many brain functions, including mood, cognitive functions, and emotions, are modulated by the serotonin system (5-HT). Restoring the physiological level of this neurotransmitter is associated with mood improvement [4,5]. Currently, at least 15 major subtypes of the 5-HT receptor have been identified, and they are divided into seven classes (5-HT_1_ to 5-HT_7_) based on structural characteristics and G-protein-coupled signaling mechanisms [6,7]. 5-HT_1A_ receptors have been extensively studied for their role in modulating mood, anxiety, and cognitive functions and are divided into two distinct classes according to their location: 5-HT_1A_ autoreceptors and 5-HT_1A_ postsynaptic receptors [8]. Serotonin release is reduced by the decrease in the firing rate of serotoninergic neurons caused by the activation of 5-HT_1A_ autoreceptors [9,10].

The dysfunction of 5-HT_1A_ receptors plays a role in the pathogenesis of major depression. All treatment methods for depression with documented efficacy, including various classes of antidepressants and electroconvulsive therapy, indirectly or directly affect the activation of 5-HT_1A_ receptors. In individuals with depression who committed suicide, an increase in the number of presynaptic 5-HT_1A_ receptors in the raphe nuclei has been found [11]. Additionally, the main mechanism by which psychedelics such as LSD, psilocin, and mescaline work is through the activation of the 5-HT_2A_ receptor. The action of atypical antipsychotic drugs involves antagonizing this receptor, while the downregulation and desensitization of postsynaptic 5-HT_2A_ receptors occur with the use of antidepressants, indicating that these processes could play a role in their effectiveness against depression [12].

The *N*-heterocyclic piperazine ring is a moiety commonly found in biologically active compounds [13]. Recognized as a privileged structure in drug discovery, the piperazine scaffold is prevalent in biologically active compounds used in various therapeutic areas, such as antitumor, antibacterial, anti-inflammatory, antipsychotic, anti-Alzheimer, antifungal, and antidiabetic treatments [14,15,16,17,18,19,20,21,22]. Coumarin derivatives, whether from natural or synthetic sources, are valuable in various fields and are frequently used in medicine, often in the form of glycosides. Combining coumarin with an amino fragment in the form of piperazine significantly enhances the biological properties of these compounds compared to those of unsubstituted coumarins. The effects of coumarin–piperazine compounds on the central nervous system are well documented, as are their antibacterial, antitumor, antioxidant, and antiviral activities [23]. Ensaculin (KA-672 × HCl) has been identified as a compound with a unique pharmacological profile that affects the central nervous system (CNS) [24]. It has been shown that ensaculin (7-methoxy-6-{3-[4-(2-methoxyphenyl)-1-piperazinyl]propoxy}-3,4-dimethylcoumarin) has a high affinity for serotoninergic 5-HT_1A_ and 5-HT_7_ receptors, adrenergic α1 receptors, and dopaminergic D_2_ and D_3_ receptors. This compound also exhibits neuroprotective effects as an NMDA receptor channel blocker and inhibits acetylcholinesterase (AChE) activity in vitro [25]. Additionally, ensaculin has demonstrated memory-enhancing effects in passive and conditioned avoidance paradigms in both healthy rodents and those with experimentally induced amnesia [26,27]. The linker between the coumarin and piperazine moiety is also a very important structural element of this class of compounds with regard to their biological activity. Many scientific studies have shown that the affinities for 5-HT_1A_ receptors of propoxy- and butoxycoumarins linked to the N-arylpiperazinyl fragment can be very high: in the nanomolar range [23]. This affinity decreases with shortening or lengthening of the chain (2- and 5-carbon linkers) [28]. Another very important linker is the 2-hydroxypropyloxy chain, which is present in numerous marketed beta-adrenergic blocking drugs [29].

**Figure 1:**
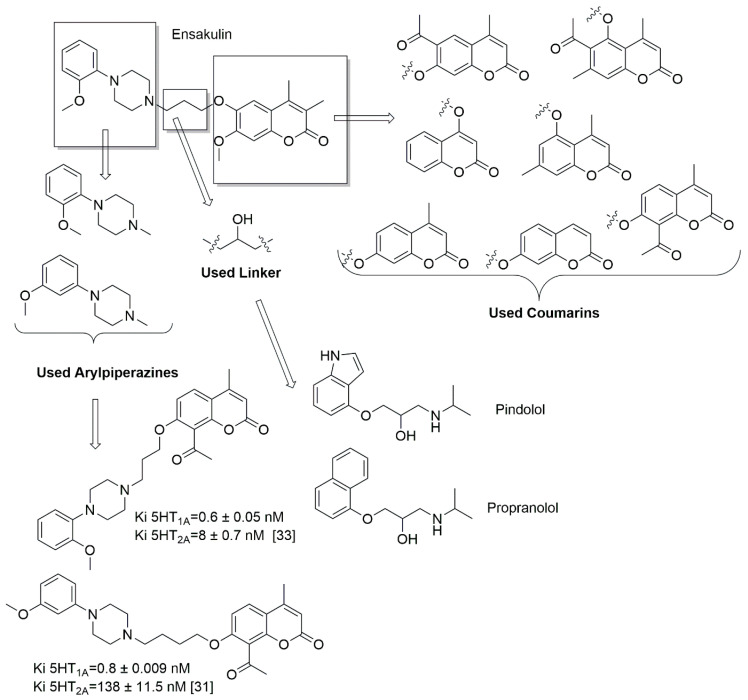
Design strategy of coumarin hybrids studied in this work.

As demonstrated in our previous studies and considering the structure of ensaculin, we see that coumarins linked by a three- or four-carbon linker had a high affinity for serotonin receptors [30,31,32,33,34]. Consequently, in this study, 2- and 3-methoxyphenylpiperazine in combination with a 2-hydroxypropoxy linker and coumarins containing various substituents were selected as building blocks for a new family of coumarin derivatives with expected antidepressant activity (Figure 1). In the scientific literature, there are only a few examples of such compounds. 4-Hydroxycoumarin derivatives with antibacterial activity and 6- and 7-hydroxycoumarin derivatives with antihistaminic activity have been previously reported [35,36], while derivatives of 8-chloro-4-methyl-7-hydroxycoumarin containing a 2-hydroxypropyl chain and a 4-methoxyphenylpiperazine fragment have been studied for their affinity to serotonin receptors but with unsatisfactory results [37]. Therefore, we believe that the synthesis and affinity studies for serotonin receptors of a series of these newly designed compounds can significantly enrich the library of biologically active coumarin derivatives.

## 2. Results and Discussion

### 2.1. Chemistry

**Scheme 1:**
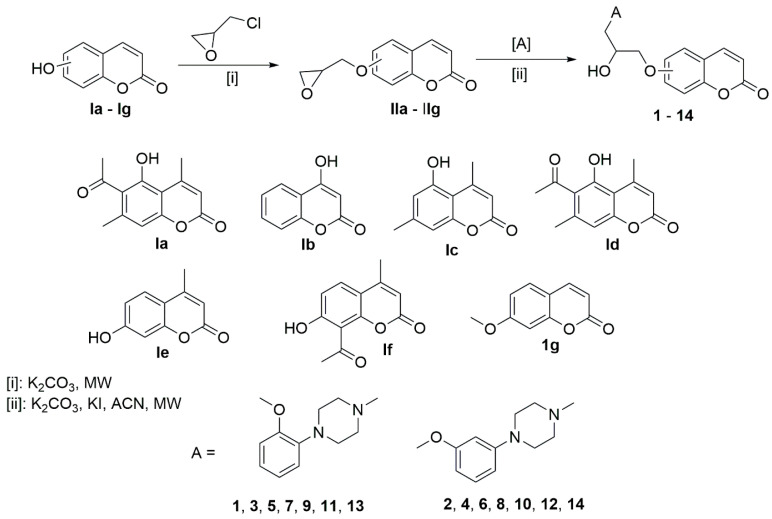
Synthetic route of new coumarins derivatives **1**–**14**.

All syntheses were carried out in two stages. In the first stage, the appropriate coumarin (**Ia**–**Ig**) was heated with epichlorohydrin in the presence of potassium carbonate (IV) for six cycles in a microwave reactor (Scheme 1) [38]. The progress of the reaction was monitored by TLC using silica gel plates (eluent:CHCl_3_, 10:0.25). The products were then purified by gravity column chromatography, gradually increasing the polarity of the mobile phase CHCl_3_:MeOH (from 100.0:0.0 to 100.0:1.0). The purified intermediate products **IIa**–**IIg** were then mixed with the appropriate amine, potassium carbonate, and a catalytic amount of KI with acetonitrile as the solvent. The mixture was again subjected to microwave heating for six cycles (each cycle consisting of three alternating phases of 6 min of heating at 80–85 °C and 2 min of cooling). All new compounds **1**–**14** synthesized in this work were synthesized using a microwave reactor and purified by column chromatography using silica gel. All compounds were characterized using ^1^H NMR, ^13^C NMR spectroscopy, and HRMS spectrometry. The NMR spectra for all compounds are presented in the Supplementary Data Figure S1.

### 2.2. Pharmacology

All new compounds **1**–**14** were subjected to affinity studies for the 5-HT_1A_ receptor using the radioligand binding assay with [3H]8-OH-DPAT, affinity studies for the 5-HT_2A_ receptor using the radioligand binding assay with [3H]ketanserin, and activity studies for the 5-HT_1A_ receptor using the [35S]GTPγS assay. Table 1 presents the affinity values of the tested compounds for the 5-HT_1A_ receptor. All additional information can be found in ESI (Supplementary Materials, Figures S2–S7). The compound with the significantly lowest binding affinity to the 5-HT1A receptor was 6-acetyl-5-(2-hydroxy-3-(4-(3-methoxyphenyl)piperazin-1-yl)propoxy)-4,7-dimethyl-2*H*-chromen-2-one (**8**), which has a methoxy group in the meta position of the phenyl ring, two methyl substituents and an acetyl group attached to the 2H-chromen-2-one system (Ki = 1776 nM). Modifying the structure of compound **8** by shifting the methoxy group to the ortho position resulted in nearly a 20-fold increase in affinity (Ki = 1776 nM for **8**, Ki = 90 nM for **7**, *p* < 0.0001). The remaining compounds exhibited comparable affinity with Ki values ranging from 87 nM to 264 nM.

Table 1 presents the results of the antagonistic activity studies against the 5-HT_1A_ receptor. The compound demonstrating the highest potency was 8-acetyl-7-(2-hydroxy-3-(4-(2-methoxyphenyl)piperazin-1-yl)propoxy)-4-methyl-2*H*-chromen-2-one (**11**) (IC_50_ = 43 nM, *p* < 0.0001), which features a methoxy substituent in the ortho position of the phenyl ring, a coumarin core substituted with a methyl group in position C-4, and an acetyl group in position C-8. Shifting the methoxy substituent to the meta position resulted in more than a 140-fold decrease in potency (IC_50_ = 6.155 µM, *p* < 0.0001 for 8-acetyl-7-(2-hydroxy-3-(4-(3-methoxyphenyl)piperazin-1-yl)propoxy)-4-methyl-2*H*-chromen-2-one (**12**)). The presence of the acetyl group in the 4-methyl-2*H*-chromen-2-one ring contributed to the greater antagonistic potency of compound **11** (IC_50_ = 0.043 µM) compared to the derivative without the acetyl substituent (IC_50_ = 2.416 µM, *p* < 0.0005 for 7-(2-hydroxy-3-(4-(2-methoxyphenyl)piperazin-1-yl)propoxy)-4-methyl-2*H*-chromen-2-one (**9**)). Derivatives with a coumarin ring substituted with a methyl group at the C-4 position and an acetyl group at the C-6 position—namely, 6-acetyl-7-(2-hydroxy-3-(4-(2-methoxyphenyl)piperazin-1-yl)propoxy)-4-methyl-2*H*-chromen-2-one (**1**) and 6-acetyl-7-(2-hydroxy-3-(4-(3-methoxyphenyl)piperazin-1-yl)propoxy)-4-methyl-2*H*-chromen-2-one (**2**)—along with derivatives with an unsubstituted 2*H*-chromen-2-one ring, such as 7-(2-hydroxy-3-(4-(3-methoxyphenyl)piperazin-1-yl)propoxy)-2*H*-chromen-2-one (**14**) and 4-(2-hydroxy-3-(4-(2-methoxyphenyl)piperazin-1-yl)propoxy)-2*H*-chromen-2-one (**3**)—and 7-(2-hydroxy-3-(4-(3-methoxyphenyl)piperazin-1-yl)propoxy)-4-methyl-2*H*-chromen-2-one (**10**) with a 4-methyl-2*H*-chromen-2-one structure—were found to have the weakest antagonistic activity. The IC_50_ values for these derivatives were 330.7 µM, 58.5 µM, 112 µM, 67.75 µM, and 89.49 µM (*p* < 0.0001), respectively. For compounds with a methoxy group in the ortho position and a coumarin moiety with a methyl substituent at the C-4 position (**9**), the addition of an acetyl group at the C-6 position (**1**) resulted in a significant, over 100-fold decrease in antagonistic activity against the receptor (IC_50_ = 2.4 µM vs. IC_50_ = 330.7 µM, *p* < 0.0001). Modification of the methoxy group position to meta (compound **10**) in the phenyl ring, without altering the 4-methyl-2*H*-chromen-2-one ring, resulted in a decrease in activity (IC_50_ = 2.4 µM vs. IC_50_ = 89.49 µM, *p* = 0.0021). The addition of an acetyl group at the C-8 position of the heterocyclic system in compound **10** enhanced the antagonistic potency (IC_50_ = 6.155 µM for compound **12**, IC5_0_ = 89.49 µM for compound **10**, *p* < 0.05). Shifting the methoxy group from the ortho to meta position in the phenyl ring, for 8-acetyl-7-(2-hydroxy-3-(4-(2-methoxyphenyl)piperazin-1-yl)propoxy)-4-methyl-2*H*-chromen-2-one (**11**) and 8-acetyl-7-(2-hydroxy-3-(4-(3-methoxyphenyl)piperazin-1-yl)propoxy)-4-methyl-2*H*-chromen-2-one (**12**), had a similar effect and resulted in a decrease in activity (IC_50_ = 6.155 µM for compound **11**, IC_50_ = 0.043 µM for compound **12**, *p* < 0.0001).

Table 1 also presents the affinity values of the tested compounds for the 5-HT_2A_ receptor. One-way analysis of variance (ANOVA) revealed that compounds with the unsubstituted 2H-chromen-2-one ring (**13** and **14**), as well as those in which the coumarin moiety is substituted with one or two methyl groups and contains an acetyl group at the C-6 or C-8 position of the ring, including **2** (6-acetyl-4-methyl-2*H*-chromen-2-one), **8** (6-acetyl-4,7-dimethyl-2*H*-chromen-2-one), and **12** (8-acetyl-4-methyl-2*H*-chromen-2-one), exhibited the strongest affinity for the 5-HT_2A_ receptor. Four out of the five listed compounds had a methoxy substituent in the meta position of the phenyl ring. Only 7-(2-hydroxy-3-(4-(2-methoxyphenyl)piperazin-1-yl)propoxy)-2*H*-chromen-2-one (**13**) was a high-affinity derivative with the methoxy group in the ortho position. The Ki values for these compounds were as follows: 18 nM, 68 nM, 83 nM, 115 nM, and 67 nM (F = 15.27, *p* < 0.0001). For compounds with the 6-acetyl-4-methyl-2*H*-chromen-2-one ring, such as 6-acetyl-7-(2-hydroxy-3-(4-(2-methoxyphenyl)piperazin-1-yl)propoxy)-4-methyl-2*H*-chromen-2-one (**1**) and 6-acetyl-7-(2-hydroxy-3-(4-(3-methoxyphenyl)piperazin-1-yl)propoxy)-4-methyl-2*H*-chromen-2-one (**2**), shifting the methoxy group in the phenyl ring from the ortho to the meta position significantly increased affinity (**1**: Ki = 1846 nM vs. **2**: Ki = 83 nM, *p* = 0.0002). Similar trends were observed in the group of derivatives with the 8-acetyl-4-methyl-2*H*-chromen-2-one ring: for 8-acetyl-7-(2-hydroxy-3-(4-(2-methoxyphenyl)piperazin-1-yl)propoxy)-4-methyl-2*H*-chromen-2-one (**11**) and 8-acetyl-7-(2-hydroxy-3-(4-(3-methoxyphenyl)piperazin-1-yl)propoxy)-4-methyl-2*H*-chromen-2-one (**12**). In these compounds, shifting the methoxy group from the ortho to meta position resulted in a 100-fold increase in affinity for the 5-HT_2A_ receptor with Ki values improving from 6157 nM to 67 nM (*p* = 0.0012). For the pair of compounds 6-acetyl-5-(2-hydroxy-3-(4-(2-methoxyphenyl)piperazin-1-yl)propoxy)-4,7-dimethyl-2*H*-chromen-2-one (**7**) and 6-acetyl-5-(2-hydroxy-3-(4-(3-methoxyphenyl)piperazin-1-yl)propoxy)-4,7-dimethyl-2*H*-chromen-2-one (**8**) with the 6-acetyl-4,7-dimethyl-2*H*-chromen-2-one group, the same change in the position of the methoxy position led to a 40-fold increase in affinity (Ki = 115 nM for **8** and Ki = 4150 nM for **7**, *p* = 0.0004). On the other hand, modifying the position of the methoxy group on the phenyl ring did not affect the binding affinity of compounds **13** and **14**, which have an unmodified chromen-2-one core. The weakest affinity for the 5-HT_2A_ receptor was observed in compounds with a methoxy group in the ortho position and a coumarin moiety substituted with one or two methyl groups at positions C-4 and C-7, respectively, along with an acetyl group at positions C-6 or C-8. These compounds are labeled as **7** (6-acetyl-4,7-dimethyl-2*H*-chromen-2-one), **1** (6-acetyl-4-methyl-2*H*-chromen-2-one), and **11** (8-acetyl-4-methyl-2*H*-chromen-2-one). Additionally, one derivative with a methoxy group in the meta position, labeled **5** (4,7-dimethyl-2*H*-chromen-2-one), also exhibited weak affinity. For the pair of compounds **5** and **6** with the 4,7-dimethyl-2*H*-chromen-2-one structure, modification of the methoxy group position in the phenyl ring from ortho to meta also resulted in increased affinity. In this case, a nearly two-fold increase was observed with Ki values rising from 1182 nM to 662 nM (*p* = 0.0317). The addition of an acetyl group at position C-6 to 4,7-dimethoxy-2*H*-chromen-2-one did not significantly affect the binding affinity to the 5-HT_2A_ receptor.

Substituting the 4-methyl-2*H*-chromen-2-one ring with an acetyl group at position C-6 led to a decrease in receptor binding affinity with Ki values dropping from 291 nM for 7-(2-hydroxy-3-(4-(2-methoxyphenyl)piperazin-1-yl)propoxy)-4-methyl-2*H*-chromen-2-one (**9**) to 1846 nM for 6-acetyl-7-(2-hydroxy-3-(4-(2-methoxyphenyl)piperazin-1-yl)propoxy)-4-methyl-2*H*-chromen-2-one (**1**) (*p* = 0.0197). When an acetyl group was added at position C-8 to the 4-methyl-2*H*-chromen-2-one ring, the receptor affinity for compounds with the methoxy substituent in the ortho position increased (Ki = 6157 nM for compound **11**, Ki = 291 nM for compound **9**, *p* < 0.001). However, a similar structural modification for compounds with the methoxy group in the meta position of the phenyl ring did not have a statistically significant impact on receptor binding affinity (*p* = 0.0878). On the contrary, the introduction of a methyl group into the coumarin ring reduced receptor binding affinity with Ki values decreasing from 68 nM for compound **14** to 260 nM for compound **10** (*p* = 0.0483) and from 18 nM for compound **13** to 291 nM for compound **9** (*p* = 0.0051).

The above results suggest that not only the presence of an acetyl group in the coumarin structure is a crucial factor that modulates the biological activity of these compounds, but also the specific position of the acetyl group within the molecule is significant. In previous experiments, a similar change in the position of the acetyl group in the coumarin ring from position C-8 to C-6 resulted in a decrease in affinity for derivatives with a methoxy substituent in the ortho position, from Ki = 1.0 nM to Ki = 5.75 nM, and for the meta position from Ki = 0.8 nM to Ki = 12.9 nM [34]. It is worth noting that in the study mentioned above that replacing the methoxy group with a bromine atom resulted in a significant increase in affinity (Ki = 0.78 nM, Ki = 12.9 nM). This suggests that a halogen substituent in the phenyl ring could be more advantageous in terms of affinity and activity toward the 5-HT_1A_ receptor. In our previous study cited above, the compound 6-acetyl-7-{4-[4-(3-methoxyphenyl)piperazin-1-yl]butoxy}-4-methylcoumarin acted as an agonist for the 5-HT_1A_ receptor. Interestingly, modification of the methoxy group to the ortho position resulted in a change in receptor activity to antagonist (IC_50_ = 301 nM) [34]. It is possible that a similar phenomenon occurs for the acetyl derivatives studied in this work.

The compounds examined in this study demonstrated an affinity for the 5-HT_1A_ receptor and exhibited weak antagonist activity toward this receptor with IC_50_ values in the micromolar range. In the study of derivatives of 8-acetyl-7-hydroxy-4-methylcoumarin, compounds with a methoxy or bromine substituent in the ortho position of the phenyl ring were identified as potent antagonists of the 5-HT_1A_ receptor. The compound 8-acetyl-7-{3-[4-(2-methoxyphenyl) piperazin-1-yl]propoxy}-4-methylcoumarin showed an affinity of Ki = 0.60 nM for the 5-HT_1A_ receptor and Ki = 8 nM for the 5-HT_2A_ receptor [33]. For comparison, 8-acetyl-7-(2-hydroxy-3-(4-(2-methoxyphenyl)piperazin-1-yl)propoxy)-4-methyl-2*H*-chromen-2-one (**11**) exhibited an affinity of Ki = 6157 nM for the 5-HT_1A_ receptor and Ki = 90 nM for the 5-HT_2A_ receptor with antagonist activity against 5-HT_1A_ at an IC_50_ of 0.043 µM. The difference between compound **11** and the derivative mentioned in the earlier study is the presence of a hydroxyl group at position C-2 in the linker of compound **11**. This suggests that the presence of a hydroxyl group in the linker negatively impacts the affinity and activity toward the examined serotonergic receptors. For the 5-HT_2A_ receptor, the strongest affinity was observed for compounds with an unsubstituted 2H-chromen-2-one ring and those with acetyl and methyl groups in specific positions. Four out of five derivatives with the highest affinity for the 5-HT_2A_ receptor had a methoxy group in the meta position of the phenyl ring. The exception was compound **13** (Ki = 18 nM), which had a methoxy group in the ortho position and exhibited a stronger affinity than its analogue **14** (Ki = 68 nM) with a methoxy group in the meta position. Unlike the other high-affinity derivatives, **13** and **14** lack methyl and acetyl substituents on the 2H-chromen-2-one ring. The introduction of a methyl group into the coumarin ring reduced binding strength. Conversely, Gonzalez-Gomez et al. demonstrated that the affinity of aryloxyalkyl derivatives of coumarins for the 5-HT_1A_ and 5-HT_2A_ receptors is highest for compounds with an o-methoxyphenyl substituent, whereas substitution of the phenyl group with pyrimidine or pyridine reduces affinity [39]. The modification of introducing methyl substituents into the coumarin ring had less impact on the receptor profile of the studied compounds. These substitutions did not affect the affinity and activity toward 5-HT_1A_. On the other hand, the modification of adding a methyl group to the coumarin ring at position C-3 increased the 5-HT_1A_ affinity almost 7-fold compared to the unsubstituted derivatives and those with methyl groups at positions C- 3 and C-4 [40]. However, when assessing binding to 5-HT_2A_, it was found that introducing a methyl group into the unsubstituted coumarin ring decreased the binding strength to the receptor both for compounds with a methoxy group in the meta position of the phenyl ring (Ki = 68 nM for **14** to Ki = 260 nM for **10**) and for compounds with a methoxy group in the ortho position (Ki = 18 nM for **13** to Ki = 291 nM for **9**).

### 2.3. ADMET and Molecular Docking Studies

The predicted ADMET properties of the studied compounds are presented in Table 2. All coumarin derivatives fall within Lipinski’s rule of five, suggesting their high druglikeness [41]. All of the tested compounds have a higher number of hydrogen bond acceptors compared to aripiprazole, suggesting that they can form more favorable interactions with the receptors binding sites as well as higher solubility in water. Also, in all cases, the nitrogen atom of the piperazine part of ligands is predicted to be basic and protonated in physiological conditions. Finally, the predicted LD_50_ values are within the 500–1100 mg/kg value, which is similar to aripiprazole and other known 5HT antagonists/agonists. Additional toxicity predictions suggest that all tested compounds may display high immunotoxocity, respiratory toxicity and neurotoxicity, also similarly to the structurally similar aripiprazole.

**Figure 2:**
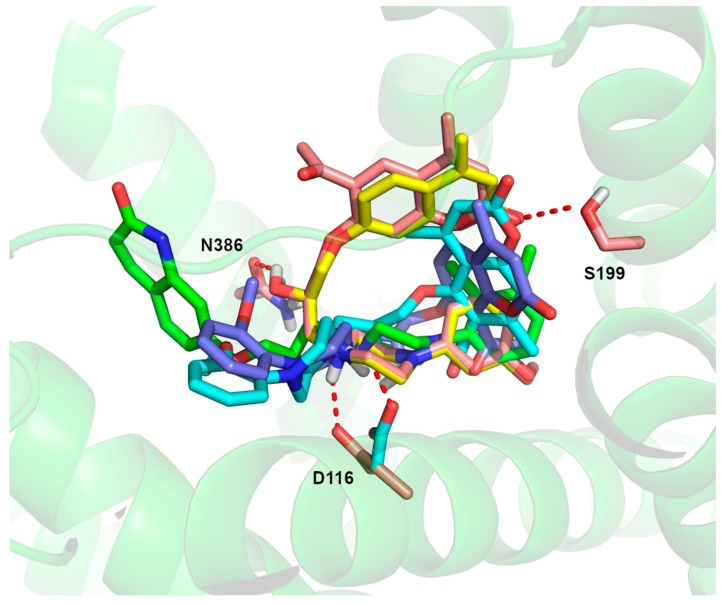
Computationally obtained binding poses of **2** (pink), **7** (cyan), **10** (yellow) and **11** (purple) to the 5HT_1A_ receptor compared to the experimental pose of aripiprazole (green).

The predicted Gibbs free energies of binding (ΔG_binding_) and Ki values are presented in Table 3 and are in general within the expected accuracy of the computational approach used. All the derivatives studied showed high affinities to both the 5HT_1A_ and the 5HT_2A_ receptor models similarly to our previous studies on related derivatives. In the case of the 5HT_1A_ receptor, the highest Gibbs free energy of binding was found for derivative **7** with the −10.0 kcal/mol value, which is slightly lower than that estimated for aripiprazole. Also, it is worth mentioning that the experimental Ki values for compound **7** indicated a very strong binding to the 5HT_1A_ receptor, which shows good agreement between the experimental and computational results (within the expected accuracy of 1 kcal/mol). Figure 2 shows the obtained poses of **7** and **2** (a derivative with the second highest estimated affinity to 5HT_1A_ receptor, but experimentally found to bind to the 5HT_1A_ receptor, which is much weaker) compared to the experimental pose of aripiprazole from the crystal structure (PDB id: 7e2z). One can see that **7** is predicted to occupy almost the exact same space as aripiprazole, but the entire molecule is rotated by 180 degrees with respect to aripiprazole with the coumarin core being on the same side of the binding pocket as the 2,3-dichlorophenyl group of aripiprazole. On the other hand, **2** is predicted to adopt a different conformation, and the high flexibility of the linker positions the coumarin core in a different part of the binding pocket with a hydrogen bond to S199. Figure 2 also shows the obtained poses of derivatives **10** and **11** with high experimental affinities to the 5HT_1A_ receptor. Interestingly, the predicted pose of **10** is almost identical to that of **2**, while the predicted pose of **11** is similar to that of **7** despite the fact that all four ligands are anchored in the binding site via a salt bridge to D116. Based on these results, we can suggest that there are two possible binding poses of this series of coumarin derivatives, depending on the subtle changes in their structures.

As in our previous studies, the predicted affinities to the 5HT_2A_ receptor are higher than for the 5HT_1A_ receptor by around 1 order of magnitude, but they are also substantially lower than the predicted affinity of ketanserin, which is a known 5HT_2A_ receptor antagonist. At this point, we do not have a good explanation of the discrepancies between the experimental and theoretical results for this receptor [34].

All additional information can be found in the ESI (Supplemetary Materials, Tables S1–S6).

## 3. Materials and Methods

### 3.1. Experimental Section

#### 3.1.1. Chemical Compounds

The starting materials were obtained from Aldrich or Merck and used as received. The reaction processes were conducted using a Plazmatronika-Poland 1000 microwave oven (Wrocław, Poland), and the melting points were measured with an ElectroThermal 9001 Digital Melting Point apparatus (Chelmsford, UK) with values uncorrected. Highresolution mass spectra were obtained using a Shimadzu LCMS-9030 spectrometer (Kyoto, Japan). ^1^H NMR and ^13^C NMR spectra in solution were recorded at 25 °C with a Bruker Advance III HD 300 MHz spectrometer (Karlsruhe, Germany) employing standard Topspin v. 3.2 software. Chemical shifts δ [ppm] were referenced to TMS. TLC was conducted on Kieselgel 60 F254 plates (Sigma-Aldrich, Oakville, ON, Canada), and the spots were detected under UV light at wavelengths of 254 and 365 nm. The ^1^H NMR and ^13^C NMR spectra of all synthesized compounds are available in the ESI (Supplemetary Materials, Figure S1).

#### 3.1.2. General Procedure for Preparing Compounds **1**–**14**

##### General Procedure for Preparing Intermediate Derivatives **IIa**–**IIg**

Compounds **IIa**–**IIg** bearing the (oxiran-2-yl)methoxy group were prepared by the previously reported procedures (Scheme 1) [38].

##### General Procedure for Preparing Derivatives **1**–**14**

The corresponding intermediate product (**IIa**–**IIg**) (1 mmol), the appropriate phenylpiperazine (2 mmol), K_2_CO_3_ (0.4 mmol), a catalytic amount of KI, and acetonitrile (5 mL) were added to the flask. The mixture was heated at 80–85 °C in a monomode microwave oven (300 W; number of cycles: 3; heating time: 6 min per cycle; total heating time: 18 min). The reaction progress was monitored by TLC on silica gel plates (eluent: CHCl_3_:MeoH; 10:0.25). Upon completion of the reaction, the hot mixture was filtered, and the solvent was evaporated. The residue was purified by column chromatography (eluent: CHCl_3_:MeOH; 100:1.0), yielding the final products **1**–**14**. Atom numbering, ^1^H NMR and ^13^C NMR spectra are available in the ESI (Supplemetary Materials, Figure S1).

##### 6-Acetyl-7-(2-hydroxy-3-(4-(2-methoxyphenyl)piperazin-1-yl)propoxy)-4-methyl-2*H*-chromen-2-one (**1**)

m.p.: 151–152 °C, Rf = 0.24, yield 21%, ^1^H NMR (300 MHz, CDCl_3_) δ ppm: 8.03 (s, 1H, H-5), 7.09–6.89 (m, 5H, H-8, H3″-H6″), 6.17 (s, 1H, H-3), 4.55 (br. s, 1H, H-2′), 4.22 (d, *J* = 3 Hz, 2H, H-1′), 3.90 (s, 3H, H-7″), 3.31–2.91 (m, 10H, H3p+5p, H2p+6p, H3′), 2.70 (s, 3H, H-11), 2.44 (s, 3H, H-9); ^13^C NMR (300 MHz, CDCL_3_) δ ppm: 197.5 (C-10), 160.8 (C-1″), 160.5 (C-7), 157.6 (C-2), 152.9 (C-8a), 152.3 (C-4), 140.3 (C-2″), 128.1 (C-5), 124.8 (C-6), 123.8 (C-6″), 121.2 (C-5″), 118.6 (C-4″), 113.7 (C-3″), 113.1 (C-3), 111.3 (C-4a), 101.2 (C-8), 71.5 (C-1′), 64.8 (C-2′), 60.9 (C-3′), 55.6 (C3p+5p), 54.1 (C2p+6p), 49.7 (C-7″), 32.2 (C-11), 18.9 (C-9); TOF MS ES+: [M + H]^+^ calcd. for C_26_H_30_O_6_N_2_: 467.2176 found 467.2189.

##### 6-Acetyl-7-(2-hydroxy-3-(4-(3-methoxyphenyl)piperazin-1-yl)propoxy)-4-methyl-2*H*-chromen-2-one (**2**)

m.p.: 165 °C, Rf = 0.70, yield 83%, ^1^H NMR (300 MHz, CDCl_3_) δ ppm: 8.07 (s, 1H, H-5), 7.18 (t, *J* = 4.5 Hz, 1H, H-5″), 6.88 (s, 1H, H-8), 6.62 (d, *J* = 6 Hz, 1H, H6″), 6.47 (s, 1H, H-2″, 6.44 (d, *J* = 3 Hz, 1H, H-4″), 6.19 (s, 1H, H-3), 4.25–2.18 (m, 2H, H-1′), 4.14–4.11 (m, 1H, H-2′), 3.80 (s, 3H, H-7″), 3.25–3.22 (m, 4H, H3p+5p), 2.89–2.85 (m, 2H, H3′), 2.70 (s, 3H, H-11), 2.67–2.58 (m, 4H, H2p+6p), 2.41 (s, 3H, H-9); ^13^C NMR (300 MHz, CDCl_3_) δ ppm: 197.5 (C-10), 160.8 (C-1″), 160.5 (C-7), 160.2 (C-2), 157.4 (C-8a), 152.6 (C-4), 152.4 (C-2″), 129.8 (C-5), 128.0 (C-6), 124.9 (C-6″), 113.6 (C-5″), 112.9 (C-4″), 108.9 (C-3″), 104.6 (C-3), 102.7 (C-4a), 100.6 (C-8), 71.5 (C-1′), 65.1 (C-2′), 60.3 (C-3′), 55.1 (C-3p, C-5p), 53.2 (C-2p, C-6p), 49.1 (C-7″), 32.1 (C-11), 18.7 (C-9); TOF MS ES+: [M + H]^+^ calcd. for C_26_H_36_O_5_N_2_: 467.2176 found 467.2182.

##### 4-(2-Hydroxy-3-(4-(2-methoxyphenyl)piperazin-1-yl)propoxy)-2*H*-chromen-2-one (**3**)

m.p.: 133–135 °C, Rf = 0.27, yield 89%, ^1^H NMR (300 MHz, CDCl3) δ ppm: 7.87 (dd, *J*_1_ = 3Hz, *J*_2_ = 9 Hz, 1H, H-5), 7.59–7.53 (m, 1H, H-7), 7.33–7.28 (m, 2H, H6, H8), 7.07–6.88 (m, 4H, H-3″-H-6″), 5.73 (s, 1H, H-3), 4.36–4.29 (m, 1H, H-2′), 4.22–4.15 (m, 2H, H-1′), 3.88 (s, 3H, H-7″), 3.17 (br. s, 4H, H3p+5p), 3.01 (m, 2H, H-3′), 2.73–2.66 (m, 4H, H2p+6p); ^13^C NMR (300 MHz, CDCl3) δ ppm: 165.6 (C-4), 162.9 (C-2), 153.4 (C-1″), 152.3 (C-8a), 140.8 (C-2″), 132.7 (C-7), 124.1 (C-6), 123.5 (C-5), 123.3 (C-6″), 121.1 (C-5″), 118.4 (C-4″), 116.9 (C-8), 115.6 (C-4a), 111.3 (C-3″), 90.9 (C-3), 71.3 (C-1′), 64.8 (C-2′), 60.3 (C-3′), 55.5 (C3p+5p), 53.7 (C-7″), 50.5 (C2p+6p); TOF MS ES+: [M + H]^+^ calcd. for C_23_H_26_O_5_N_2_: 411.1914 found 411.1928.

##### 4-(2-Hydroxy-3-(4-(3-methoxyphenyl)piperazin-1-yl)propoxy)-2*H*-chromen-2-one (**4**)

Oil, Rf = 0.58, yield 96%, ^1^H NMR (300 MHz, CDCl3) δ ppm: 7.85 (d, *J* = 3 Hz, 1H, H-5), 7.56 (t, *J* = 5.1 Hz, 1H, H-7), 7.33 (d, *J* = 10 Hz, 1H, H6), 7.28 (d, *J* = 10 Hz, 1H, H-8), 7.18 (t, *J* = 5 Hz, 1H, H-5″), 6.54 (d, *J* = 6.3 Hz, 1H, H-6″), 6.47 (t, *J* = 1.3 Hz, 1H, H-2″), 6.43 (d, *J* = 1.2 Hz, 1H, H-4″), 5.73 (s, 1H, H-3), 4.28–4.24 (m, 1H, H-2′), 4.19–4.13 (m, 2H, H-1′), 3.79 (s, 3H, H-7″), 3.28–3.20 (m, 4H, H3p+5p), 2.89–2.85 (m, 2H, H-3′), 2.68–2.60 (m, 4H, H2p+6p); ^13^C NMR (300 MHz, CDCl3) δ ppm: 165.4 (C-4), 162.7 (C-2), 160.5 (C-3″), 153.2 (C-8a), 152.3 (C-1″), 132.4 (C-5″), 129.8 (C-7), 123.8 (C-5), 122.9 (C-6), 116.7 (C-8), 115.5(C-4a), 108.9 (C-4″), 104.6 (C-6″), 102.6 (C-8″), 90.9 (C-3), 71.2 (C-1′), 64.8 (C-2′), 60.0 (C-3′), 55.1 (C3p+5p), 53.2 (C-7″),49.1 (C2p+6p); TOF MS ES+: [M + H]^+^ calcd. for C_23_H_26_O_5_N_2_: 411.1914 found 411.1928.

##### 5-(2-Hydroxy-3-(4-(2-methoxyphenyl)piperazin-1-yl)propoxy)-4,7-dimethyl-2*H*-chromen-2-one (**5**)

m.p.: 150–152 °C, Rf = 0.20, yield 94%, ^1^H NMR (300 MHz, CDCl3) δ ppm: 7.09–7.03 (m, 1H, H-5″), 6.98–6.95 (m, 2H, H3″, H6″), 6.95 (br. s, 1H, H-4″), 6.78 (s, 1H, H-8), 6.56 (s, 1H, H-6), 6.07 (s, 1H, H-3), 4.38–4.34 (m, 1H, H-2′), 4.10–4.09 (m, 2H, H-1′), 3.89 (s, 3H, H-7″), 3.24 (br. s, 4H, H3p+5p), 3.11–3.08 (m, 2H, H-3′), 2.90–2.80 (m, 4H, H2p+6p), 2.63 (s, 3H, H-10), 2,40 (s, 3H, H-9); ^13^C NMR (300 MHz, CDCl3) δ ppm: 161.0 (C-1″), 156.9 (C-2), 155.4 (C-4), 154.0 (C-5), 152.3 (C-8a), 143.2 (C-2″), 140.5 (C-7), 123.7 (C-6″), 121.2 (C-4″), 118.5 (C-5″), 113.8 (C-4a), 111.3 (C-3″), 110.8 (C-3), 108.4 (C-6), 108.3 (C-8), 71.3 (C-1′), 65.0 (C-2′), 61.2 (C-3′), 55.6 (C3p+5p), 53.9 (C2p+6p), 50.1 (C-7″), 24.9 (C-10), 22.2 (C-11); TOF MS ES+: [M + H]^+^ calcd. for C_25_H_30_O_5_N_2_: 439.2227 found 439.2224.

##### 5-(2-Hydroxy-3-(4-(3-methoxyphenyl)piperazin-1-yl)propoxy)-4,7-dimethyl-2*H*-chromen-2-one (**6**)

m.p.: 117–131 °C, Rf = 0.71, yield 55%, ^1^H NMR (300 MHz, CDCl3) δ ppm: 7.18 (t, *J* = 8.1 Hz, 1H, H-5″), 6.71 (s, 2H, H-6, H-8), 6.56–6.53 (m, 2H, H-6″, H-2″), 6.03–5.98 (m, 2H, H-3, H-4″), 4.35–4.28 (m, 1H, H-2′), 4.22–4.13 (m, 2H, H-1′), 3.79 (s, 3H, H-7″), 3.26 (br. s, 4H, H-3p+H-5p), 2.60–2.56 (m, 10H, H-2p+H-6p, H-9, H-10), 2.38 (s, 2H, H-3′); ^13^C NMR (300 MHz, CDCl_3_, δ ppm): 160.5 (C-3″), 156.9 (C-2), 154.2 (C-4), 153.8 (C-5), 152.4 (C-1″), 143.1 (C-7), 143.1 (C-5″), 129.8 (C-4a), 113.5 (C-6, C-8a), 110.7 (C-3), 108.9 (C-4″), 104.6 (C-8), 102.6 (C-6, C-2), 71.3 (C-1′), 69.6 (C-2′), 65.3 (C-3′), 55.1 (C-3p+C-5p), 53.2 (C-2p+C-6p), 49.1 (C-7″), 24.5 (C-10), 21.9 (C-9); TOF MS ES+: [M + H]^+^ calcd. for C_25_H_30_O_5_N_2_: 439.2227 found 439.2220.

##### 6-Acetyl-5-(2-hydroxy-3-(4-(2-methoxyphenyl)piperazin-1-yl)propoxy)-4,7-dimethyl-2*H*-chromen-2-one (**7**)

Oil, Rf = 0.40, yield 95%, ^1^H NMR (300 MHz, CDCl3) δ ppm: 6.99–6.86 (m, 5H, H-8, H3″-H-6″), 6.18 (s, 1H, H-3), 4.15–4.07 (m, 1H, H-2′), 3.87–3.80 (m, 5H, H-7″, H-1′), 3.13 (br. s, 4H, H3p+5p), 2.94–2.90 (m, 2H, H-3′), 2.71–2.61 (m, 7H, H2p+6p, H-12), 2.59 (s, 3H, H-10), 2.30 (s, 3H, H-9); ^13^C NMR (300 MHz, CDCl_3_, δ ppm): 204.8 (C-11), 160.1 (C-1″), 154.7 (C-2), 153.9 (C-5), 152.9 (C-8a), 152.2 (C-4), 140.8 (C-7), 139.2 (C-2″), 133.5 (C-4a), 123.3 (C-6″), 121.1 (C-4″), 118.3 (C-5″), 116.0 (C-6), 115.4 (C-3″), 112.7 (C-3), 111.2 (C-8), 65.3 (C-1′), 59.8 (C-2′), 55.5 (C-3′), 53.5 (C3p+5p), 50.5 (C2p+6p, C-7″), 32.9 (C-12), 22.9 (C-10), 19.4 (C-9); TOF MS ES+: [M + H]^+^ calcd. for C_27_H_32_O_6_N_2_: 481.2333 found 481.2348.

##### 6-Acetyl-5-(2-hydroxy-3-(4-(3-methoxyphenyl)piperazin-1-yl)propoxy)-4,7-dimethyl-2*H*-chromen-2-one (**8**)

Oil, Rf = 0.48, yield 81%, ^1^H NMR (300 MHz, CDCl3) δ ppm: 7.19 (t, *J* = 4.9 Hz, 1H, H-5″), 6.99 (s, 1H, H-8), 6.54 (dd, *J*_1_ = 0.5 Hz, *J_2_* = 5 Hz, 1H, H-6″), 6.46 (t, *J* = 1.3 Hz, 1H, H-4″), 6.44 (d, *J* = 3 Hz, 1H, H-2″), 6.18 (s, 1H, H-3), 3.87–3.79 (m, 5H, H-1′, H-7″), 3.25–3.17 (m, 4H, H3p+5p), 2.84–2.80 (m, 2H, H-3′), 2.67 (s, 3H, H-10), 2.61–2.37 (m, 7H, H2p+6p, H-12), 2.30 (s, 3H, H-9); ^13^C NMR (300 MHz, CDCl_3_, δ ppm): 204.6 (C-11), 160.5 (C-1″), 159.9 (C-2), 154.5 (C-5), 153.7 (C-8a), 152.4 (C-4), 152.3 (C-7), 139.0 (C-3″), 133.4 (C-4a), 129.7 (C-8″), 115.8 (C-5″), 115.3 (C-6), 112.5 (C-3), 108.8 (C-6″), 104.6 (C-4″), 102.6 (C-6″), 80.2 (c-2″). 65.4 (C-1′), 59.6 (C-2′), 55.1 (C-3′), 53.0 (C3p+5p), 49.1 (C2p+6p, C-7″), 32.7 (C-12), 22.7 (C-10), 19.2 (C-9); TOF MS ES+: [M + H]^+^ calcd. for C_27_H_32_O_6_N_2_: 481.2333 found 481.2343.

##### 7-(2-Hydroxy-3-(4-(2-methoxyphenyl)piperazin-1-yl)propoxy)-4-methyl-2*H*-chromen-2-one (**9**)

m.p.: 110–112 °C, Rf = 0.80, yield 50%, ^1^H NMR (300 MHz, CDCl3) δ ppm: 7.50 (d, *J* = 9 Hz, 1H, H-5), 7.28–6.85 (m, 6H, H-6, H-8, H3″- H6″), 6.15 (s, 1H, H-3), 4.27–4.20 (m, 1H, H-2′), 4.13–4.03 (m, 2H, H-1′), 3.88 (s, 3H, H-7″), 3.16 (br. s, 4H, H3p+5p), 3.00–2.96 (m, 4H, H2p+6p), 2.77–2.73 (m, 2H, H-3′), 2.41 (s, 3H, H-9); ^13^C NMR (300 MHz, CDCl_3_, δ ppm): 161.8 (C-1″), 161.4 (C-2), 155.3 (C-7), 152.7 (C-8a), 152.3 (C-4), 140.8 (C-2″), 125.8 (C-5), 123.5 (C-6″), 121.2 (C-4″), 118.5 (C-5″), 114.1 (C-3″), 112.7 (C-6), 112.3 (C-3), 111.3 (C-4a), 101.8 (C-8), 70.7 (C-1′), 65.2 (C-2′), 60.7 (C-3′), 55.6 (C3p+5p), 53.8 (C-7″), 50.3 (C2p+6p), 18.9 (C-9); TOF MS ES+: [M + H]^+^ calcd. for C_24_H_28_O_5_N_2_: 425.2071 found 425.2083.

##### 7-(2-Hydroxy-3-(4-(3-methoxyphenyl)piperazin-1-yl)propoxy)-4-methyl-2*H*-chromen-2-one (**10**)

m.p.: 128 °C, Rf = 0.77, yield 56%, ^1^H NMR (300 MHz, CDCl3) δ ppm: 7.50 (d, *J* = 5.4 Hz, 1H, H-5), 7.18 (t, *J* = 4.9 Hz, 1H, H-5″), 6.92 (d, *J* = 6.9 Hz, 1H, H-8), 6.84 (d, 1.5 Hz, 1H, H-6), 6.55 (d, *J* = 1.8 Hz, 1H, H-6″), 6.47 (t, *J* = 1.3 Hz, 1H, H-4″), 6.43 (d, *J* = 6.6 Hz, 1H, H-2″), 6.14 (s, 1H, H-3), 4.20–4.15 (m, 1H, H-2′), 4.10–4.03 (m, 2H, H-1′), 3.79 (s, 3H, H-7″), 3.27–3.19 (m, 4H, H3p+5p), 2.87–2.83 (m, 2H, H-3′), 2.67–2.57 (m, 4H, H2p+6p), 2.40 (s, 3H, H-9); ^13^C NMR (300 MHz, CDCl_3_, δ ppm): 161.6 (C-3″), 161.2 (C-2), 160.5 (C-7), 155.1 (C-8a), 152.4 (C-4), 152.4 (C-1″), 129.8 (C-5″), 125.5 (C-5), 113.8 (C-3), 112.5 (C-4a), 112.1 (C-6), 108.9 (C-6″), 104.6 (C-8), 102.6 (C-2″), 101.6 (C-4″), 70.6 (C-1′), 65.3 (C-2′), 60.2 (C-3′), 55.1 (C3p+5p), 53.2 (C-7″), 59.1 (C2p+6p), 18.9 (C-9); TOF MS ES+: [M + H]^+^ calcd. for C_24_H_28_O_5_N_2_: 425.2071 found 425.2075.

##### 8-Acetyl-7-(2-hydroxy-3-(4-(2-methoxyphenyl)piperazin-1-yl)propoxy)-4-methyl-2*H*-chromen-2-one (**11**)

m.p.: 171 °C, Rf = 0.63, yield 69%, ^1^H NMR (300 MHz, CDCl3) δ ppm: 7.56 (d, *J* = 5.4 Hz, 1H, H-5), 7.03–6.99 (m, 1H, H-3″), 6.99–6.92 (m, 3H, H-4″, H-5″, H-6″), 6.87 (d, 5.1 Hz, 1H, H-6), 6.16 (s, 1H, H-3), 4.18–4.16 (m, 1H, H-2′), 4.13–4.10 (m, 2H, H-1′), 3.87 (s, 3H, H-7″), 3.10 (br. s., 4H, H3p+5p), 2.89–2.85 (m, 2H, H-3′), 2.68–2.55 (m, 7H, H2p+6p, H-11), 2.40 (s, 3H, H-9); ^13^C NMR (300 MHz, CDCl_3_, δ ppm): 199.2 (C-10), 159.8 (C-1″), 157.8 (C-2), 152.2 (C-7), 151.9 (C-8a), 150.7 (C-4), 141.0 (C-2″), 126.4 (C-5), 123.0 (C-6″), 120.9 (C-4″), 119.8 (C-5″), 118.1 (C-8), 114.2 (C-6), 112.8 (C-3″), 111.2 (C-3), 109.0 (C-4a), 71.6 (C-1′), 65.4 (C-2′), 60.1 (C-3′), 55.3 (C3p+5p), 55.5 (C-7″), 50.6 (C2p+6p), 32.5 (C-11), 18.7 (C-9); TOF MS ES+: [M + H]^+^ calcd. for C_26_H_30_O_6_N_2_: 467.2176 found 467.2182.

##### 8-Acetyl-7-(2-hydroxy-3-(4-(3-methoxyphenyl)piperazin-1-yl)propoxy)-4-methyl-2*H*-chromen-2-one (**12**)

Oil, Rf = 0.68, yield 58%, ^1^H NMR (300 MHz, CDCl3) δ ppm: 7.57 (d, *J* = 6.9 Hz, 1H, H-5), 7.18 (t, *J* = 4.9 Hz, 1H, H-5″), 6.94 (d, *J* = 5.4 Hz, 1H, H-6), 6.55–6.53 (m, 1H, H-6″), 6.44–6.42 (m, 2H, H-2″, H-4″), 6.16 (s, 1H, H-3), 4.18–4.11 (m, 3H, H-1′, H-2′), 3.79 (s, 3H, H-7″), 3.22–3.19 (m, 4H, H3p+5p), 2.83–2.78 (m, 2H, H-3′), 2.62–2.60 (m, 7H, H2p+6p, H-11), 2.40 (s, 3H, H-9); ^13^C NMR (300 MHz, CDCl_3_, δ ppm): 199.2 (C-10), 160.6 (C-3″), 159.7 (C-2), 157.8 (C-7), 152.4 (C-8a), 151.9 (C-4), 150.8 (C-1″), 129.7 (C-5″), 126.4 (C-5), 119.7 (C-8), 114.2 (C-6), 112.8 (C-3), 109.0 (C-4a), 108.8 (C-4″), 104.5 (C-6″), 102.5 (C-2″), 71.6 (C-1′), 65.5 (C-2′), 60.1 (C-3′), 55.1 (C3p+5p), 53.2 (C-7″), 49.1 (C2p+6p), 32.5 (C-11), 18.7 (C-9); TOF MS ES+: [M + H]^+^ calcd. for C_26_H_30_O_6_N_2_: 467.2176 found 467.2184.

##### 7-(2-Hydroxy-3-(4-(2-methoxyphenyl)piperazin-1-yl)propoxy)-2*H*-chromen-2-one (**13**)

Oil, Rf = 0.82, yield 95%, ^1^H NMR (300 MHz, CDCl3) δ ppm: 7.63 (d, *J* = 9.3 Hz, 1H, H-4), 7.37 (d, *J* = 8.4 Hz, 1H, H-5), 7.04–7.00 (m, 1H, H-8), 6.99–6.85 (m, 5H, H-6, H-3″-H-6″), 6.26 (d, *J* = 9.6 Hz, 1H, H-3), 4.21–4.14 (m, 1H, H-2′), 4.12–4.00 (m, 2H, H-1′), 3.87 (s, 3H, H-7″), 3.12 (br. s., 4H, H3p+5p), 2.94–2.88 (m, 2H, H-3′), 2.72–2.57 (m, 4H, H2p+6p); ^13^C NMR (300 MHz, CDCl_3_, δ ppm): 161.9 (C-1″), 161.1 (C-2), 155.7 (C-7), 152.2 (C-8a), 143.3 (C-4), 141.0 (C-2″), 128.7 (C-5), 123.1 (C-6″), 120.9 (C-4″), 118.2 (C-5″), 113.2 (C-3), 112.9 (C-4a), 112.7 (C-6), 111.8 (C-3″), 101.5 (C-8), 71.7 (C-1′), 65.1 (C-2′), 60.2 (C-3′), 55.3 (C3p+5p), 53.4 (C-7″), 50.6 (C2p+6p); TOF MS ES+: [M + H]^+^ calcd. for C_23_H_26_O_5_N_2_: 433.1733 found 433.1732.

##### 7-(2-Hydroxy-3-(4-(3-methoxyphenyl)piperazin-1-yl)propoxy)-2*H*-chromen-2-one (**14**)

m.p.: 112–114 °C, Rf = 0.73, yield 70%, ^1^H NMR (300 MHz, CDCl3) δ ppm: 7.65 (d, *J* = 9.3 Hz, 1H, H-4), 7.37 (d, *J* = 8.4 Hz, 1H, H-5), 7.18 (t, *J* = 8.1 Hz, 1H, H-5″), 6.91–6.84 (m, 2H, H-6, H-8), 6.54 (d, *J* = 10.2 Hz, 1H, H-6″), 6.47–6.42 (m, 2H, H-2″, H-4″), 6.25 (d, *J* = 9.6 Hz, 1H, H-3), 4.23–4.16 (m, 1H, H-2′), 4.11–4.02 (m, 2H, H-1′), 3.79 (s, 3H, H-7″), 3.29–3.19 (m, 4H, H3p+5p), 2.92–2.85 (m, 2H, H-3′), 2.73–2.59 (m, 4H, H2p+6p); ^13^C NMR (300 MHz, CDCl_3_, δ ppm): 161.8 (C-3″), 161.1 (C-2), 160.5 (C-7), 155.7 (C-8a), 152.4 (C-1″), 129.8 (C-5″), 128.8 (C-5), 113.3 (C-3), 112.8 (C-4a), 112.8 (C-6), 108.9 (C-4″), 104.7 (C-6″), 102.6 (C-8), 101.6 (C-2″), 70.6 (C-1′), 65.2 (C-2′), 60.2 (C-3′), 55.2 (C3p+5p), 53.2 (C-7″), 49.0 (C2p+6p); TOF MS ES+: [M + H]^+^ calcd. for C_23_H_26_O_5_N_2_: 433.1914 found 433.1916.

#### 3.1.3. Biological Assays

##### Membrane Preparation

Sprague–Dawley rats were decapitated under isoflurane anesthesia. Brains were rapidly removed and placed on ice. Hippocampi (for the 5-HT_1A_ assay) or frontal cortices (for the 5-HT_2A_ assay) were dissected on a Petri dish. The tissue from 10 rats was homogenized in 30 vol. homogenization buffer (50 mM Tris-HCl, pH = 4.7, 1 mM EDTA, 1mM dithiothreitol) with a hand held Teflon-glass homogenizer. The homogenate was centrifuged at 48,000× *g* at 4 °C for 15 min. The pellet was suspended and homogenized in homogenization buffer and incubated for 10 min at 36 °C. The centrifugation and suspension steps were repeated twice. The final pellet was homogenized in 5 vol. 50 mM Tris-HCl, pH = 7.4 buffer and stored at −80 °C for no longer than 6 months.

##### Competitive Binding Assays

For the 5-HT_1A_ assay, seven concentrations of the compounds tested equally spaced on a log scale (10^−10^ M–10^−4^ M) were incubated for 60 min. at 36 °C in binding buffer (50 mM Tris-HCl, pH 7.4, 0.1% ascorbate, 5 mM MgCl_2_) with 0.4 nM [^3^H]8-OH-DPAT (specific activity: 200 Ci/mmol, Revvity, Waltham, MA, USA) and 80 µg of the hippocampal membranes. For the 5-HT_2A_ receptor assay, 160 µg/mL of the frontal cortex membranes was incubated with 1 nM [^3^H]ketanserin in binding buffer (50 mM Tris-HCl, 0.1% ascorbate, 3 mM CaCl_2,_ 120 mM NaCl, 5 mM KCl). Non-specific binding was determined with 10 μM serotonin. The final DMSO concentration in the assay was 5%. After incubation, the reaction mixture was deposited with the FilterMate-96 Harvester (Revvity, Waltham, MA, USA) onto Unifilter^®^ GF/C plates (Revvity, Waltham, MA, USA) presoaked in 0.4% PEI for 1 h. Each well was washed with 2 mL of 50 mM Tris-HCl (pH 7.4) buffer to separate bound ligands from free. Plates were left to dry overnight. Then, 35 µL of Microscint-20 scintillation fluid (Revvity, Waltham, MA, USA) was added to each filter well and left to equilibrate for 2 h. Filter-bound radioactivity was counted in a MicroBeta^2^ LumiJet scintillation counter (Revvity, Waltham, MA, USA). Binding curves were fitted with one site non-linear regression. The binding affinity (Ki) for each compound was calculated from the EC_50_ values with the Cheng–Prusoff equation from two separate experiments.

##### Antagonist Activity at 5-HT1A Receptors

Serial dilutions of the compounds tested (10^−10^–10^−4^ M) were incubated in duplicate with 0.8 nM [^35^S]GTPγS in assay buffer (50 mM Tris-HCl, pH = 7.4, 1 mM EGTA, 3 mM MgCl_2_, 100 mM NaCl, 30 µM GDP) and 1 µM 8-OH-DPAT. Then, hippocampal membrane homogenates (15 μg/mL) were added to each well, and the mixture was incubated for 90 min. at 37 °C in a total volume of 250 µL. Non-specific binding was determined with 10 µM of unlabeled GTPγS. The reaction was terminated by vacuum filtration onto GF/C Unifilter Plates (Revvity, Waltham, MA, USA) presoaked with 50 mM Tris-HCl (pH = 7.4) for 1 h with the FilterMate Harvester^®^ (Revvity, Waltham, MA, USA). The samples were then rapidly washed with 2 mL of 50 mM Tris-HCl (pH = 7.4) buffer. The Unifilter plates were dried overnight at RT. After drying, 35 µL of EcoScint-20 scintillant (Revvity, Waltham, MA, USA) was added to each well. Radioactivity was counted in a Trilux MicroBeta^2^ counter (Revvity, Waltham, MA, USA). Data were analyzed with GraphPad Prism 5.0 software (GraphPad Software, San Diego, CA, USA, accessed on Sept 12, 2022, www.graphpad.com). The curves were fitted with a one-site non-linear regression model to determine inhibitory potency (IC_50_). The experiments were repeated twice.

#### 3.1.4. Theoretical Methodology

In the computational part of this study, we used a protocol similar to our previous investigations on this topic [28,29,30,31,32,33], but based on recently obtained crystal structures of 5-HT_1A_ and 5-HT_2A_ receptors, as in our latest study on this topic [34]. For the 5-HT_1A_ receptor, we selected three crystal structures: apo-5-HT_1A_ (PDB id: 7e2x), serotonin-bound 5-HT_1A_ (PDB id: 7e2y) and aripiprazole-bound 5-HT_1A_ (PDB id: 7e2z), which were all complexed to a G protein [42]. In the case of the 5-HT_2A_ receptor, we selected two crystal structures: 5-HT_2A_ in complex with serotonin (PDB id: 7wc4) and 5-HT_2A_ in complex with aripiprazole (PDB id: 7voe) [43,44]. We selected these particular structures on the basis of a high similarity of compounds studied in this work to aripiprazole. Next, we performed standard flexible docking for each ligand–receptor pair for each of the five GPCR crystal structures using Autodock Vina ver. 1.1.2 with the exhaustiveness parameter set to 18. In the case of the 5-HT_1A_ receptor, the following residues were described in a flexible manner: Y96, Q97, F112, D116, T121, S199, F361, N386, and Y390, while for the 5-HT_2A_ receptor, the flexible residues were W151, D155, V156, F243, F332, W336, F339, F340, N363, and V366. Values presented in Table 3 are the lowest Gibbs free energy values from all docking experiments found for each of the studied ligands, which displayed the crucial interaction between the basic nitrogen atom of piperazine and D116 (for the 5HT_1A_ receptor) or D155 (for the 5HT_2A_ receptor). Additionally, we performed a computational assessment of ADME properties using QikProp 4.6 software and evaluated the pKa values of basic nitrogen-containing functional groups using Epik 5.3 software [45]. Toxicity has been estimated using the ProTox 3.0 server [46].

## 4. Conclusions

In summary, the outcome of this study was the synthesis of new coumarin derivatives and the determination of their binding profile and receptor functionality for 5-HT_1A_ receptors as well as their affinity for 5-HT_2A_ receptors. Our results demonstrated that both the type of substituents and their positioning on the phenyl ring, the coumarin-derived portion, and the carbon linker significantly influence the affinity and activity profile of the compounds toward these receptors.

The compound with an acetyl group at position 8 of the coumarin ring and a methoxy substituent in the ortho position of the phenyl ring (**11**) exhibited the strongest antagonistic activity toward the 5-HT_1A_ receptor. Shifting the methoxy group to the ortho position resulted in nearly a 20-fold increase in affinity for the 5-HT_1A_ receptor (compound **11** vs. **12**). The weakest affinity for the 5-HT_1A_ receptor was observed in the compound with a methoxy substituent in the meta position, a coumarin core substituted with two methyl groups at positions C-4 and C-7, and an acetyl group at position 6 (compound **8**). Adding an acetyl group at position C-6 of the 2H-chromen-2-one ring caused a more than 10-fold decrease in antagonistic activity toward the 5-HT1A receptor (compound **6** vs. **8**). The attachment of an acetyl group at position 8 of the heterocyclic coumarin derivative enhanced its antagonistic activity toward the 5-HT_1A_ receptor (compounds **9** vs. **11** and **10** vs. **12**). The strongest affinity for the 5-HT_2A_ receptor was found for compounds with an unsubstituted 2H-chromen-2-one ring or a ring substituted with dimethyl groups at positions C-4 and C-7 along with an acetyl group at position 6 or 8 (compounds **13**, **14**, **12** and **2**). Based on all data, both experimental and computational, we identified compounds **11** (8-acetyl-7-(2-hydroxy-3-(4-(2-methoxyphenyl)piperazin-1-yl)propoxy)-4-methyl-2*H*-chromen-2-one) and **7** (6-acetyl-5-(2-hydroxy-3-(4-(2-methoxy phenyl)piperazin-1-yl)propoxy)-4,7-dimethyl-2*H*-chromen-2-one) as lead structures with the most favorable receptor profile and aligning with the current concept of designing multifunctional antidepressant drugs.

## Figures and Tables

**Table 1:** Binding affinities for 5-HT1A and 5-HT2A receptors and antagonist activity at the 5-HT1A receptors for compounds **1**–**14**.

Compound	5-HT_1A_ Ki (nM, 95% CI)	5-HT_1A_ IC_50_ (µM, 95% CI)	5-HT_2A_ Ki (nM, 95% CI)
**1**	243 (136–437)	331 (55–1985)	1846 (1126–10,440)
**2**	196 (134–288)	59 (18–160)	83 (30–229)
**3**	243 (159–372)	68 (37–129)	4006 (1832–8761)
**4**	190 (118–307)	10 (0.49–23)	315 (131–760)
**5**	90 (54–150)	14.7 (7.1–30)	1182 (516–2806)
**6**	176 (97–319)	6.5 (2.5–17.3)	662 (320–1373)
**7**	90 (56–147)	2.4 (0.58–9.8)	4150 (2276–7568)
**8**	1776 (354–8916)	16.2 (6.2–42.7)	115 (36–367)
**9**	245 (143–419)	2.4 (0.7–7.9)	291 (134–629)
**10**	87 (53–142)	89.5 (30.2–265)	260 (111–607)
**11**	96 (50–183)	0.043 (0.014–0.137)	6157 (3184–11,910)
**12**	264 (162–432)	6.1 (1.76–21.5)	67 (26–169)
**13**	222 (124–396)	1.43 (0.63–32.6)	18 (6–61)
**14**	135 (85–216)	112 (48.9–256.3)	68 (41–113)
**8-OH-DPAT**	0.68 (0.45–1.02)	-	-
**Ketanserin**	-	-	0.56 (0.23–1.36)
**WAY-100635**		0.0043 (0.00186–0.0075)	

**Table 2:** Predicted ADMET properties for compounds **1**–**14**.

Compound	MW ^a^	Dipole ^b^	vol ^c^	SASA ^d^	dHB ^e^	aHB ^f^	logP ^g^	metab ^h^	Ro3 ^i^	Ro5 ^j^	pKa ^k^	LD_50_ ^l^
**1**	466.5	7.1	777.6	1426.2	1	10.7	2.36	6	0	0	8.97	500
**2**	466.5	3.7	764.7	1418.1	1	10.7	2.30	5	0	0	8.97	550
**3**	410.5	3.5	705.7	1270.5	1	8.7	2.77	5	0	0	9.05	1100
**4**	410.5	4.5	705.1	1273.8	1	8.7	2.78	5	0	0	9.05	550
**5**	438.5	6.7	753.1	1367.3	1	8.7	3.38	7	0	1	8.99	1100
**6**	438.5	7.6	761.9	1387.3	1	8.7	3.37	6	0	0	9.00	676
**7**	480.6	6.1	774.1	1433.4	1	10.7	2.76	7	0	1	8.99	500
**8**	480.6	8.7	763.9	1425.7	1	10.7	2.70	7	0	1	8.99	550
**9**	424.5	9.0	758.5	1356.8	1	8.7	3.17	6	0	0	8.97	1100
**10**	424.5	6.5	745.6	1348.7	1	8.7	3.11	5	0	0	8.97	1100
**11**	466.5	12.7	775.3	1420.3	1	10.7	2.52	6	0	0	8.97	550
**12**	466.5	10.0	762.4	1412.2	1	10.7	2.46	5	0	0	8.97	1000
**13**	410.5	8.7	733.7	1305.2	1	8.7	2.91	5	0	0	8.97	1100
**14**	410.5	6.1	720.7	1297.1	1	8.7	2.85	4	0	0	8.97	550
**aripiprazole**	448.4	7.8	709	1318.3	1	6.25	4.43	5	0	0	7.39	800
**ketanserin**	395.4	7.8	699.4	1235.5	1	7.5	2.93	3	0	0	6.45	790
**8-OH-DPAT**	247.4	1	556.1	959.5	1	2.75	3.46	4	0	0	9.4	150
**WAY100635**	422.6	3.9	761.8	1413.2	0	8.25	3.95	6	0	0	6.34	874

^a^ MW–molecular weight (Da); ^b^ dipole—dipole moment (D); ^c^ vol—total molecular volume (Å^3^); ^d^ SASA—solvent accessible surface (Å^2^); ^e^ dHB—estimated number of hydrogen bonds that would be donated by the solute to water molecules in an aqueous solution; ^f^ aHB—estimated number of hydrogen bonds that would be accepted by the solute from water molecules in an aqueous solution; ^g^ logP—octanol/water partition coefficient; ^h^ metab—number of likely metabolic reactions; ^i^ Ro3—number of violations of Jorgensen’s rule of three; ^j^ Ro5—number of violations of Lipinski’s rule of five; ^k^ pKa—predicted pKa value of the basic nitrogen atom; ^l^ LD_50_—predicted value of lethal dose (mg/kg).

**Table 3:** Predicted Gibbs free energies of binding (ΔG) and Ki of compounds **1**–**14** to 5-HT receptors.

	5HT_1A_		5HT_2A_	
Compound	ΔG [kcal/mol]	Ki [nM]	ΔG [kcal/mol]	Ki [nM]
**1**	<−9.0	>250	−10.8	12.1
**2**	−9.8	65.5	−10.3	28.2
**3**	<−9.0	>250	−11.0	8.6
**4**	−9.6	91.9	−10.7	14.3
**5**	−9.4	128.7	−10.7	14.3
**6**	<−9.0	>250	−10.5	20.1
**7**	−10.0	46.8	−11.3	5.2
**8**	<−9.0	>250	−10.9	10.2
**9**	<−9.0	>250	−9.8	65.5
**10**	−9.2	180.4	−10.0	46.8
**11**	−9.4	128.7	−10.9	10.2
**12**	−9.2	180.4	−10.3	28.2
**13**	<−9.0	>250	−9.7	77.6
**14**	−9.5	108.8	−9.8	65.5
**aripiprazole**	−9.5	108.8	−9.9	55.4
**ketanserin**	−9.6	-	−11.7	2.7

## Data Availability

No new data were created or analyzed in this study. Data sharing is not applicable to this article.

## Supplementary Materials

The following supporting information can be downloaded at https://www.mdpi.com/article/10.3390/ijms26051946/s1.
