# Supplementary material for: Exploring the Potential of Coumarin Derivatives on Serotonin Receptors 5-HT1A and 5HT2A
Source: Int J Mol Sci. 2025 Feb 24;26(5):1946. doi: 10.3390/ijms26051946 (PMC11900313; doi:10.3390/ijms26051946)

Exploring the potential of coumarin derivatives on serotonin receptors 5-HT_{1A} and $5HT_{2A}$

Kinga Ostrowska[1,*], Gabriela Horosz[1], Karolina Kruk[1], Bartłomiej Sieroń[1], Anna Leśniak[2], Zofia Czartoryska[2], Magdalena Bujalska-Zadrożny[2], Dejan Milenkovic[3], Bartosz Trzaskowski[4]

*Correspondence author: kostrowska@wum.edu.pl

[1]Department of Organic and Physical Chemistry, Faculty of Pharmacy, Medical University of Warsaw, Banacha 1, 02-097 Warsaw, Poland

[2]Faculty of Pharmacy, Department of Pharmacotherapy and Pharmaceutical Care, Medical University of Warsaw, Banacha 1, 02-097, Warsaw, Poland

[3]Department of Science, Institute for Information Technologies, University of Kragujevac, Jovana Cvijića bb, 34000 Kragujevac, Serbia

[4]Centre of New Technologies, University of Warsaw, 2C Banacha Str., 02-097 Warsaw, Poland

Figure S1: ^{1}H and ^{13}C NMR spectra

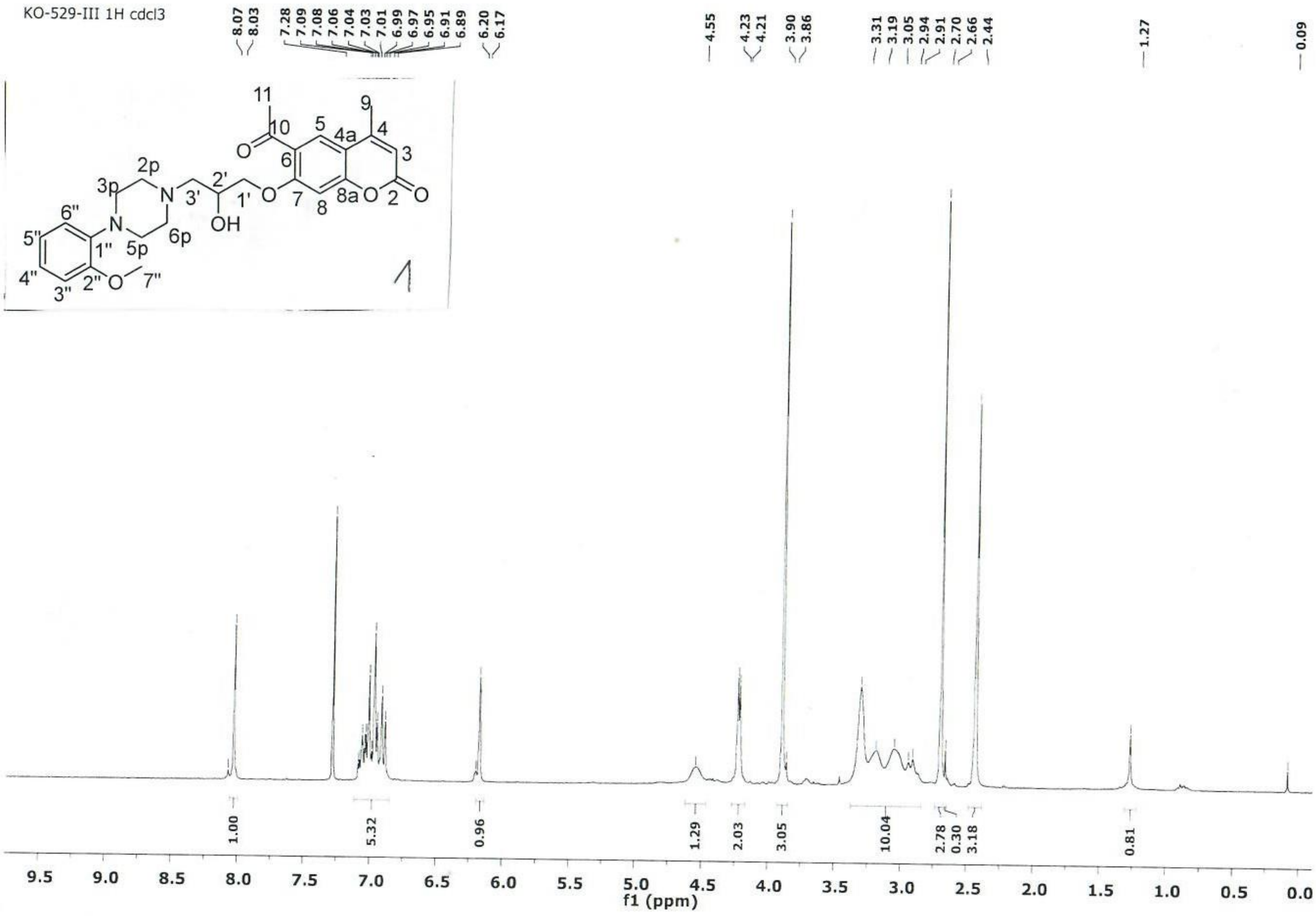
KO-529-III 1H cdcl3
8.07
8.03
7.28
7.09
7.08
7.06
7.04
7.03
7.01
6.99
6.97
6.95
6.91
6.89
6.20
6.17
4.55
4.23
4.21
3.90
3.86
3.31
3.19
3.05
2.94
2.91
2.70
2.66
2.44
1.27
0.09
1.00
5.32
0.96
1.29
2.03
3.05
10.04
2.78
0.30
3.18
0.81
9.5
9.0
8.5
8.0
7.5
7.0
6.5
6.0
5.5
5.0
4.5
4.0
3.5
3.0
2.5
2.0
1.5
1.0
0.5
0.0
f1 (ppm)

1

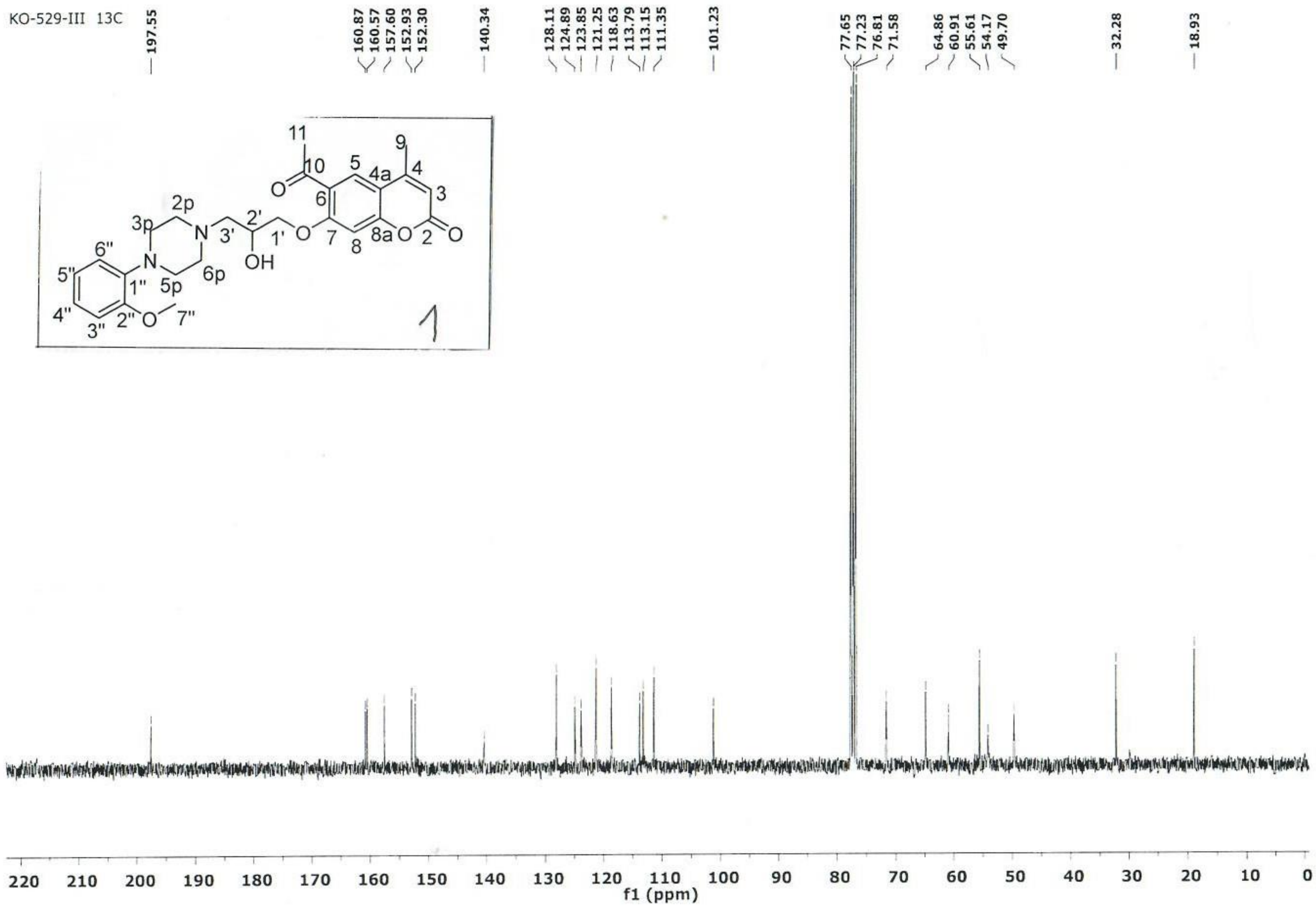

KO-529-III 13C
197.55
160.87
160.57
157.60
152.93
152.30
140.34
128.11
124.89
123.85
121.25
118.63
113.79
113.15
111.35
101.23
77.65
77.23
76.81
71.58
64.86
60.91
55.61
54.17
49.70
32.28
18.93
11
9
10
5
4a
4
3
6
2p
2'
3p
N
3'
1'
O
7
8a
O
2
O
6''
N
OH
8
5''
1''
5p
6p
4''
3''
2''
O
7''
1
220 210 200 190 180 170 160 150 140 130 120 110 100 90 80 70 60 50 40 30 20 10 0
f1 (ppm)

9181-1H
538 I

2

Parameter	Value
1 Title	
2 Owner	felix
3 Solvent	CDCl3
4 Temperature	298.2
5 Pulse Sequence	zg30
6 Experiment	1D
7 Number of Scans	8
8 Receiver Gain	29.7
9 Relaxation Delay	0.0000
10 Pulse Width	11.3000
11 Acquisition Time	3.2768
12 Acquisition Date	2023-10-25T17:05:16
13 Spectrometer Frequency	500.20
14 Spectral Width	10000.0
15 Lowest Frequency	-1922.3
16 Nucleus	1H
17 Acquired Size	32768
18 Spectral Size	65536

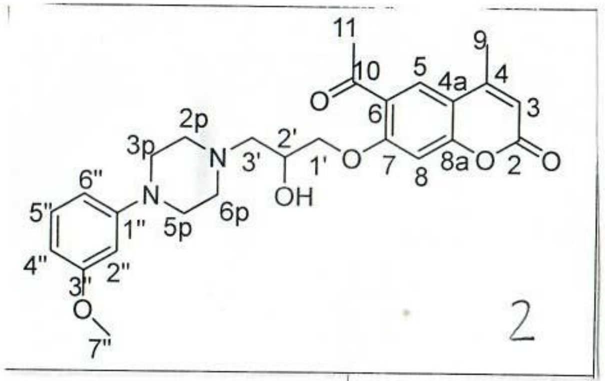

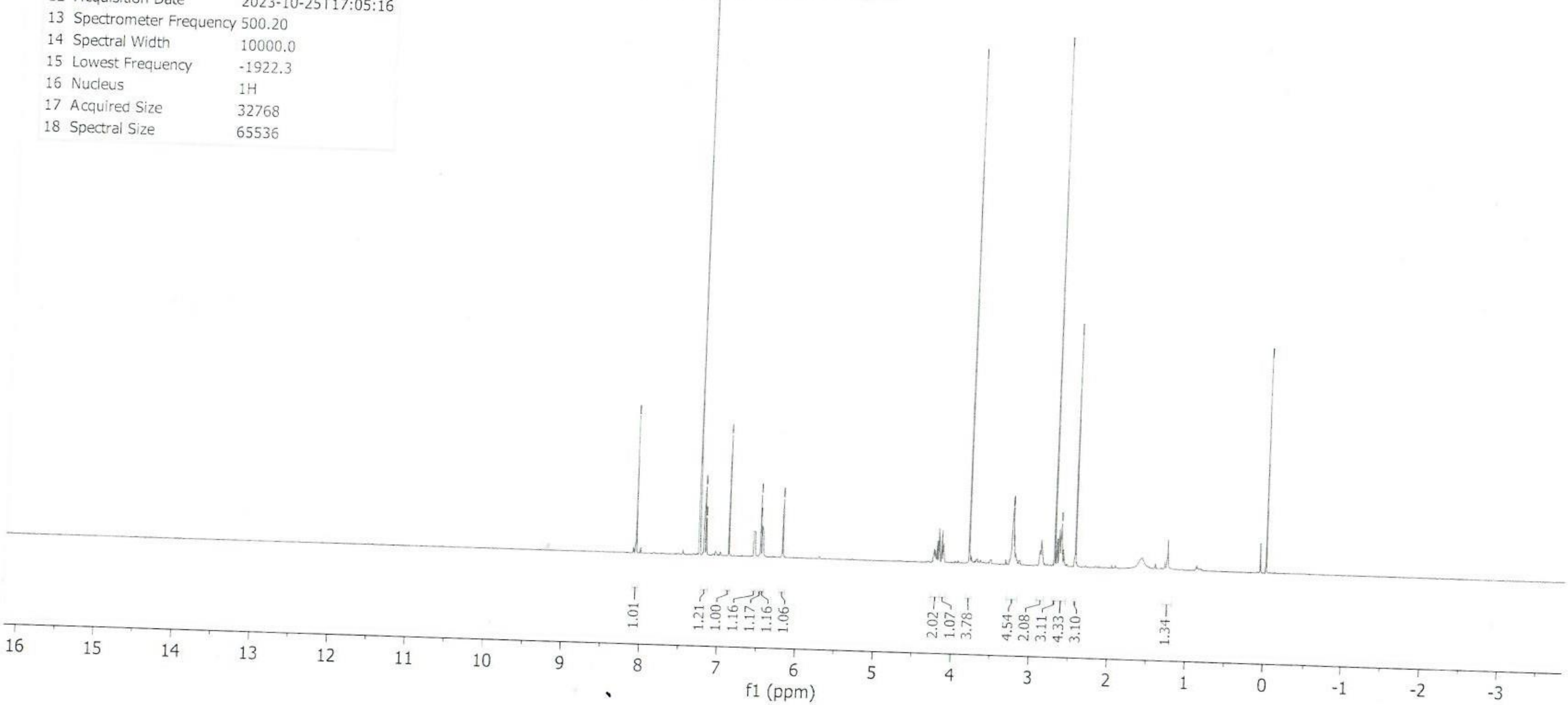

2

Parameter	Value
Comment	51090-13C<br>538 I
Origin	Bruker BioSpin GmbH
Solvent	CDCl3
Temperature	298.1
Pulse Sequence	zgpg30
Number of Scans	2300
Receiver Gain	200
Relaxation Delay	1.5000
Pulse Width	10.0000
Acquisition Time	1.7302
Acquisition Date	2023-11-27T21:50:17
Spectrometer Frequency	75.49
Spectral Width	18939.4
Lowest Frequency	-790.0
Nucleus	13C
Acquired Size	32768
Spectral Size	65536

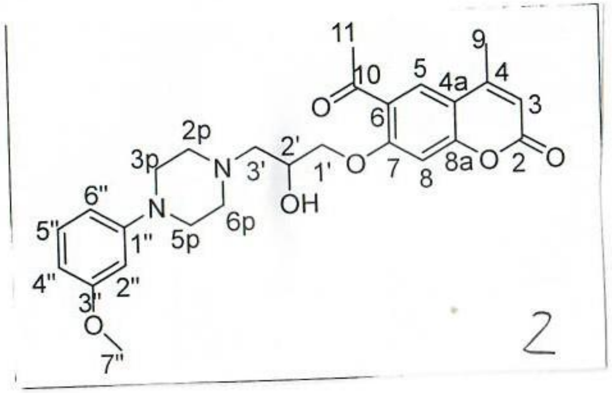

2

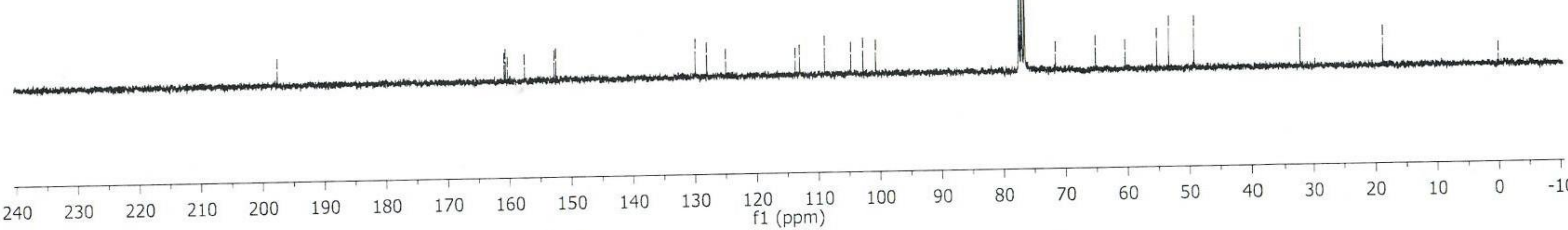

3

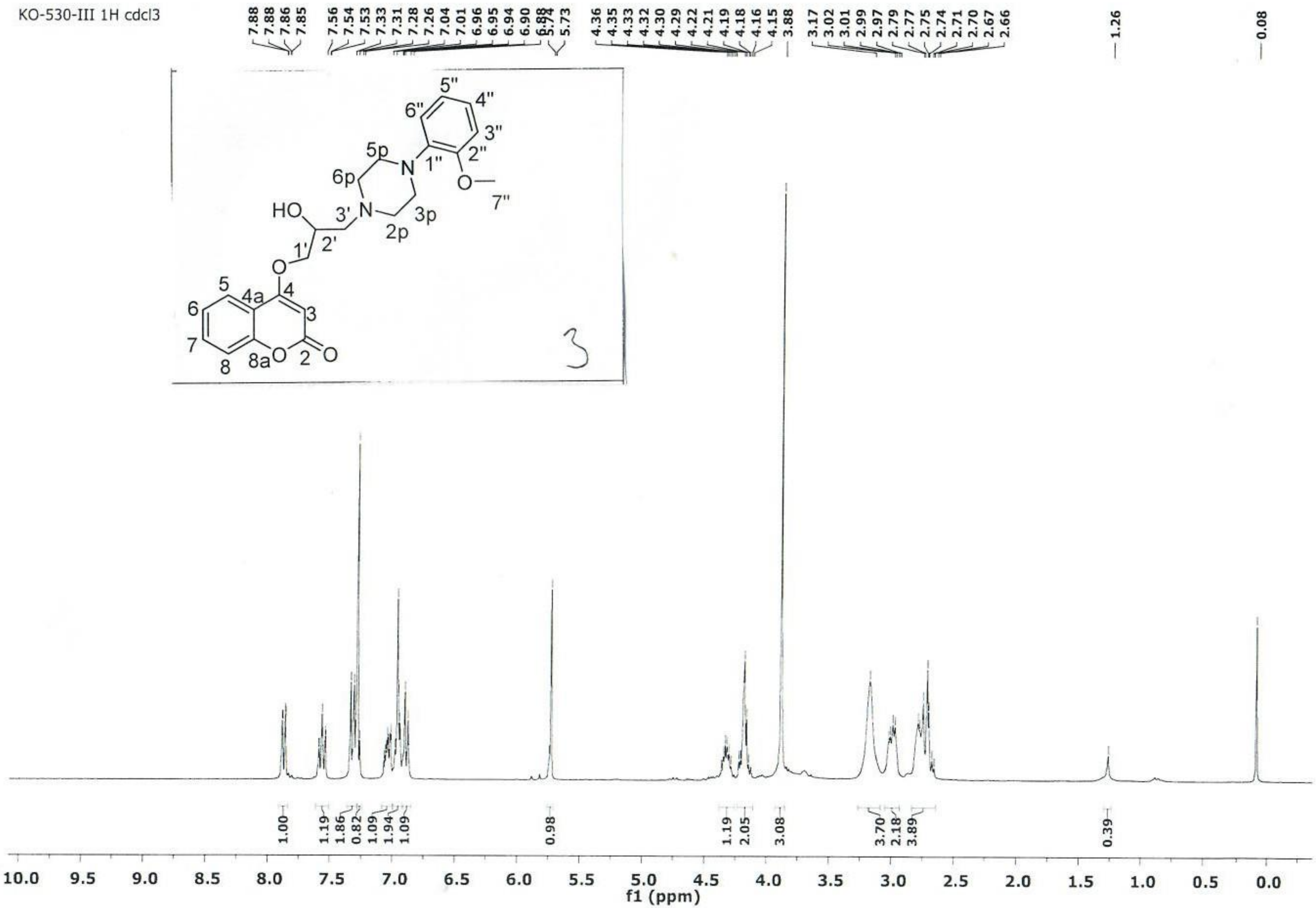

KO-530-III 1H cdcl3
7.88 7.88 7.86 7.85 7.56 7.54 7.53 7.33 7.31 7.28 7.26 7.04 7.01 6.96 6.95 6.94 6.90 6.88 5.74 5.73 4.36 4.35 4.33 4.32 4.30 4.29 4.22 4.21 4.19 4.18 4.16 4.15 3.88 3.17 3.02 3.01 2.99 2.97 2.79 2.77 2.75 2.74 2.71 2.70 2.67 2.66 1.26 0.08
1.00 1.19 1.86 0.82 1.09 1.94 1.09 0.98 1.19 2.05 3.08 3.70 2.18 3.89 0.39
10.0 9.5 9.0 8.5 8.0 7.5 7.0 6.5 6.0 5.5 5.0 4.5 4.0 3.5 3.0 2.5 2.0 1.5 1.0 0.5 0.0
f1 (ppm)
5" 4" 6" 3" 5p 2" 1" 6p N O 7" HO 3' N 3p 2p 1' 2' O 5 4a 4 6 3 7 8 8a O 2 O
3

3

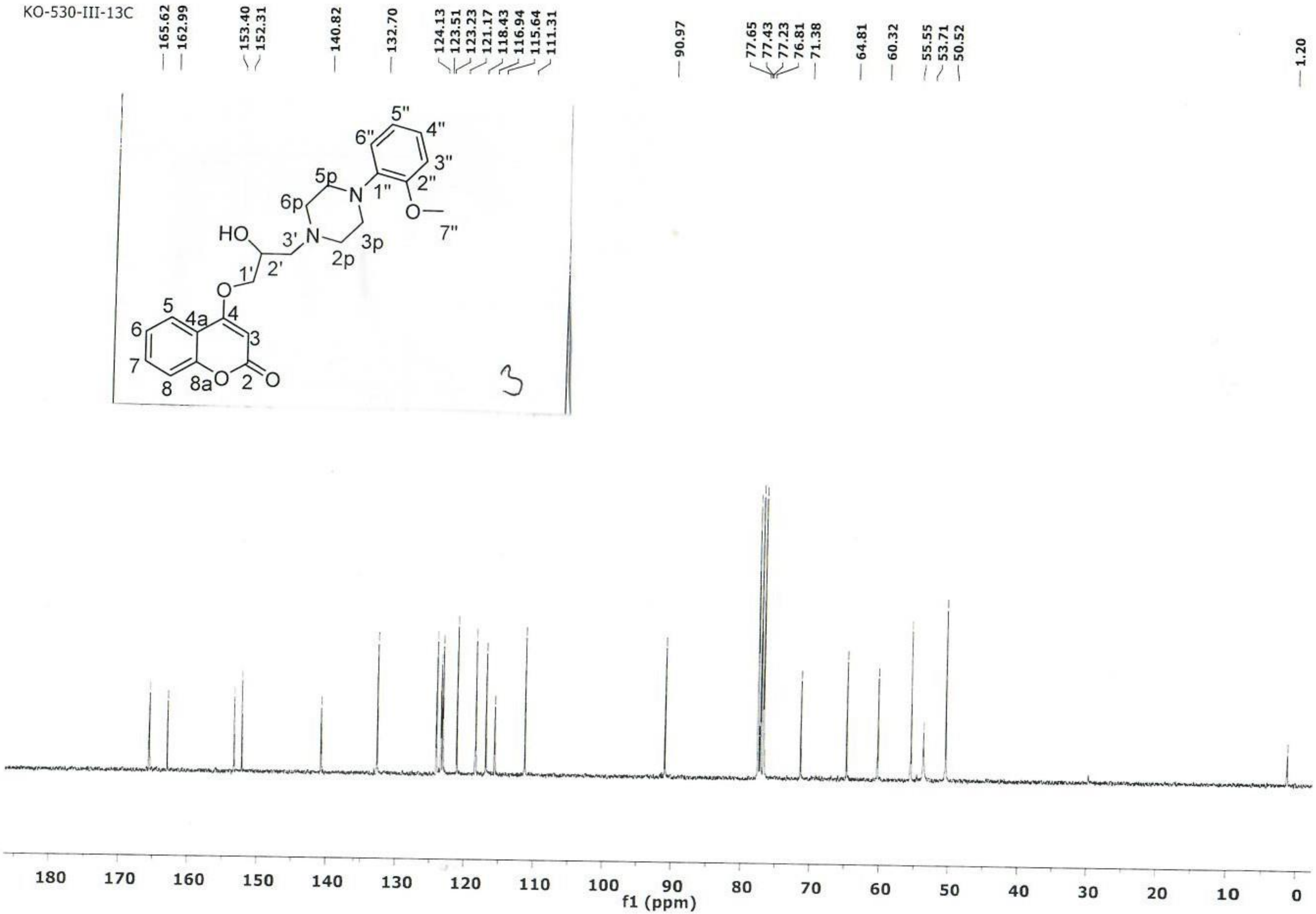
KO-530-III-13C
165.62
162.99
153.40
152.31
140.82
132.70
124.13
123.51
123.23
121.17
118.43
116.94
115.64
111.31
90.97
77.65
77.43
77.23
76.81
71.38
64.81
60.32
55.55
53.71
50.52
1.20
5"
4"
6"
3"
5p
N
1"
2"
6p
O
HO
3'
N
3p
7"
1'
2'
2p
O
5
4a
4
6
3
7
8
8a
O
2
O
3
180 170 160 150 140 130 120 110 100 90 80 70 60 50 40 30 20 10 0
f1 (ppm)

9179-1H
537 I

Parameters		
	Parameter	Value
1	Title	
2	Owner	felix
3	Solvent	CDCl3
4	Temperature	298.1
5	Pulse Sequence	zg30
6	Experiment	1D
7	Number of Scans	8
8	Receiver Gain	29.7
9	Relaxation Delay	0.0000
10	Pulse Width	11.3000
11	Acquisition Time	3.2768
12	Acquisition Date	2023-10-25T17:00:51
13	Spectrometer Frequency	500.20
14	Spectral Width	10000.0
15	Lowest Frequency	-1921.7
16	Nucleus	1H
17	Acquired Size	32768
18	Spectral Size	65536

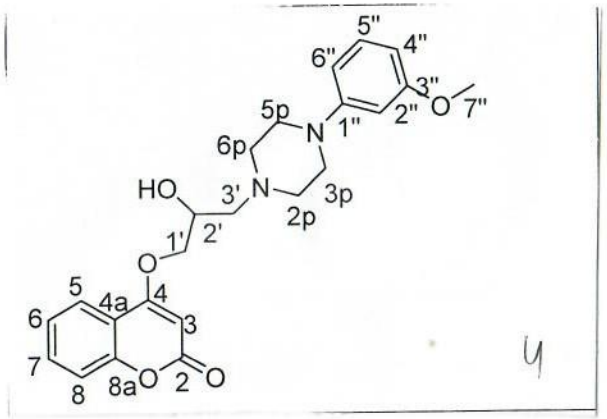

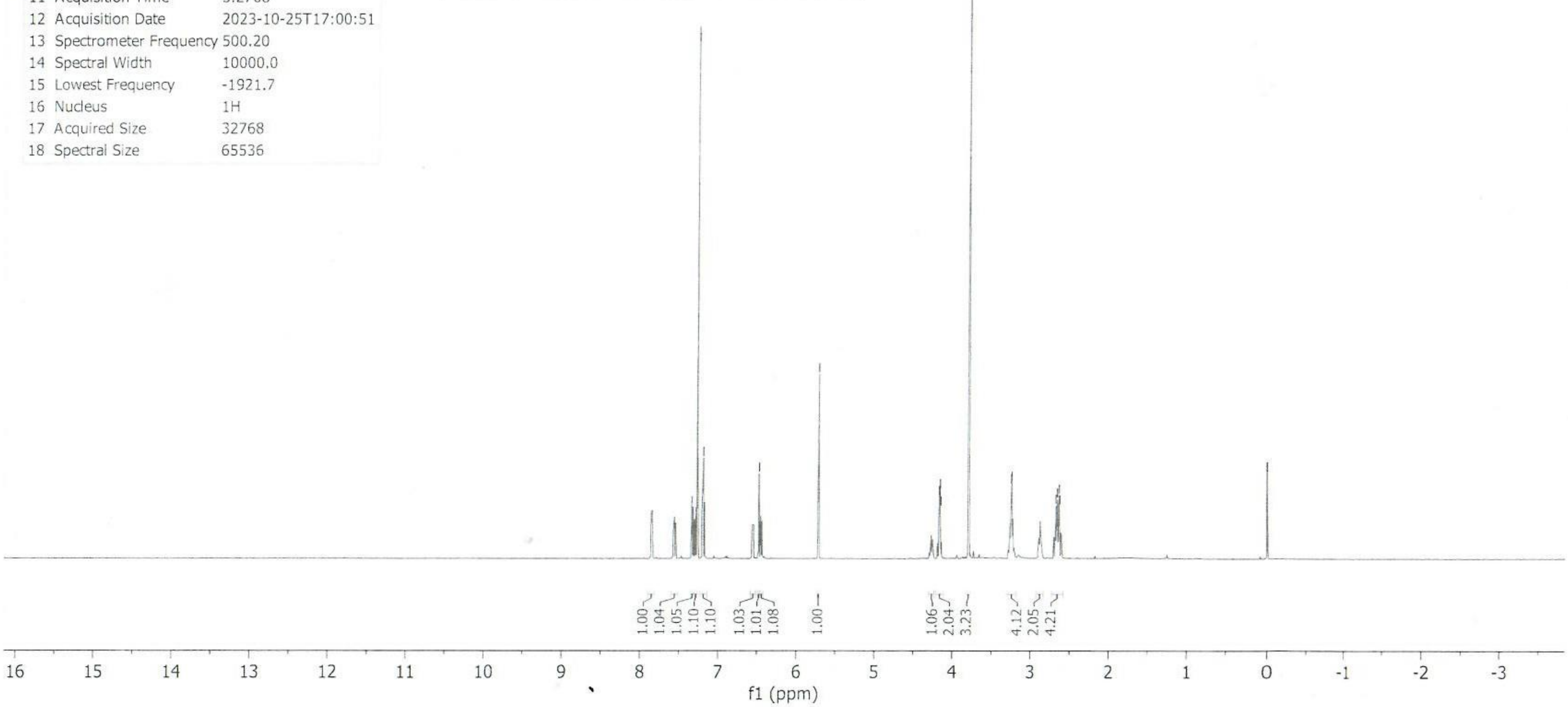

4

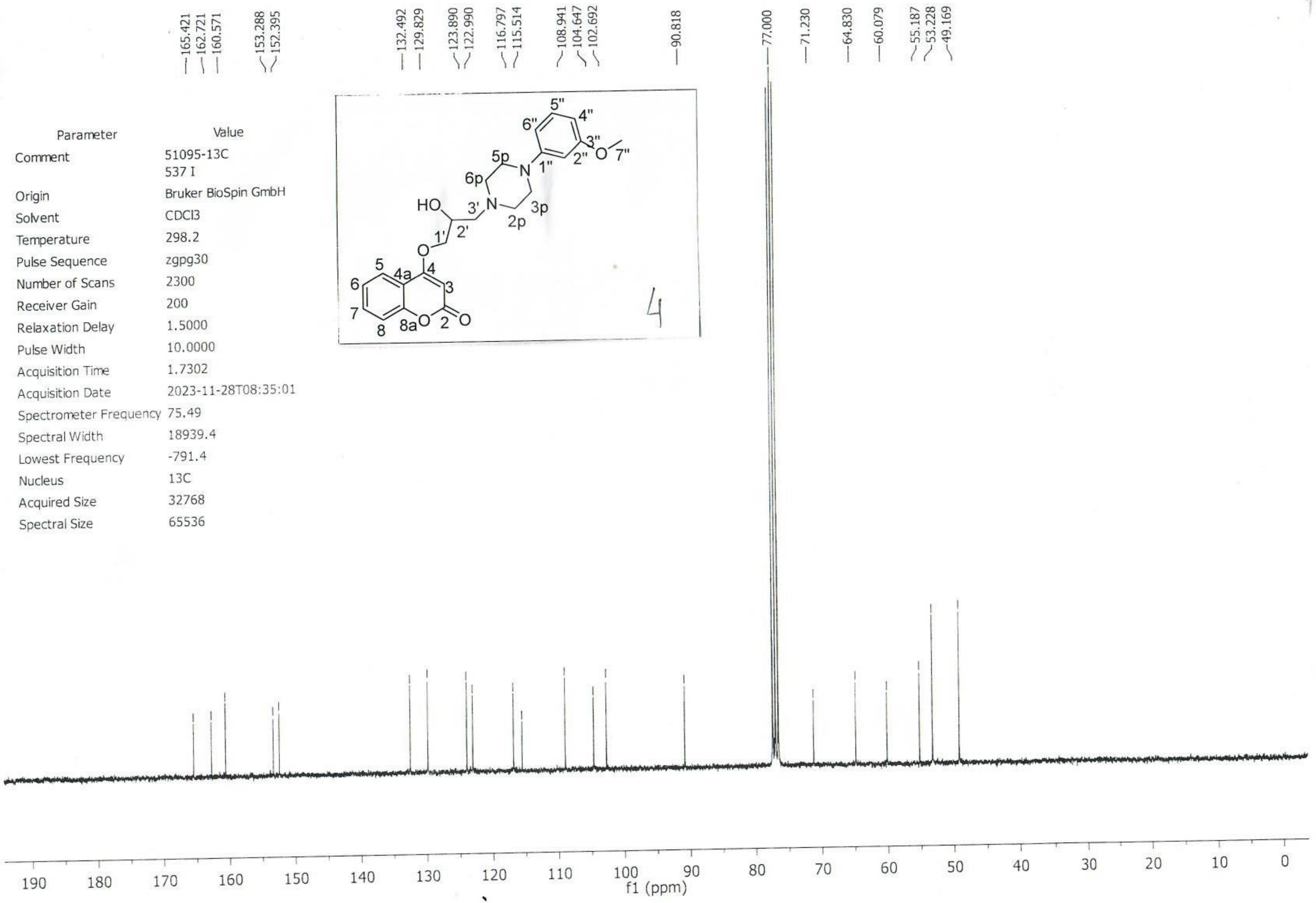

Parameter	Value
Comment	51095-13C<br>537 I
Origin	Bruker BioSpin GmbH
Solvent	CDCl3
Temperature	298.2
Pulse Sequence	zgpg30
Number of Scans	2300
Receiver Gain	200
Relaxation Delay	1.5000
Pulse Width	10.0000
Acquisition Time	1.7302
Acquisition Date	2023-11-28T08:35:01
Spectrometer Frequency	75.49
Spectral Width	18939.4
Lowest Frequency	-791.4
Nucleus	13C
Acquired Size	32768
Spectral Size	65536

5

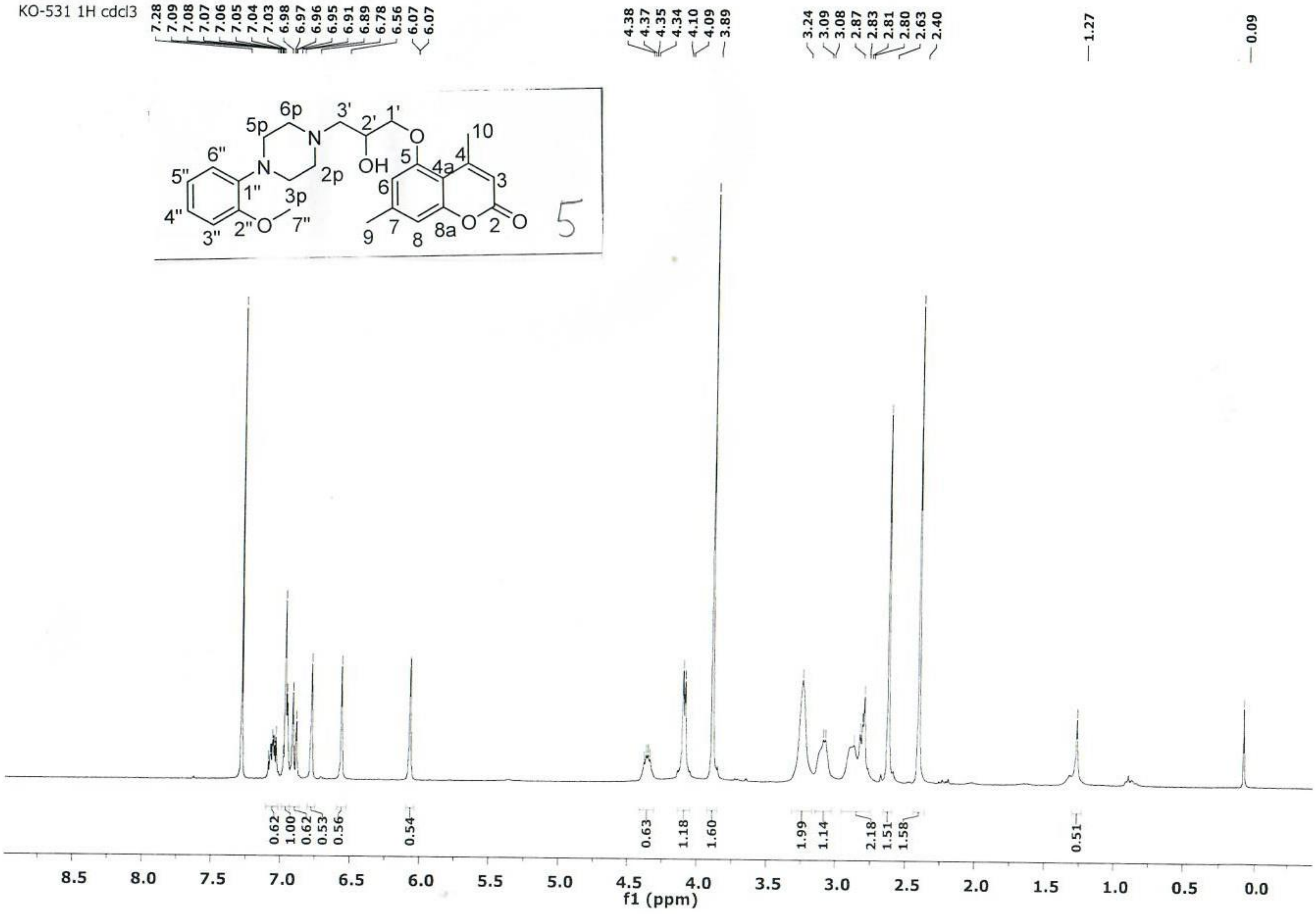

KO-531 1H cdcl3
7.28 7.09 7.08 7.07 7.06 7.05 7.04 7.03 6.98 6.97 6.96 6.95 6.91 6.89 6.78 6.56 6.07 6.07
4.38 4.37 4.35 4.34 4.10 4.09 3.89
3.24 3.09 3.08 2.87 2.83 2.81 2.80 2.63 2.40
1.27
0.09
6p 3' 1' 5p 2' O 10 6" 4 5 N N 4a OH 5" 3 1" 2p 6 3p 4" 2" O 7" 3" 9 7 8 8a O 2 O
5
0.62 1.00 0.62 0.53 0.56 0.54 0.63 1.18 1.60 1.99 1.14 2.18 1.51 1.58 0.51
8.5 8.0 7.5 7.0 6.5 6.0 5.5 5.0 4.5 4.0 3.5 3.0 2.5 2.0 1.5 1.0 0.5 0.0
f1 (ppm)

5

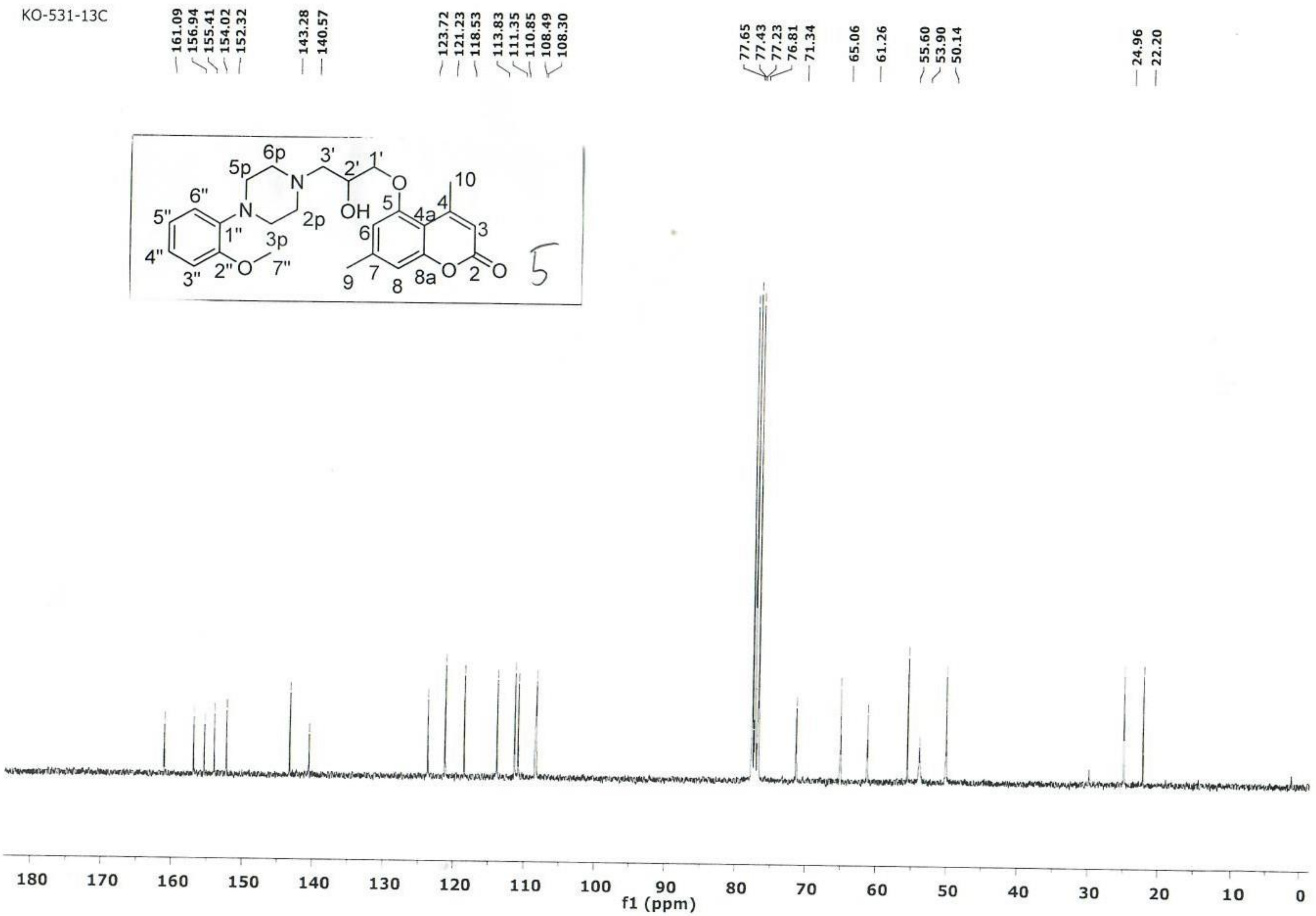
KO-531-13C
161.09
156.94
155.41
154.02
152.32
143.28
140.57
123.72
121.23
118.53
113.83
111.35
110.85
108.49
108.30
77.65
77.43
77.23
76.81
71.34
65.06
61.26
55.60
53.90
50.14
24.96
22.20
6p 3' 2' 1' 5p N O 10 6" OH 5 4 N 4a 5" 1" 2p 6 3 3p 4" 2" O 7" 3" 9 7 8 8a O 2 O 5
180 170 160 150 140 130 120 110 100 90 80 70 60 50 40 30 20 10 0
f1 (ppm)

6

Parameter	Value
Comment	38216-1H<br>KO433a
Origin	Bruker BioSpin GmbH
Solvent	CDCl3
Temperature	0.0
Pulse Sequence	zg30
Number of Scans	32
Receiver Gain	129
Relaxation Delay	0.0000
Pulse Width	14.5000
Acquisition Time	3.5001
Acquisition Date	2021-05-14T09:40:50
Spectrometer Frequency	300.20
Spectral Width	6009.6
Lowest Frequency	-1154.9
Nucleus	1H
Acquired Size	21034
Spectral Size	65536

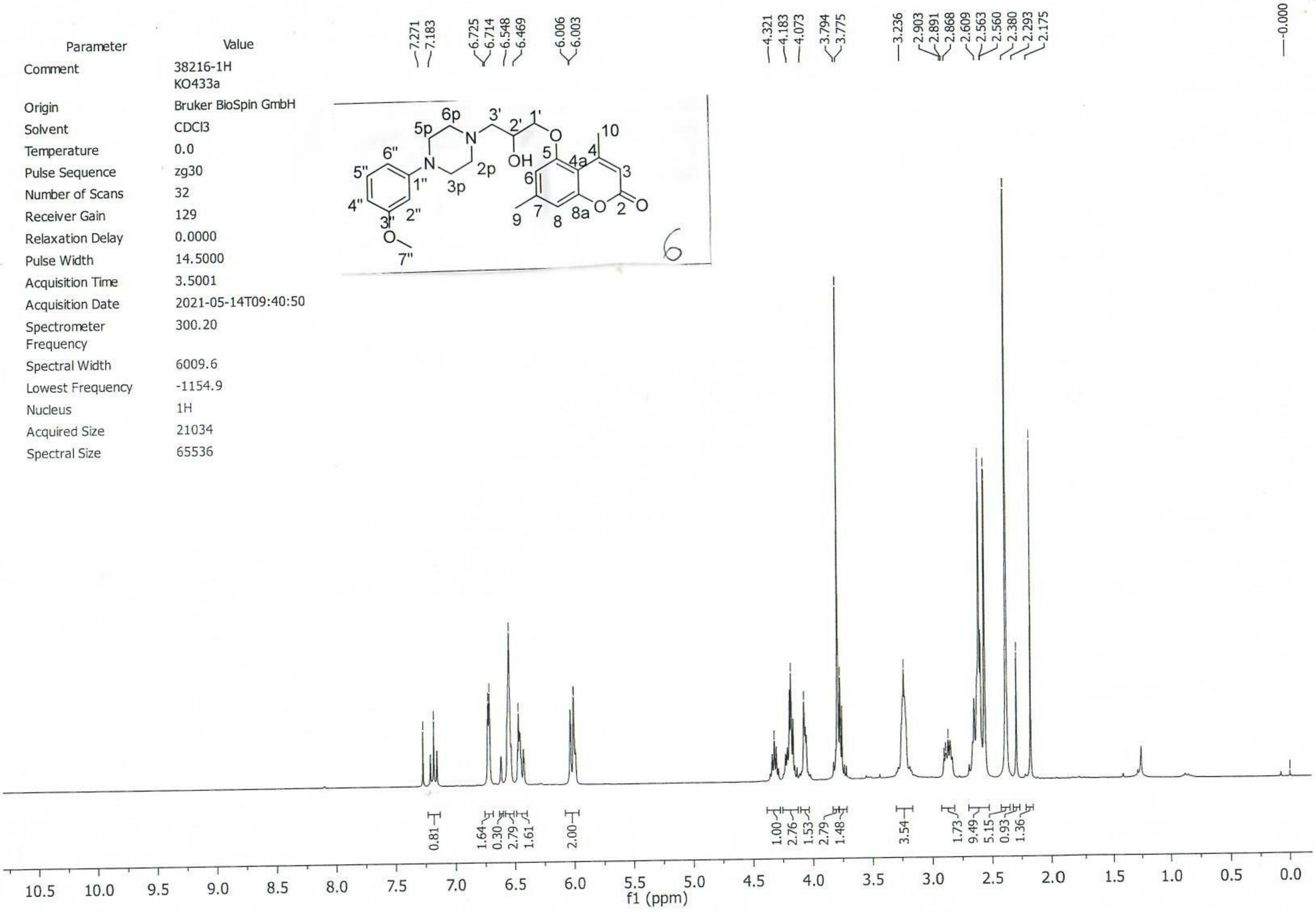

Parameter	Value
Comment	38216-13C<br>KO433a
Origin	Bruker BioSpin GmbH
Solvent	CDCl3
Temperature	298.2
Pulse Sequence	zgpg30
Number of Scans	3600
Receiver Gain	200
Relaxation Delay	1.5000
Pulse Width	10.0000
Acquisition Time	1.7302
Acquisition Date	2021-05-25T21:46:19
Spectrometer Frequency	75.49
Spectral Width	18939.4
Lowest Frequency	-794.8
Nucleus	13C
Acquired Size	32768
Spectral Size	65536

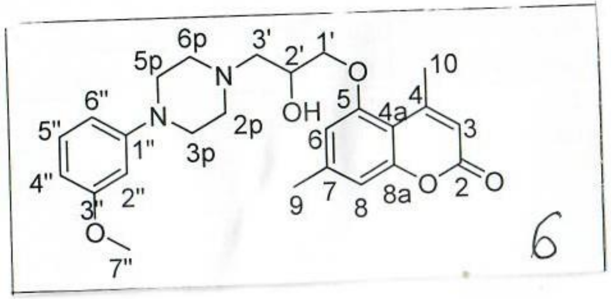

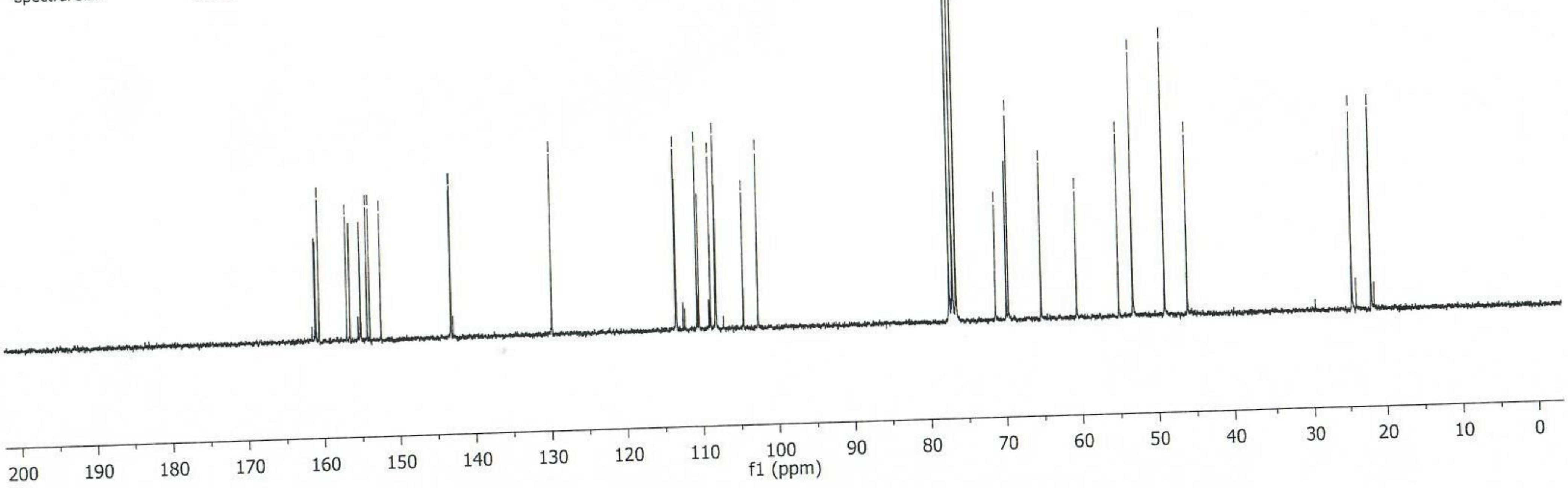

6

7

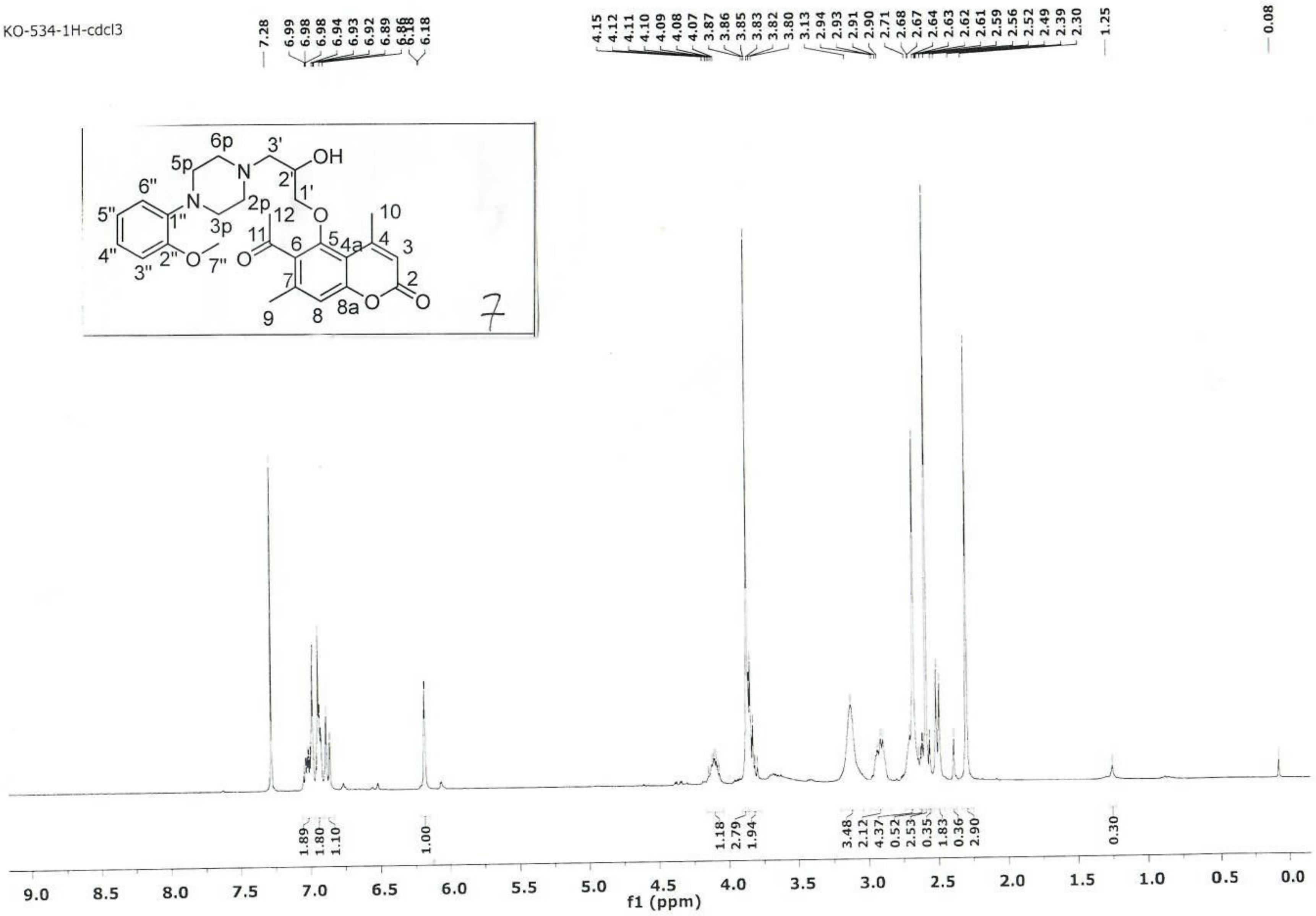
KO-534-1H-cdcl3
7.28
6.99
6.98
6.98
6.94
6.93
6.92
6.89
6.86
6.18
6.18
4.15
4.12
4.11
4.10
4.09
4.08
4.07
3.87
3.86
3.85
3.83
3.82
3.80
3.13
2.94
2.93
2.91
2.90
2.71
2.68
2.67
2.64
2.63
2.62
2.61
2.59
2.56
2.52
2.49
2.39
2.30
1.25
0.08
6p
3'
OH
5p
N
2'
6"
1'
N
2p
12
10
5"
1"
3p
O
11
5
4a
4
3
6
4"
2"
O
7"
O
7
2
9
8
8a
O
O
7
1.89
1.80
1.10
1.00
1.18
2.79
1.94
3.48
2.12
4.37
0.52
2.53
0.35
1.83
0.36
2.90
0.30
9.0
8.5
8.0
7.5
7.0
6.5
6.0
5.5
5.0
4.5
f1 (ppm)
4.0
3.5
3.0
2.5
2.0
1.5
1.0
0.5
0.0

7

KO-534-13C

204.84

160.15
154.71
153.90
152.59
152.27

140.87
139.27
133.59

123.39
121.11
118.35
116.00
115.48
112.71
111.27

80.44
77.65
77.43
77.23
76.81

65.37
59.89
55.50
53.54
50.55

32.92

22.98
19.48

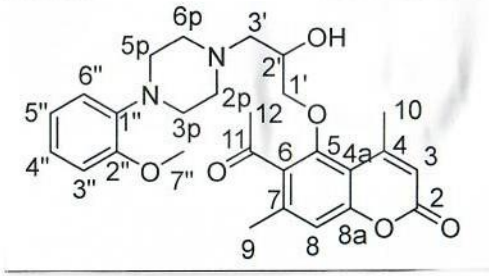

7

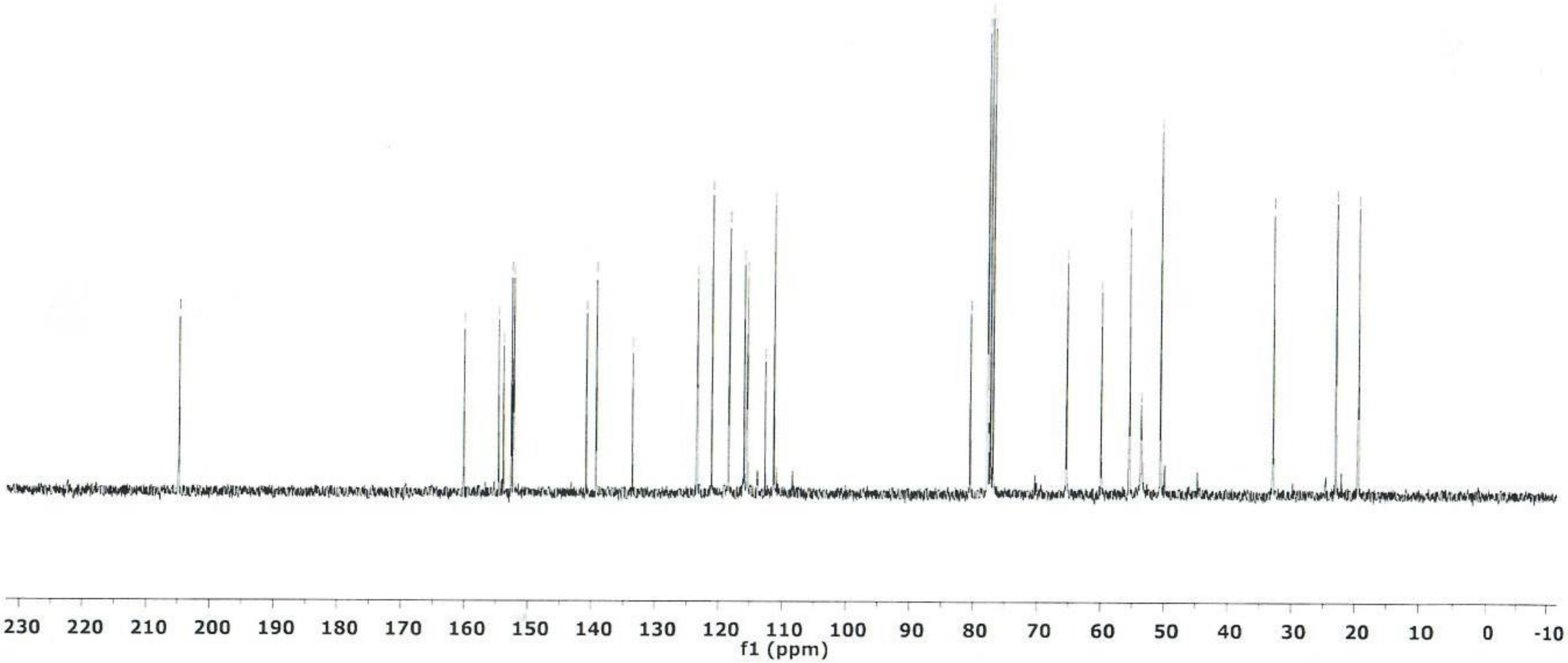

9184-1H
539 IB

Parameters		
	Parameter	Value
1	Title	
2	Owner	felix
3	Solvent	CDCl3
4	Temperature	298.2
5	Pulse Sequence	zg30
6	Experiment	1D
7	Number of Scans	8
8	Receiver Gain	29.7
9	Relaxation Delay	0.0000
10	Pulse Width	11.3000
11	Acquisition Time	3.2768
12	Acquisition Date	2023-10-25T17:34:26
13	Spectrometer Frequency	500.20
14	Spectral Width	10000.0
15	Lowest Frequency	-1919.4
16	Nucleus	1H
17	Acquired Size	32768
18	Spectral Size	65536

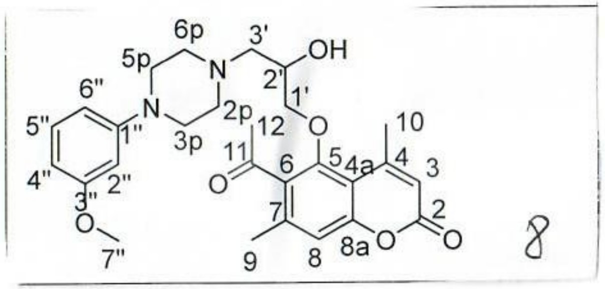

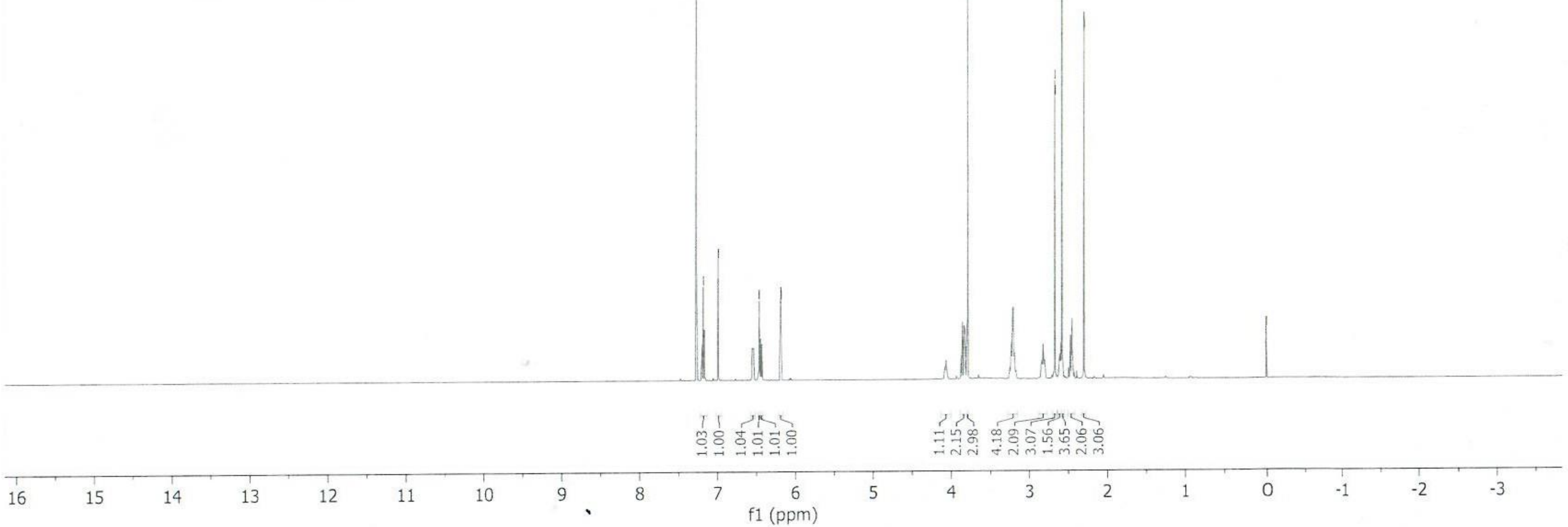

Parameter	Value
Comment	51092-13C<br>539 IB
Origin	Bruker BioSpin GmbH
Solvent	CDCl3
Temperature	298.1
Pulse Sequence	zgpg30
Number of Scans	2300
Receiver Gain	200
Relaxation Delay	1.5000
Pulse Width	10.0000
Acquisition Time	1.7302
Acquisition Date	2023-11-28T02:08:50
Spectrometer Frequency	75.49
Spectral Width	18939.4
Lowest Frequency	-792.0
Nucleus	13C
Acquired Size	32768
Spectral Size	65536

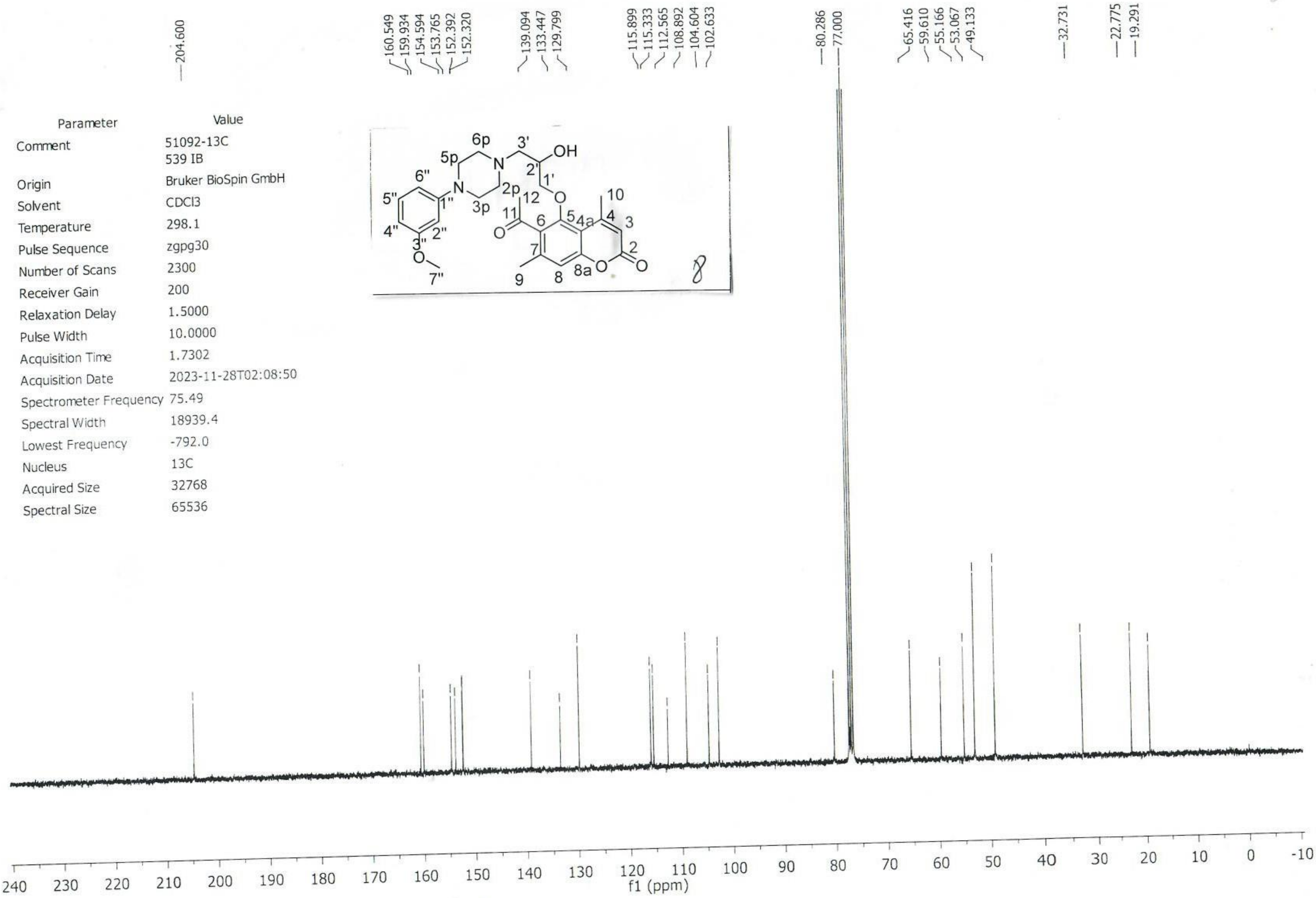

9

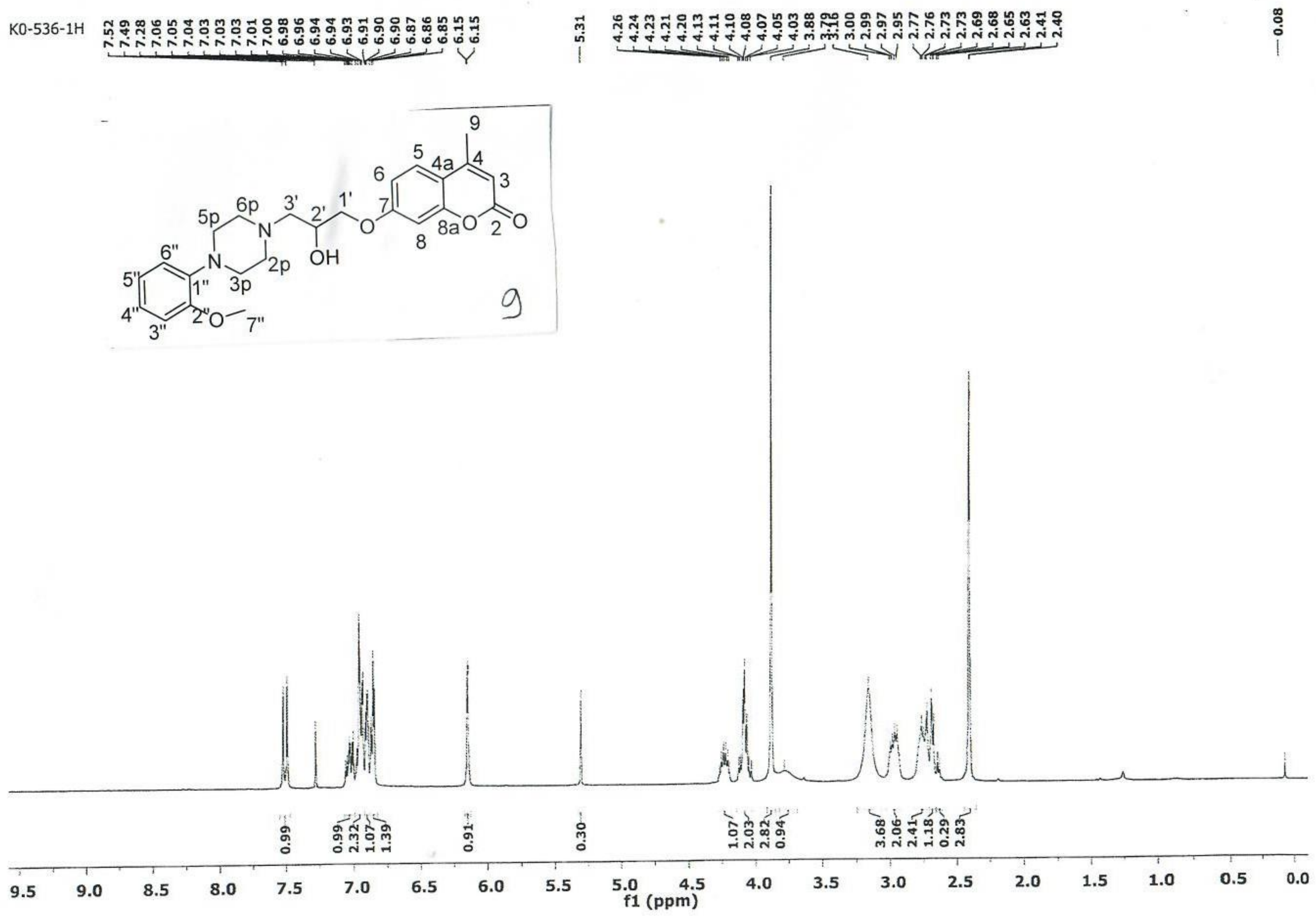
K0-536-1H
7.52 7.49 7.28 7.06 7.05 7.04 7.03 7.03 7.03 7.01 7.00 6.98 6.96 6.94 6.94 6.93 6.91 6.90 6.90 6.87 6.86 6.85 6.15 6.15
5.31
4.26 4.24 4.23 4.21 4.20 4.13 4.11 4.10 4.08 4.07 4.05 4.03 3.88 3.79 3.16 3.00 2.99 2.97 2.95 2.77 2.76 2.73 2.73 2.69 2.68 2.65 2.63 2.41 2.40
0.08
9
5
4a
4
3
6
3'
1'
7
6p
2'
5p
N
O
O
8a
O
2
8
6"
N
2p
OH
5"
1"
3p
4"
2"
O
7"
3"
9
0.99
0.99 2.32 1.07 1.39
0.91
0.30
1.07 2.03 2.82 0.94
3.68 2.06 2.41 1.18 0.29 2.83
9.5 9.0 8.5 8.0 7.5 7.0 6.5 6.0 5.5 5.0 4.5 4.0 3.5 3.0 2.5 2.0 1.5 1.0 0.5 0.0
f1 (ppm)

9

K0-536-13C

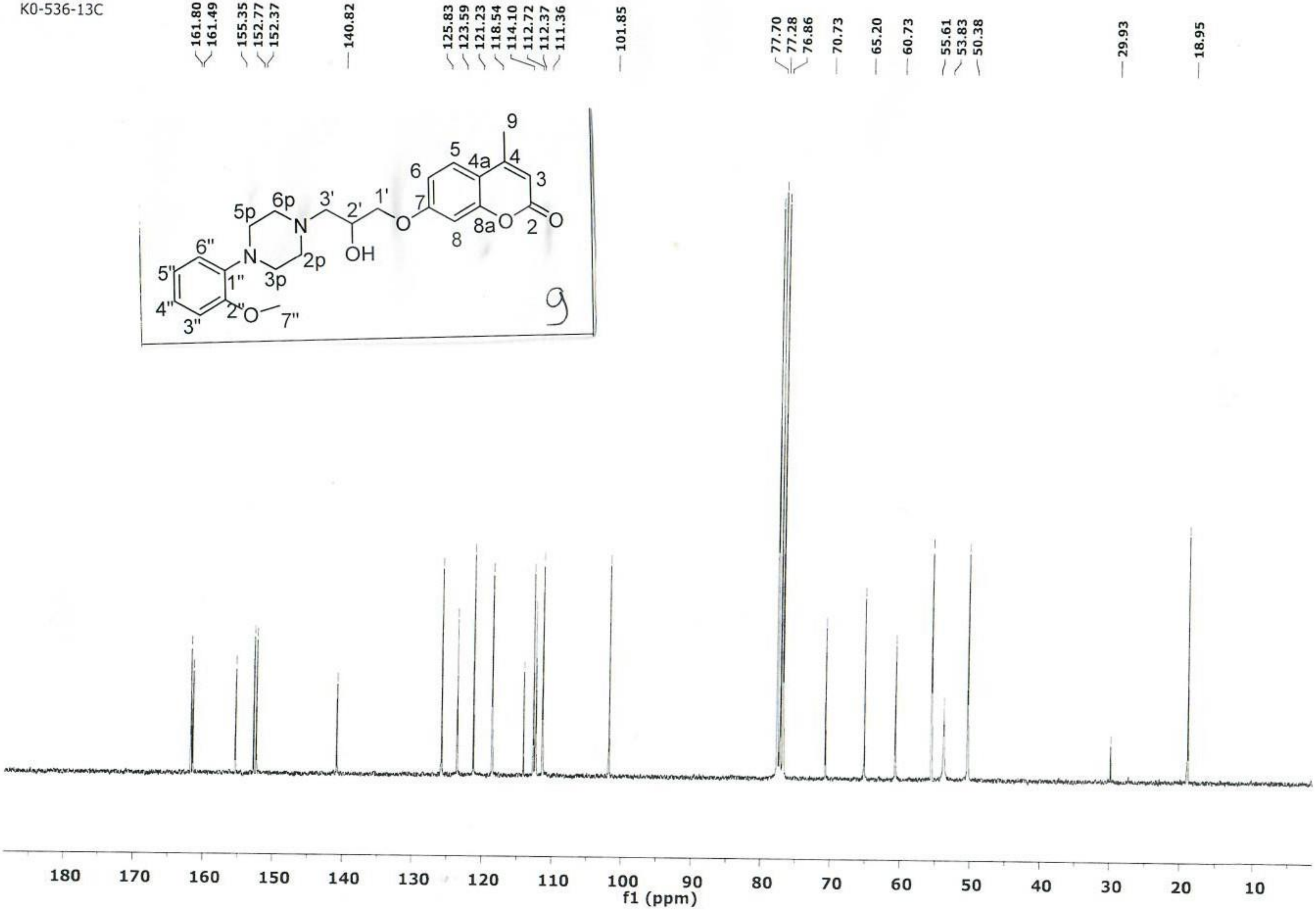

161.80
161.49
155.35
152.77
152.37
140.82
125.83
123.59
121.23
118.54
114.10
112.72
112.37
111.36
101.85
77.70
77.28
76.86
70.73
65.20
60.73
55.61
53.83
50.38
29.93
18.95
9
5
4a
4
3
6
7
2
O
8a
O
8
5p
6p
N
3'
2'
1'
O
OH
6''
N
2p
5''
1''
3p
4''
2''
O
7''
3''
9
180
170
160
150
140
130
120
110
100
90
80
70
60
50
40
30
20
10
f1 (ppm)

9187-1H
540 II

Parameters

	Parameter	Value
1	Title	
2	Owner	felix
3	Solvent	CDCl3
4	Temperature	298.2
5	Pulse Sequence	zg30
6	Experiment	1D
7	Number of Scans	8
8	Receiver Gain	29.7
9	Relaxation Delay	0.0000
10	Pulse Width	11.3000
11	Acquisition Time	3.2768
12	Acquisition Date	2023-10-25T17:41:16
13	Spectrometer Frequency	500.20
14	Spectral Width	10000.0
15	Lowest Frequency	-1922.0
16	Nucleus	1H
17	Acquired Size	32768
18	Spectral Size	65536

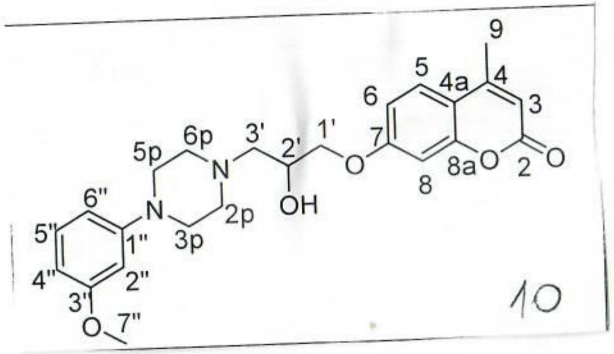

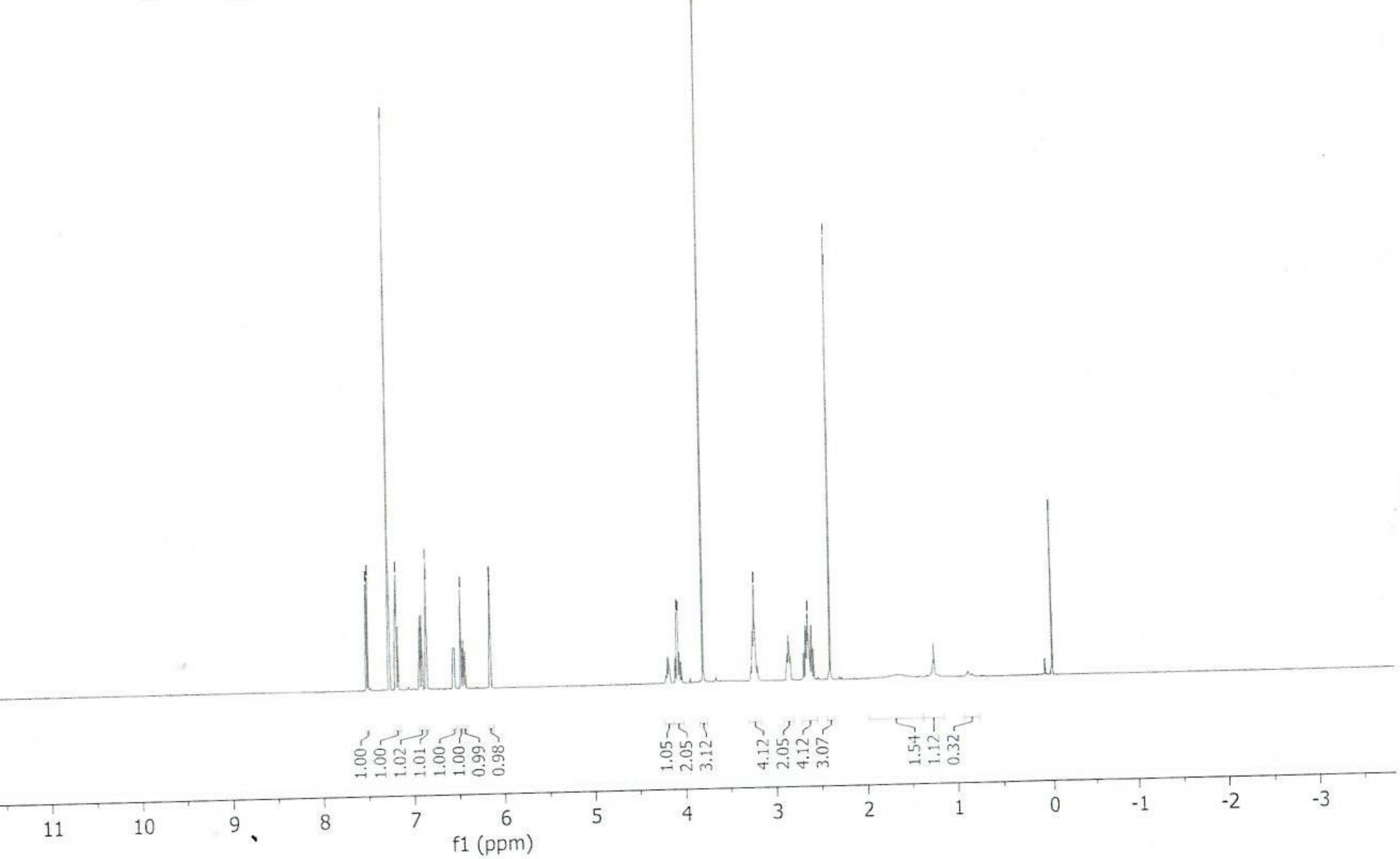

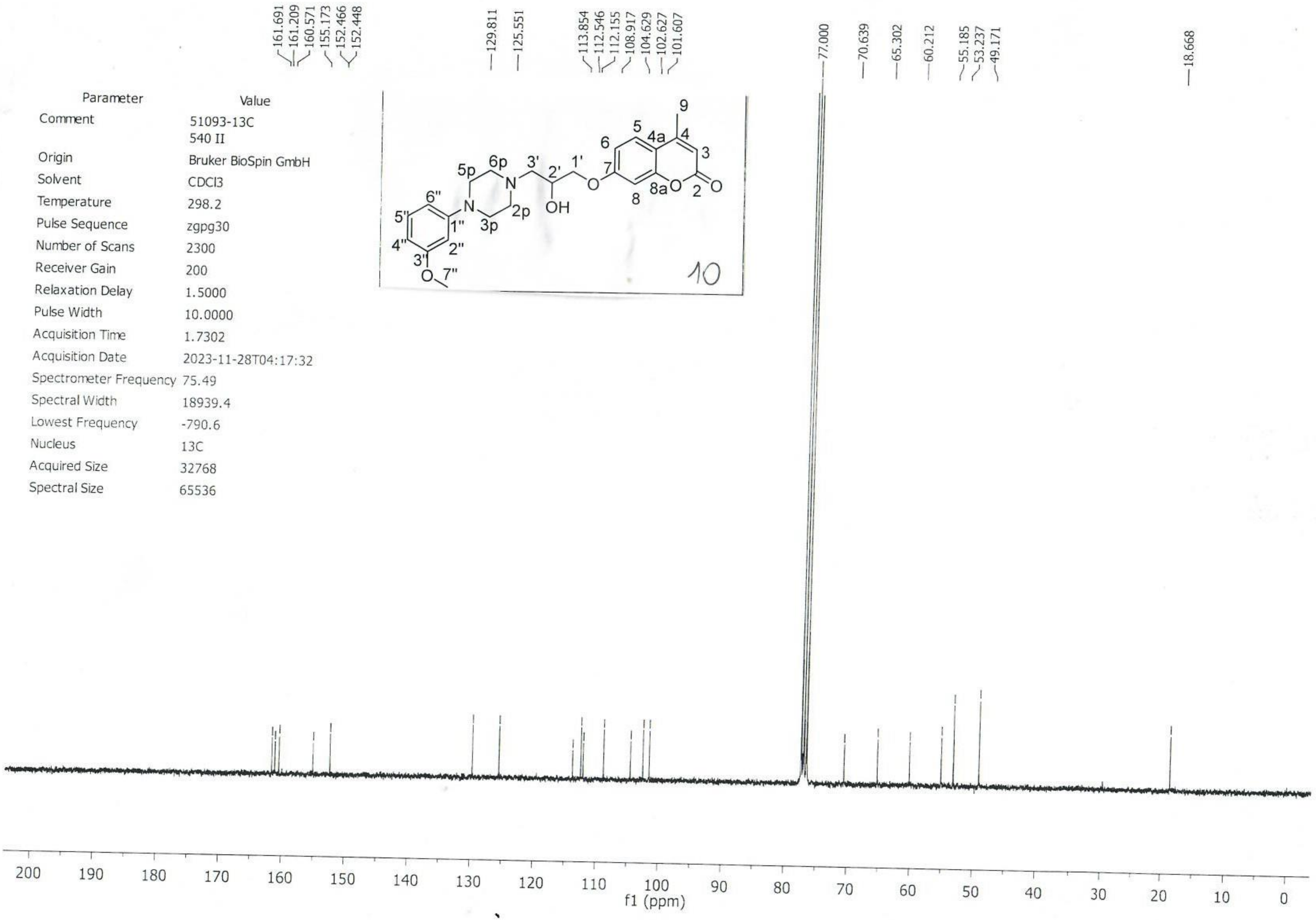

Parameter	Value
Comment	51093-13C<br>540 II
Origin	Bruker BioSpin GmbH
Solvent	CDCl3
Temperature	298.2
Pulse Sequence	zgpg30
Number of Scans	2300
Receiver Gain	200
Relaxation Delay	1.5000
Pulse Width	10.0000
Acquisition Time	1.7302
Acquisition Date	2023-11-28T04:17:32
Spectrometer Frequency	75.49
Spectral Width	18939.4
Lowest Frequency	-790.6
Nucleus	13C
Acquired Size	32768
Spectral Size	65536

9191-1H
541 II

11.

Parameters		
	Parameter	Value
1	Title	
2	Owner	felix
3	Solvent	CDCl3
4	Temperature	298.2
5	Pulse Sequence	zg30
6	Experiment	1D
7	Number of Scans	8
8	Receiver Gain	29.7
9	Relaxation Delay	0.0000
10	Pulse Width	11.3000
11	Acquisition Time	3.2768
12	Acquisition Date	2023-10-25T18:10:13
13	Spectrometer Frequency	500.20
14	Spectral Width	10000.0
15	Lowest Frequency	-1921.5
16	Nucleus	1H
17	Acquired Size	32768
18	Spectral Size	65536

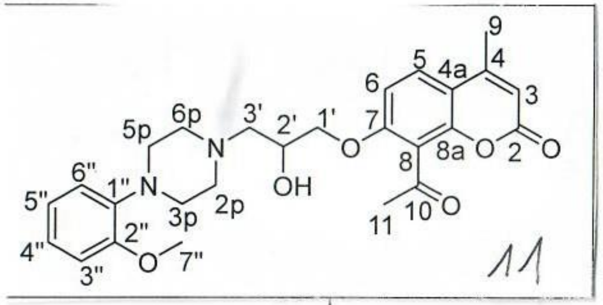

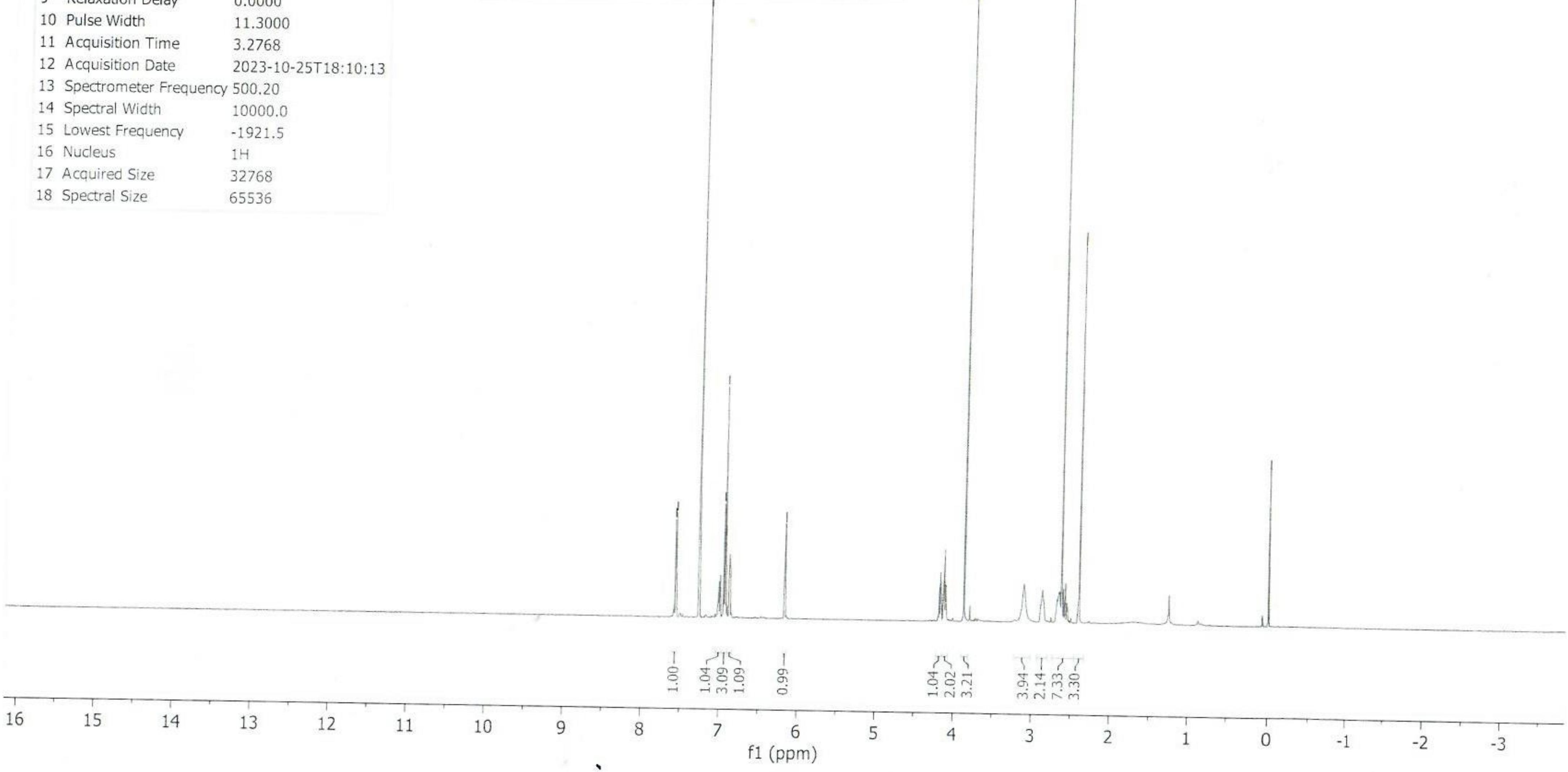

Parameter	Value
Comment	51094-13C<br>541 II
Origin	Bruker BioSpin GmbH
Solvent	CDCl3
Temperature	298.2
Pulse Sequence	zgpg30
Number of Scans	2300
Receiver Gain	200
Relaxation Delay	1.5000
Pulse Width	10.0000
Acquisition Time	1.7302
Acquisition Date	2023-11-28T06:26:15
Spectrometer Frequency	75.49
Spectral Width	18939.4
Lowest Frequency	-790.7
Nucleus	13C
Acquired Size	32768
Spectral Size	65536

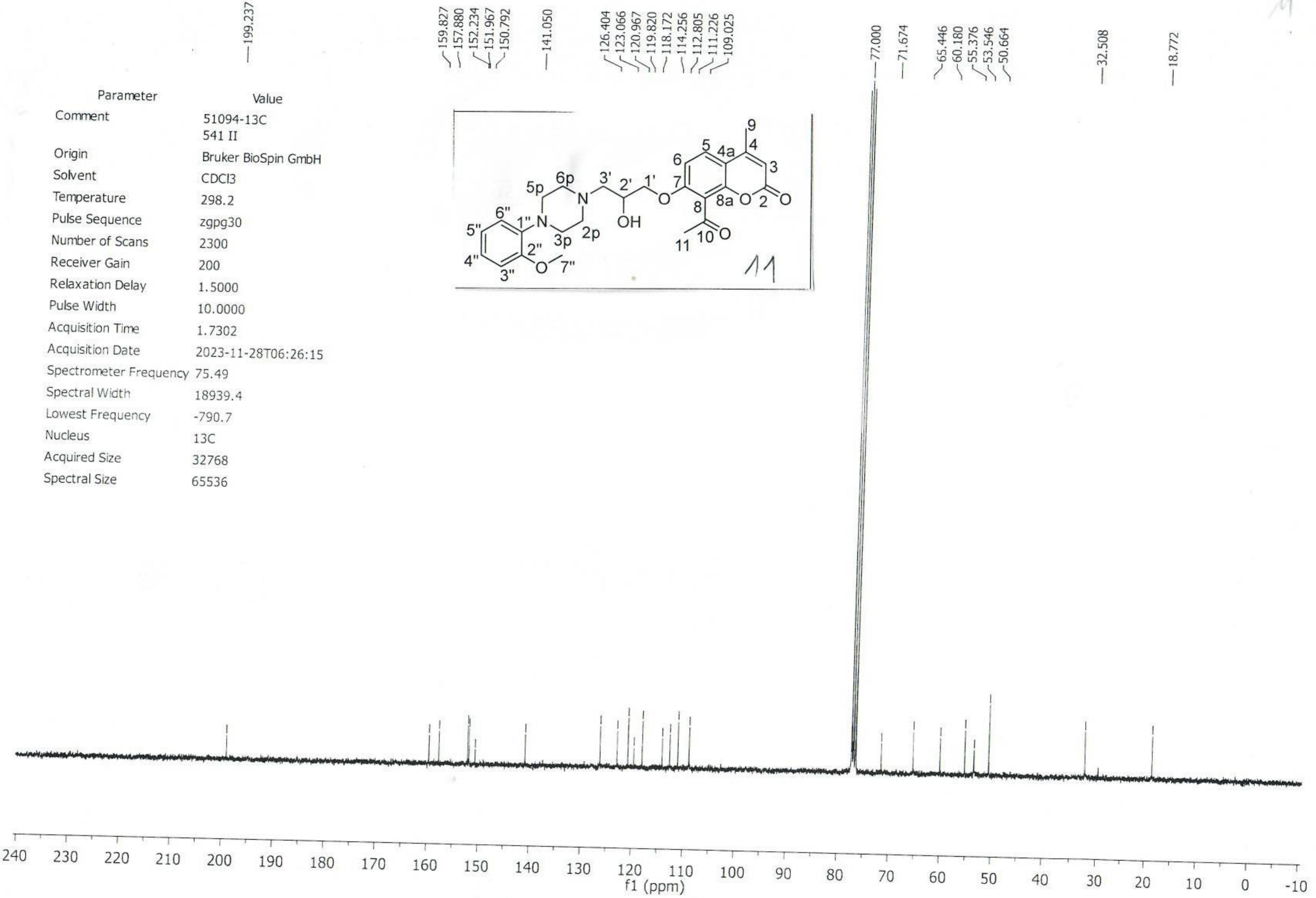

9194-1H
542 II

Parameters

	Parameter	Value
1	Title	
2	Owner	felix
3	Solvent	CDCl3
4	Temperature	298.2
5	Pulse Sequence	zg30
6	Experiment	1D
7	Number of Scans	8
8	Receiver Gain	29.7
9	Relaxation Delay	0.0000
10	Pulse Width	11.3000
11	Acquisition Time	3.2768
12	Acquisition Date	2023-10-25T18:17:52
13	Spectrometer Frequency	500.20
14	Spectral Width	10000.0
15	Lowest Frequency	-1921.2
16	Nucleus	1H
17	Acquired Size	32768
18	Spectral Size	65536

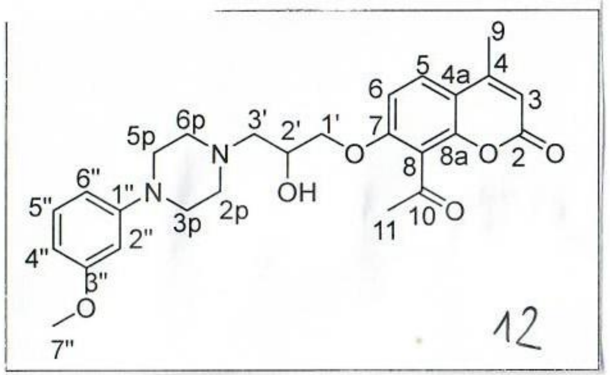

12

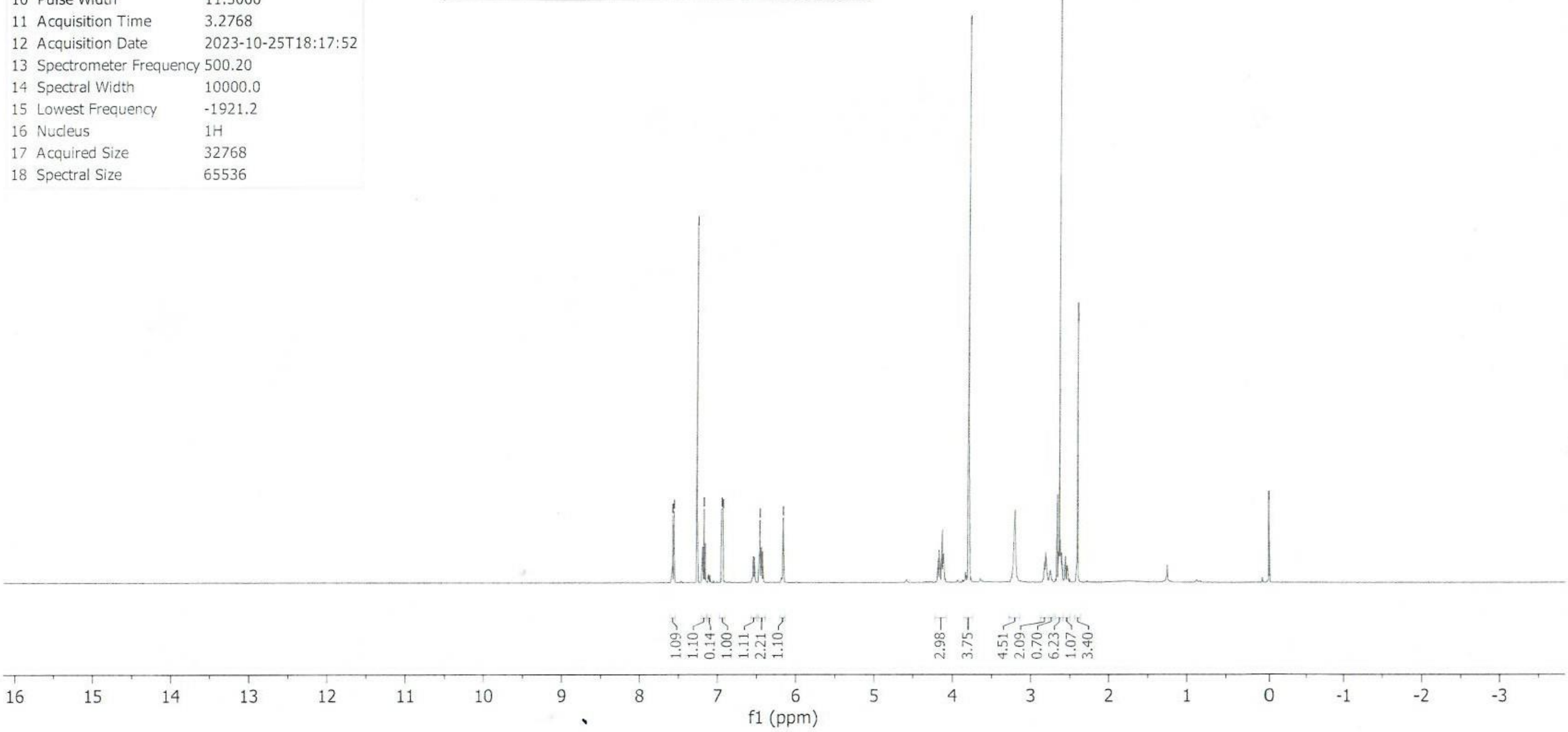

12

Parameter	Value
Comment	51091-13C<br>542 II
Origin	Bruker BioSpin GmbH
Solvent	CDCl3
Temperature	298.2
Pulse Sequence	zgpg30
Number of Scans	2300
Receiver Gain	200
Relaxation Delay	1.5000
Pulse Width	10.0000
Acquisition Time	1.7302
Acquisition Date	2023-11-27T23:59:49
Spectrometer Frequency	75.49
Spectral Width	18939.4
Lowest Frequency	-791.1
Nucleus	13C
Acquired Size	32768
Spectral Size	65536

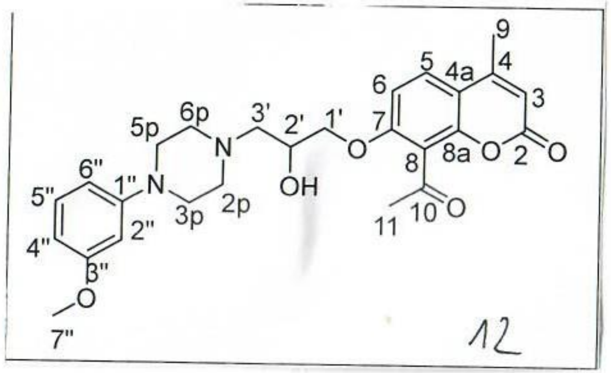

12

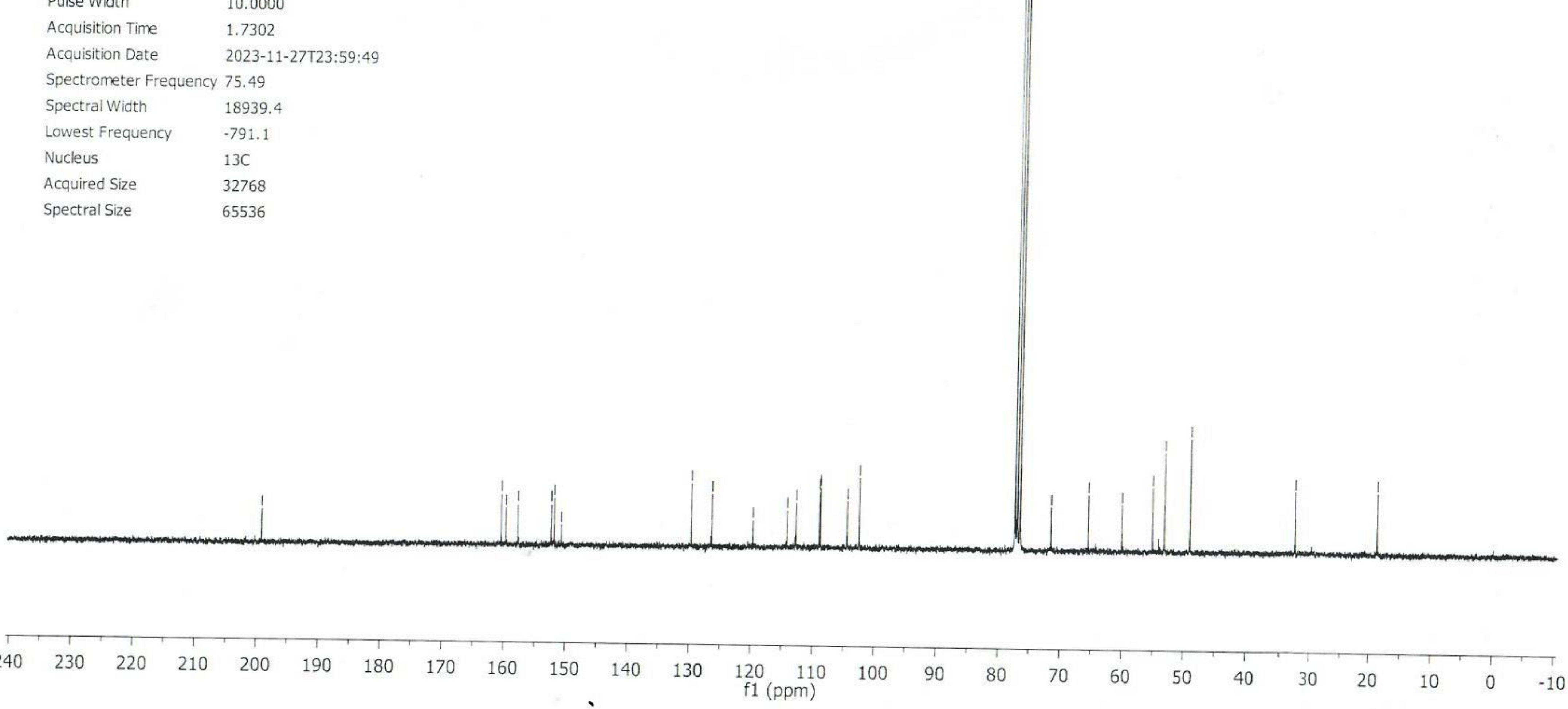

Parameter	Value
Comment	52096-13C<br>543 I
Origin	Bruker BioSpin GmbH
Solvent	CDCl3
Temperature	298.1
Pulse Sequence	zgpg30
Number of Scans	2400
Receiver Gain	200
Relaxation Delay	1.5000
Pulse Width	10.0000
Acquisition Time	1.7302
Acquisition Date	2023-12-05T19:59:01
Spectrometer Frequency	75.49
Spectral Width	18939.4
Lowest Frequency	-790.9
Nucleus	13C
Acquired Size	32768
Spectral Size	65536

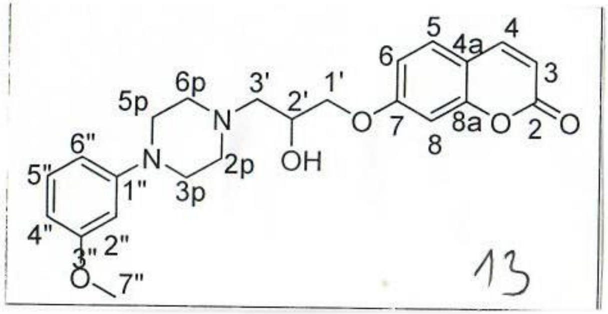

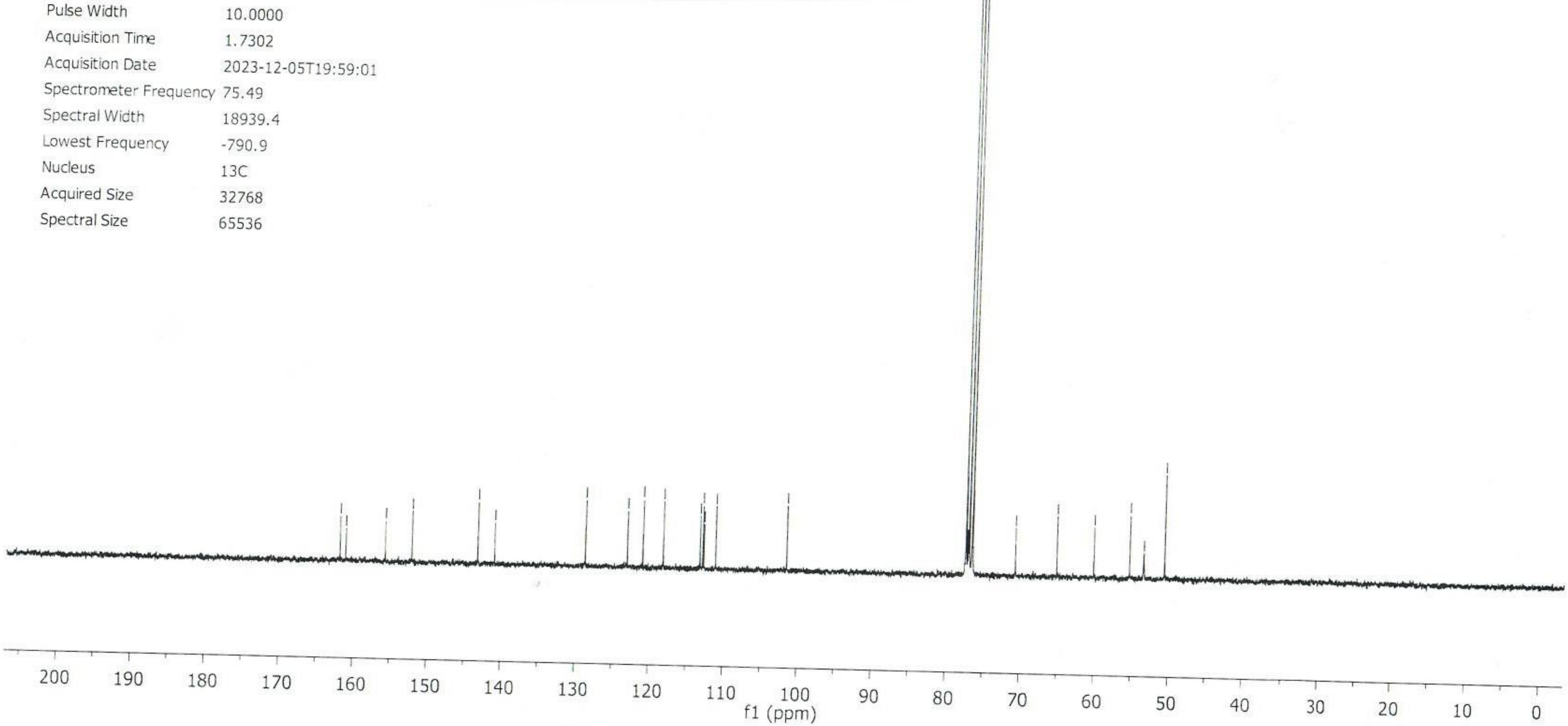

Parameter	Value
Comment	52097-1H<br>544 I
Origin	Bruker BioSpin GmbH
Solvent	CDCl3
Temperature	298.1
Pulse Sequence	zg30
Number of Scans	44
Receiver Gain	200
Relaxation Delay	0.0000
Pulse Width	14.5000
Acquisition Time	3.5001
Acquisition Date	2023-11-28T14:34:49
Spectrometer Frequency	300.20
Spectral Width	6009.6
Lowest Frequency	-1156.7
Nucleus	1H
Acquired Size	21034
Spectral Size	65536

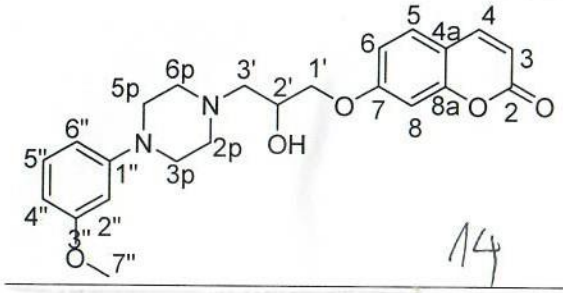

14

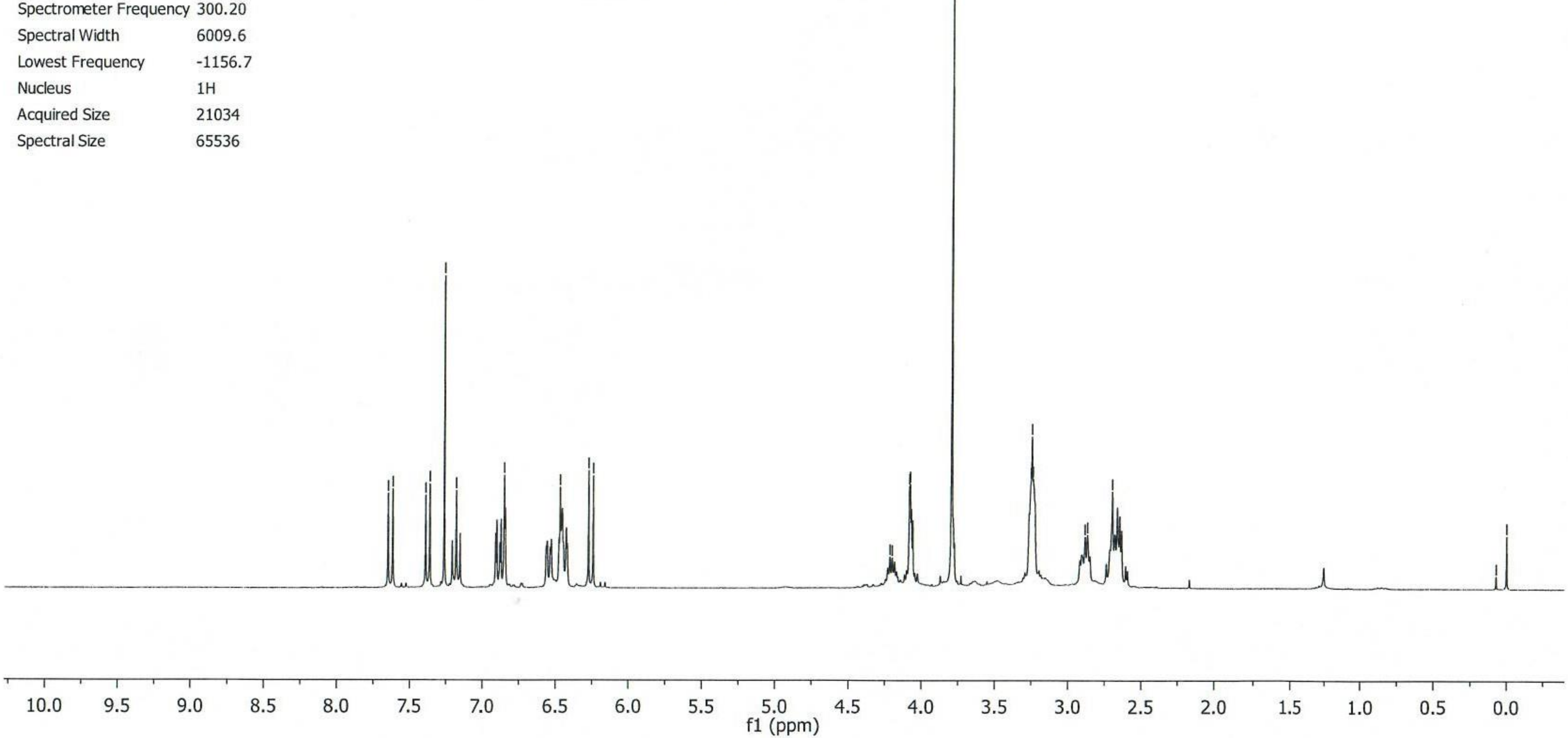

14

Parameter	Value
Comment	53088-1H<br>544I
Origin	Bruker BioSpin GmbH
Solvent	CDCl3
Temperature	298.1
Pulse Sequence	zgpg30
Number of Scans	4800
Receiver Gain	200
Relaxation Delay	1.5000
Pulse Width	10.0000
Acquisition Time	1.7302
Acquisition Date	2024-02-22T22:15:59
Spectrometer Frequency	75.49
Spectral Width	18939.4
Lowest Frequency	-788.9
Nucleus	13C
Acquired Size	32768
Spectral Size	65536

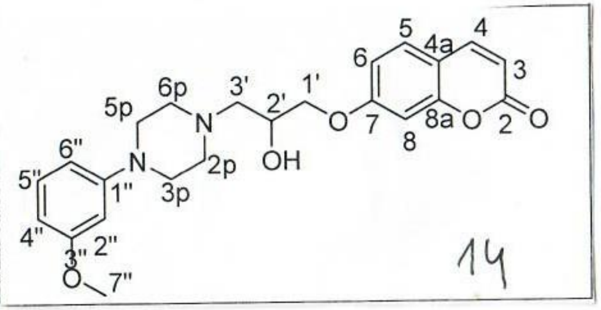

14

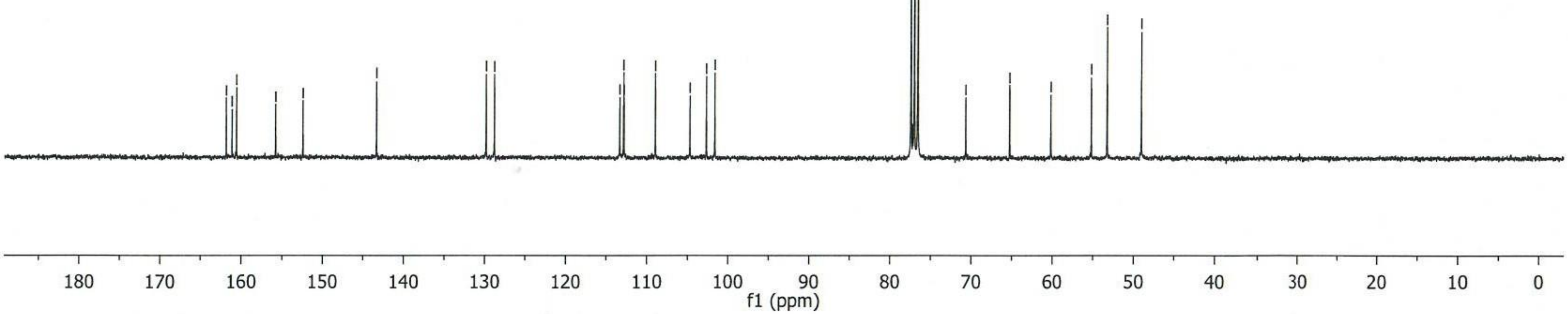

Supplementary data

Binding affinities were calculated according to the Cheng-Prusoff equation:

$$Ki = \frac{IC50}{1+\frac{[R]}{Kd}}$$

K_i – inhibitory constant; IC_{50} – half maximal inhibitory concentration; [R] – radioligand concentration in the assay in nM; K_d – radioligand dissociation constant

Tables S1-S3: Percent of specific [^{3}H]ketanserin displacement data used to calculate K_i values of the compounds at the 5-HT_{2A} receptor

**Table S1**	**9**			**8**			**7**			**3**			**5**		
**Conc. [M]**	**Mean**	**SEM**	**n**	**Mean**	**SEM**	**n**	**Mean**	**SEM**	**n**	**Mean**	**SEM**	**n**	**Mean**	**SEM**	**n**
**-11**	100.87	0.44	3	100.87	0.44	3	100.25	17.70	3	100.00	3.97	3	100.00	4.31	3
**-10**	105.22	2.71	3	92.30	3.13	3	101.98	6.66	3	92.31	9.38	3	100.00	4.31	3
**-9**	104.42	16.91	3	99.14	4.00	3	102.94	9.26	3	90.11	5.86	3	102.61	7.54	3
**-8**	88.97	7.09	2	109.66	0.27	3	102.24	4.68	2	91.21	15.22	2	100.00	0.86	3
**-7**	74.96	6.64	3	62.70	13.77	3	94.40	3.98	2	74.73	9.77	3	84.70	2.80	3
**-6**	34.54	2.74	3	3.79	15.10	3	67.76	2.59	3	70.33	5.08	3	67.91	9.48	3
**-5**	0.67	9.01	3	5.95	8.44	3	46.21	4.81	3	40.66	9.77	3	35.45	0.22	3
**-4**	-9.23	2.25	3	-13.70	1.39	2	4.61	5.18	3	28.57	9.47	2	35.07	0.43	3

Table S2	4			2			1			6			7		
**Conc. [M]**	**Mean**	**SEM**	**n**	**Mean**	**SEM**	**n**	**Mean**	**SEM**	**n**	**Mean**	**SEM**	**n**	**Mean**	**SEM**	**n**
-11	100.70	11.27	3	100.71	11.27	3	100.94	11.27	3	100.91	11.27	3	100.51	0.00	3
-10	102.43	16.32	3	92.22	9.42	3	98.59	4.62	2	97.83	7.53	3	103.03	5.25	3
-9	105.02	5.65	3	87.94	4.28	3	100.24	0.94	3	94.02	4.71	3	106.06	12.12	2
-8	106.75	8.29	2	86.77	0.00	1	116.10	5.33	3	105.98	14.12	3	102.27	5.69	3
-7	92.80	15.69	3	48.61	13.43	3	90.11	12.23	2	79.89	7.84	3	-14.39	4.81	3
-6	22.31	4.39	3	27.40	10.49	3	52.78	7.84	3	44.02	4.08	3	-14.39	4.81	3
-5	-0.74	2.45	2	34.85	6.91	3	44.96	5.16	2	32.07	7.84	3	-42.42	2.62	3
-4	4.89	9.73	3	-13.01	4.76	3	4.23	15.06	3	0.91	7.58	3	-5.30	12.88	2

Table S3	14			10			11			12		
**Conc [M]**	**Mean**	**SEM**	**n**	**Mean**	**SEM**	**n**	**Mean**	**SEM**	**n**	**Mean**	**SEM**	**n**
-11	100.05	13.60	3	100.66	21.69	3	100.66	21.69	3	100.51	0.00	3
-10	104.92	1.33	3	86.90	0.69	3	114.88	1.72	3	96.97	8.75	3
-9	92.62	5.77	3	82.14	0.00	2	103.57	7.56	3	98.48	0.87	3
-8	109.54	0.44	3	88.69	1.03	3	101.19	4.81	3	65.15	9.09	2
-7	54.15	10.21	3	66.67	10.31	3	110.42	4.46	2	66.67	9.09	2
-6	-15.85	4.44	3	40.48	8.25	3	80.36	4.47	3	-9.85	0.44	3
-5	-2.77	2.22	3	-8.93	2.41	3	51.19	0.00	3	-26.14	10.98	2
-4	0.05	10.60	3	-16.67	0.69	3	1.19	4.81	3	-11.74	8.71	2

Conc. [M] – compound concentration in mol/L; Mean – mean of non-specific binding subtracted percent of [^{3}H]ketanserin displacement by the analyzed compounds from 2-3 experiments; SEM – standard error of the mean; n – number of separate experiments

Tables S4-S6: Percent of specific [^{3}H]8-OH-DPAT displacement data used to calculate K_i values of the compounds at the 5-HT_{1A} receptor

**Table S4**	**9**			**8**			**7**			**6**			**11**		
**Conc. [M]**	**Mean**	**SEM**	**n**	**Mean**	**SEM**	**n**	**Mean**	**SEM**	**n**	**Mean**	**SEM**	**n**	**Mean**	**SEM**	**n**
-11	100,00	10,85	3	100,24	18,13	2	100,14	2,35	3	100,00	9,52	3	100,18	6,45	3
-10	104,22	2,04	2	99,36	17,52	3	93,40	2,35	3	97,10	1,61	2	80,47	0,71	3
-9	100,14	2,59	2	98,56	6,55	3	90,87	4,14	3	108,51	0,77	2	86,73	0,92	3
-8	99,25	12,19	2	91,53	1,94	3	72,47	0,49	3	90,72	2,68	3	68,92	5,96	3
-7	88,42	6,53	3	84,50	13,19	3	64,04	1,46	3	84,28	4,91	3	66,09	2,77	3
-6	10,49	1,02	3	69,60	17,85	3	14,04	1,95	3	1,03	0,15	3	0,98	2,20	3
-5	9,26	6,14	3	19,33	4,63	2	1,40	0,81	3	-9,15	1,86	3	8,85	3,05	3
-4	-0,14	1,65	3	21,57	1,20	3	0,42	4,95	3	-14,05	0,82	3	-9,09	3,19	3

**Table S5**	**12**			**3**			**1**			**5**			**2**		
**Conc. [M]**	**Mean**	**SEM**	**n**	**Mean**	**SEM**	**n**	**Mean**	**SEM**	**n**	**Mean**	**SEM**	**n**	**Mean**	**SEM**	**n**
-11	100,08	5,40	3	100,10	2,13	2	100,14	2,35	3	100,00	7,90	2	100,16	5,71	2
-10	99,64	3,58	3	110,88	5,21	2	111,24	15,89	3	97,90	3,33	2	98,42	3,10	2
-9	116,30	1,70	2	108,20	4,50	3	109,27	8,27	3	97,28	7,13	3	99,35	5,87	2
-8	103,16	0,84	3	103,85	3,27	3	85,67	4,87	3	71,36	0,57	3	85,98	1,69	3
-7	85,64	0,70	3	86,03	3,74	3	78,37	1,78	3	63,33	3,49	3	82,61	1,51	3
-6	15,82	8,71	3	13,16	0,35	3	23,46	3,65	3	2,35	0,78	3	9,67	1,19	3
-5	13,75	6,25	3	4,96	3,92	3	6,04	8,03	3	4,94	0,71	3	4,89	1,07	3
-4	5,84	1,97	3	-5,47	0,35	3	-5,62	0,16	3	-9,88	6,70	3		3,21	2

Table S6	4			10			13			14		
**Conc [M]**	**Mean**	**SEM**	**n**	**Mean**	**SEM**	**n**	**Mean**	**SEM**	**n**	**Mean**	**SEM**	**n**
-11	100,08	2,01	3	100,12	2,01	3	100,14	18,41	3	100,14	2,36	3
-10	91,59	11,54	2	98,62	3,79	2	100,28	5,32	3	95,42	2,04	2
-9	86,62	0,08	3	94,59	2,85	3	100,52	4,39	3	94,44	3,59	2
-8	98,44	4,21	2	81,01	1,94	3	97,99	8,03	3	75,21	3,25	3
-7	75,24	1,94	3	59,86	0,69	3	79,63	6,96	3	63,94	1,46	3
-6	10,46	6,37	2	2,04	4,51	3	15,03	1,05	3	22,44	10,08	3
-5	4,65	2,98	3	6,01	2,78	3	8,99	4,70	3	1,13	0,81	3
-4	0,24	1,39	3	-23,44	1,32	3	0,14	0,57	3	0,14	4,96	3

Conc. [M] – compound concentration in mol/L; Mean – mean of non-specific binding subtracted percent of [^{3}H]ketanserin displacement by the analyzed compounds from 2-3 experiments; SEM – standard error of the mean; n – number of separate experiments

Supplementary data

Tables S1-S3: Percent of specific inhibition of 8-OH-DPAT-induced G-protein stimulation at the 5-HT_{1A} receptor used to calculate IC50 values of the compounds

**Table S1**	**4**			**3**			**2**			**1**			**5**		
**Conc. [M]**	**Mean**	**SEM**	**n**	**Mean**	**SEM**	**n**	**Mean**	**SEM**	**n**	**Mean**	**SEM**	**n**	**Mean**	**SEM**	**n**
-11	116,39	1,71	3	121,35	1,89	3	122,29	2,83	2	116,39	1,71	3	127,71	1,74	3
-10	117,37	1,85	2	120,95	0,55	3	125,12	2,34	3	117,37	3,66	3	125,93	3,99	3
-9	117,80	2,67	2	129,68	0,46	3	123,68	0,18	3	116,20	2,45	3	125,05	2,89	3
-8	117,34	0,21	3	123,73	1,45	3	120,90	0,13	3	116,80	6,85	2	127,92	0,32	3
-7	116,46	2,21	3	122,41	0,44	3	124,22	0,83	3	112,24	3,10	3	132,07	2,67	2
-6	120,83	0,70	3	126,41	0,15	3	118,07	3,65	3	113,95	1,15	3	126,53	0,11	3
-5	106,98	0,87	3	126,32	0,21	3	115,05	1,28	3	118,17	1,37	3	118,51	2,49	3
-4	103,20	2,27	3	107,63	0,66	3	110,41	1,03	3	111,66	0,28	3	98,65	1,69	3

	**6**			**7**			**8**			**9**			**10**		
**Conc [M]**	**Mean**	**SEM**	**n**	**Mean**	**SEM**	**n**	**Mean**	**SEM**	**n**	**Mean**	**SEM**	**n**	**Mean**	**SEM**	**n**
-11	127,71	1,74	3	127,71	1,74	3	127,71	1,74	3	127,71	1,74	3	127,71	1,74	3
-10	129,27	1,69	3	123,98	0,78	3	127,64	4,82	3	125,53	0,90	2	131,82	1,02	3
-9	129,87	3,43	3	121,34	0,48	3	127,97	2,33	3	128,99	1,10	3	131,54	9,10	3
-8	129,82	2,22	3	129,08	2,54	3	125,60	2,41	3	132,19	0,91	3	134,79	0,74	2
-7	122,36	1,98	2	130,29	2,33	3	127,83	2,36	3	129,45	4,79	3	126,76	0,56	3
-6	120,96	1,66	3	117,67	2,54	3	124,44	0,88	3	118,00	0,48	3	121,31	1,97	2
-5	114,52	5,38	3	107,79	0,54	3	122,17	0,64	3	107,10	8,65	3	123,42	0,72	3
-4	95,08	4,18	3	82,93	2,04	3	94,43	3,53	3	110,06	3,29	3	114,75	3,16	3

	11			12			13			14		
**Conc [M]**	**Mean**	**SEM**	**n**	**Mean**	**SEM**	**n**	**Mean**	**SEM**	**n**	**Mean**	**SEM**	**n**
-11	128,76	1,20	3	128,76	1,20	3	128,76	1,20	3	128,76	1,20	3
-10	122,86	1,48	2	127,13	1,34	3	121,23	3,13	3	126,22	1,18	2
-9	125,96	0,35	3	126,83	0,16	3	127,22	0,54	3	125,01	1,55	2
-8	127,26	2,76	3	125,27	3,32	3	126,20	1,02	3	128,01	0,51	3
-7	102,29	3,59	2	127,12	0,59	3	122,86	1,54	2	133,80	1,29	3
-6	106,74	2,06	2	120,96	4,58	3	123,51	2,84	3	131,86	0,70	3
-5	101,29	1,85	3	113,95	3,16	3	118,36	1,69	3	122,63	6,24	3
-4	88,81	1,73	2	90,02	1,02	3	96,51	0,43	3	116,27	1,61	3

Conc. [M] – compound concentration in mol/L; Mean – mean of percent inhibition of 8–OH-DPAT-induced G-protein stimulation by the analyzed compounds from 2-3 experiments; SEM – standard error of the mean; n – number of separate experiments

Figure S2: Dose-response curves (A-D) of the analyzed compounds for the 5-HT_{2A} receptor.

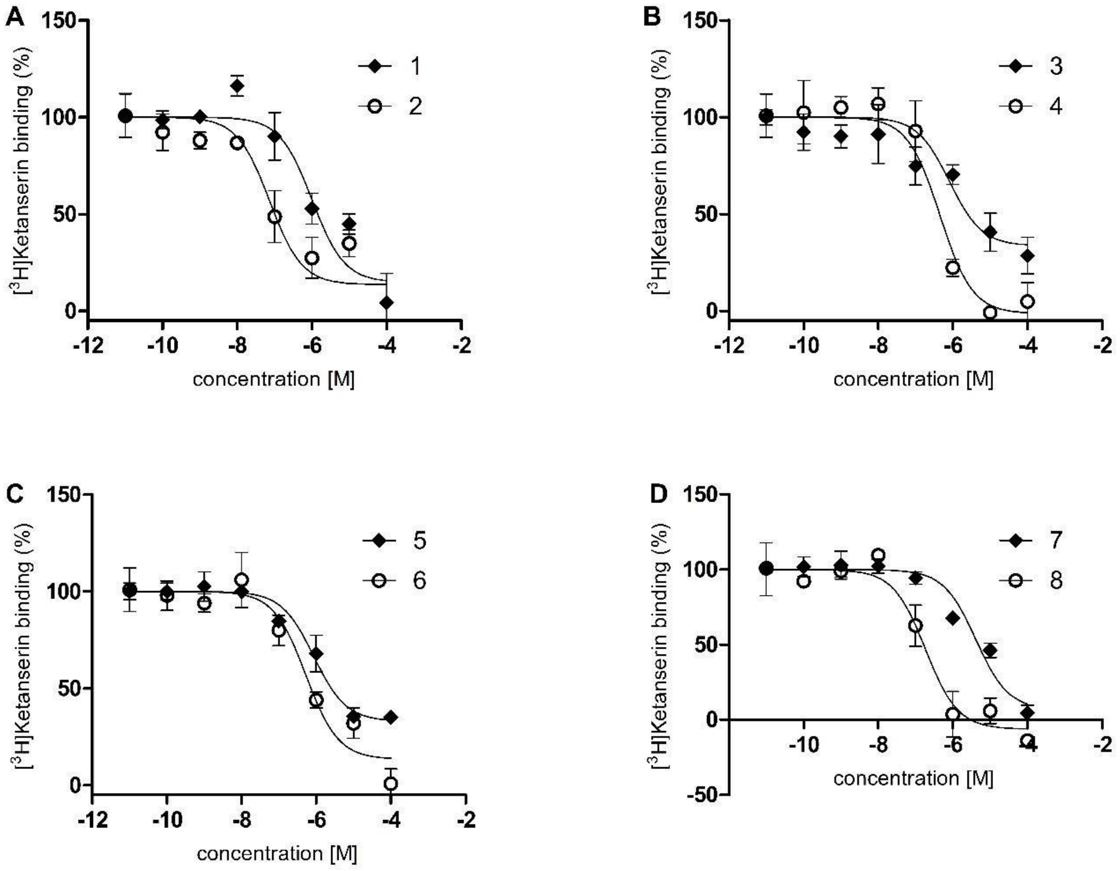

Figure S3: Dose-response curves (E-G) of the analyzed compounds for the 5-HT_{2A} receptor.

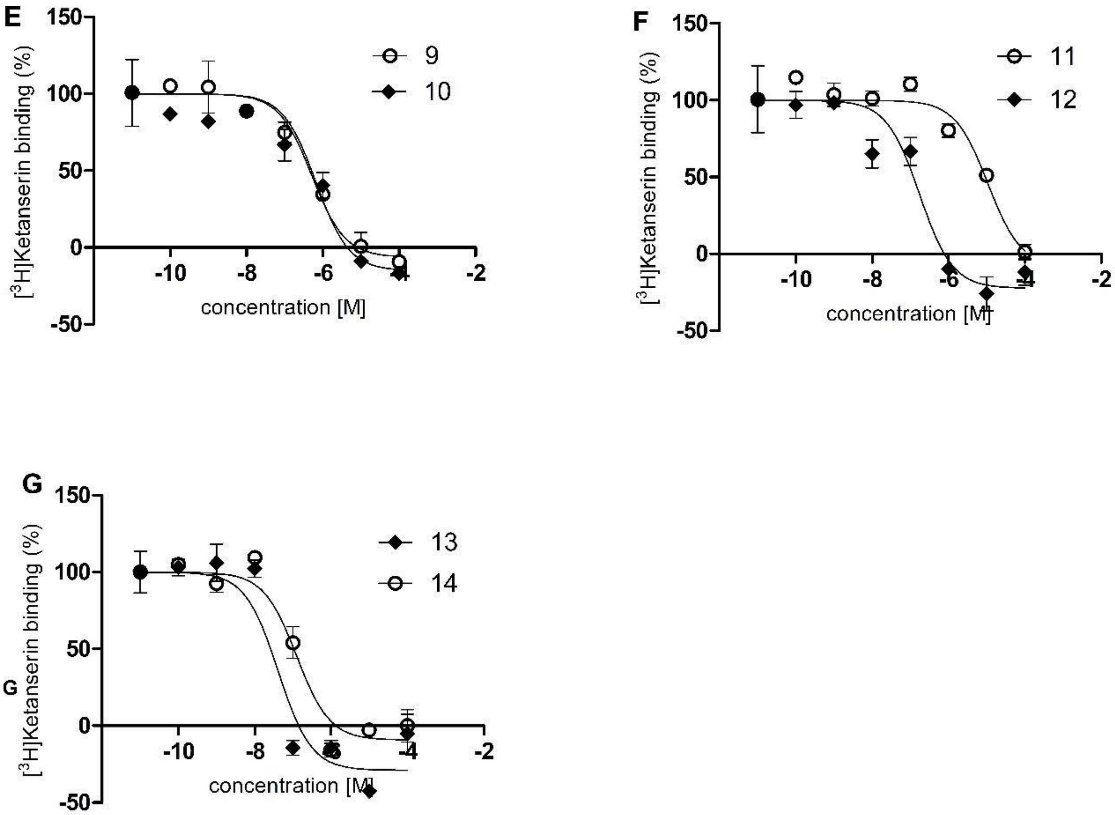

Figure S4: Dose-response curves (A-D) of the analyzed compounds for the 5-HT_{1A} receptor.

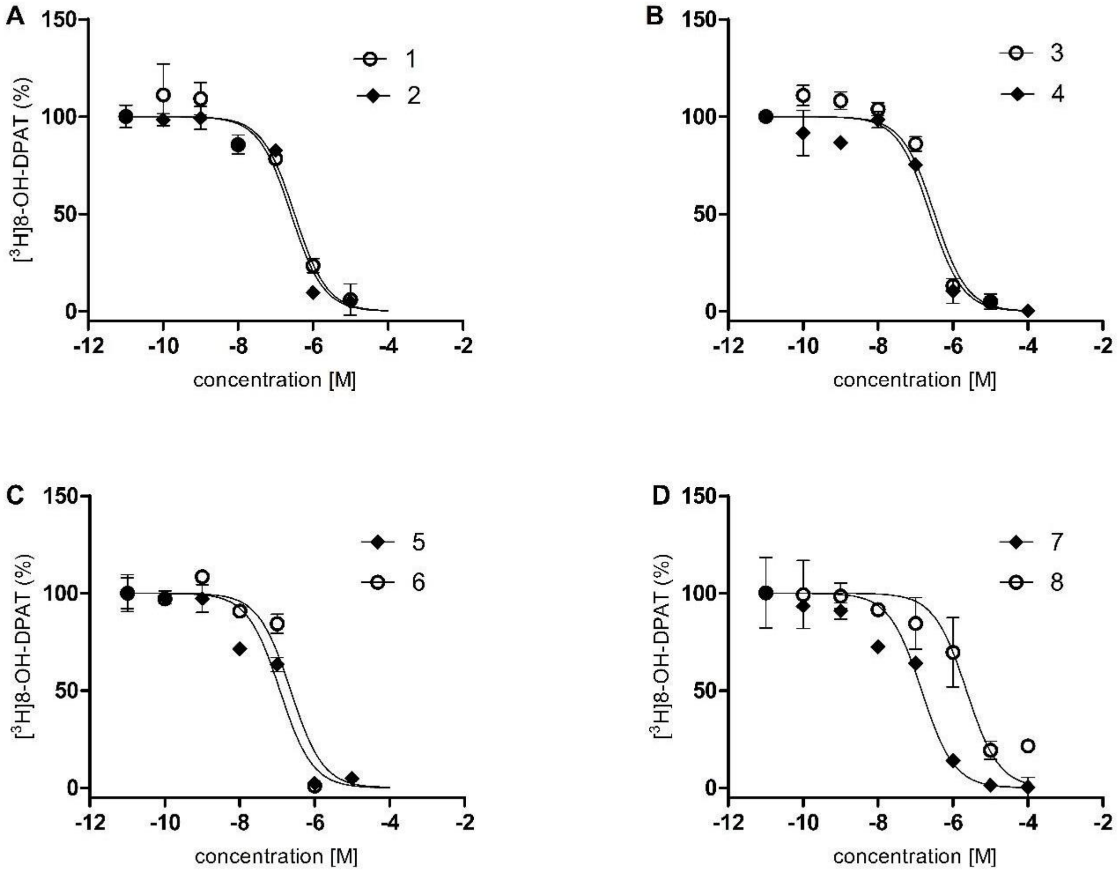

Figure S5: Dose-response curves (E-G) of the analyzed compounds for the 5-HT_{1A} receptor.

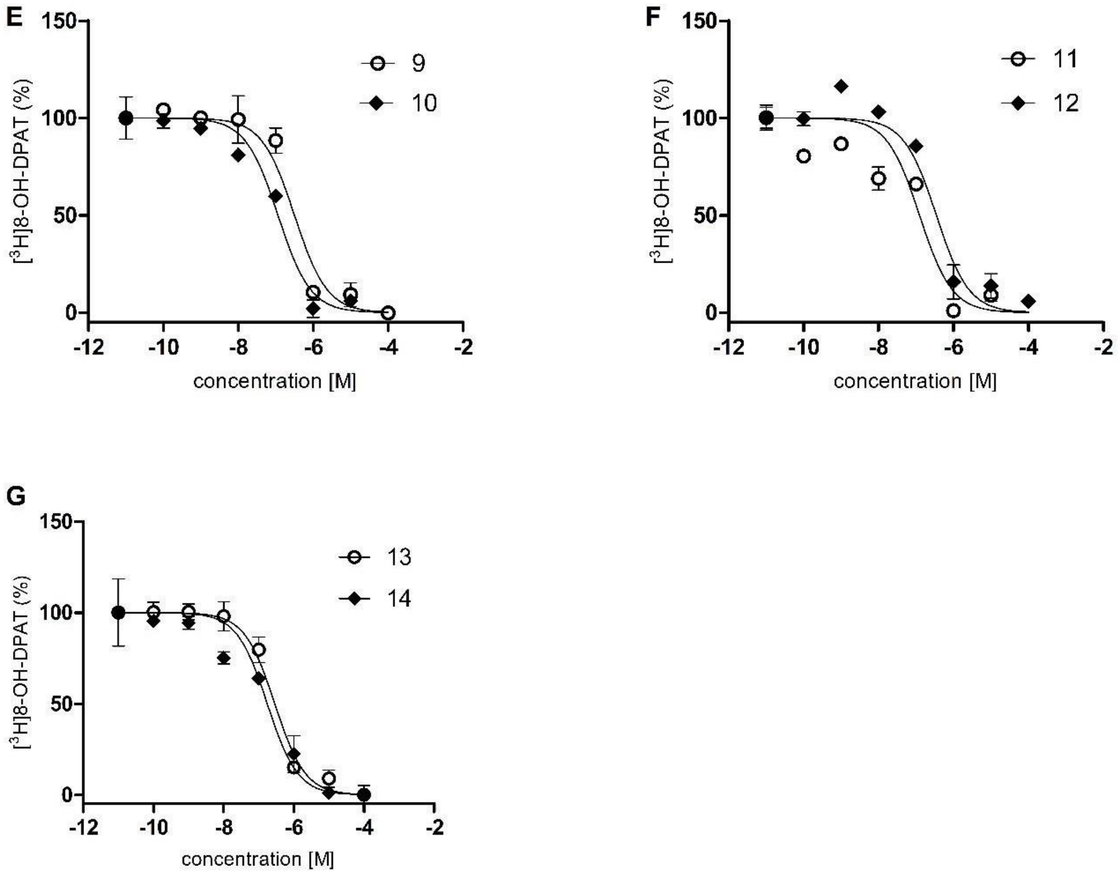

Figure S6: Dose-response curves (A-D) of 5-HT$_{1A}$ antagonism for the analyzed compounds

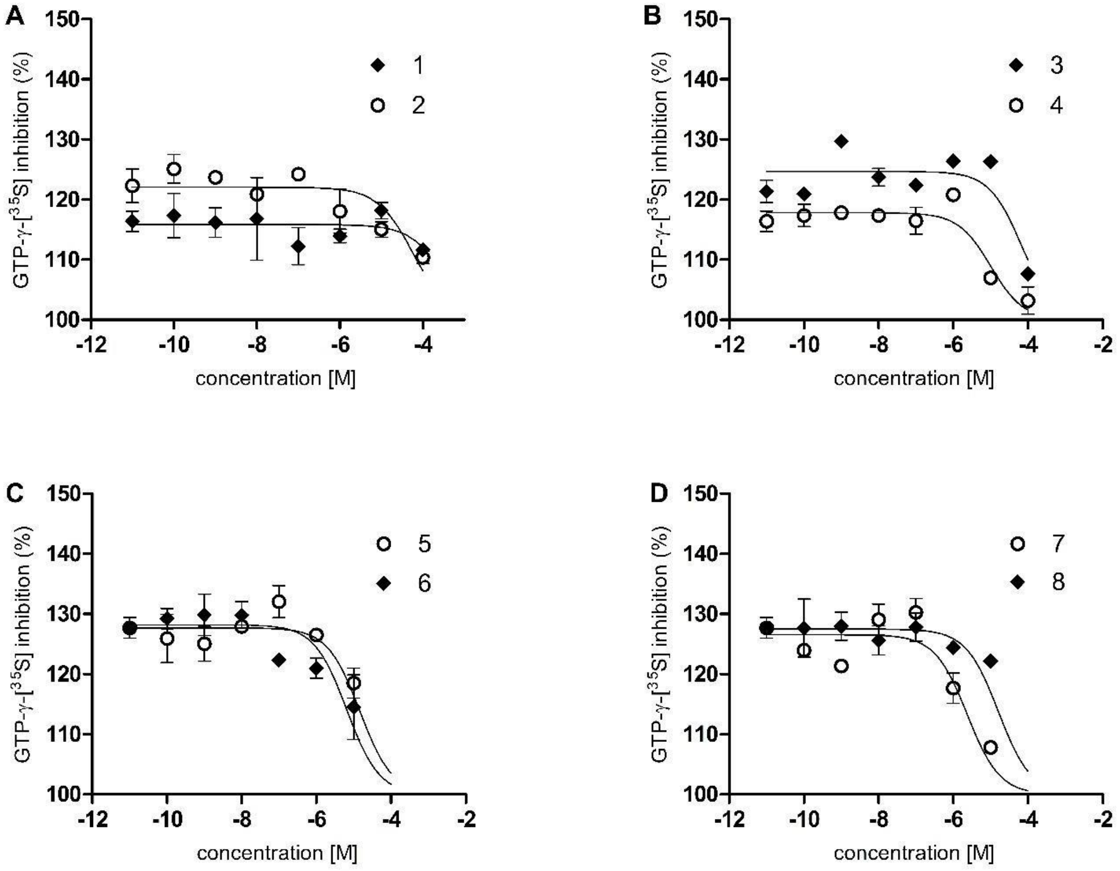

Figure S7: Dose-response curves (E-G) of 5-HT_{1A} antagonism for the analyzed compounds

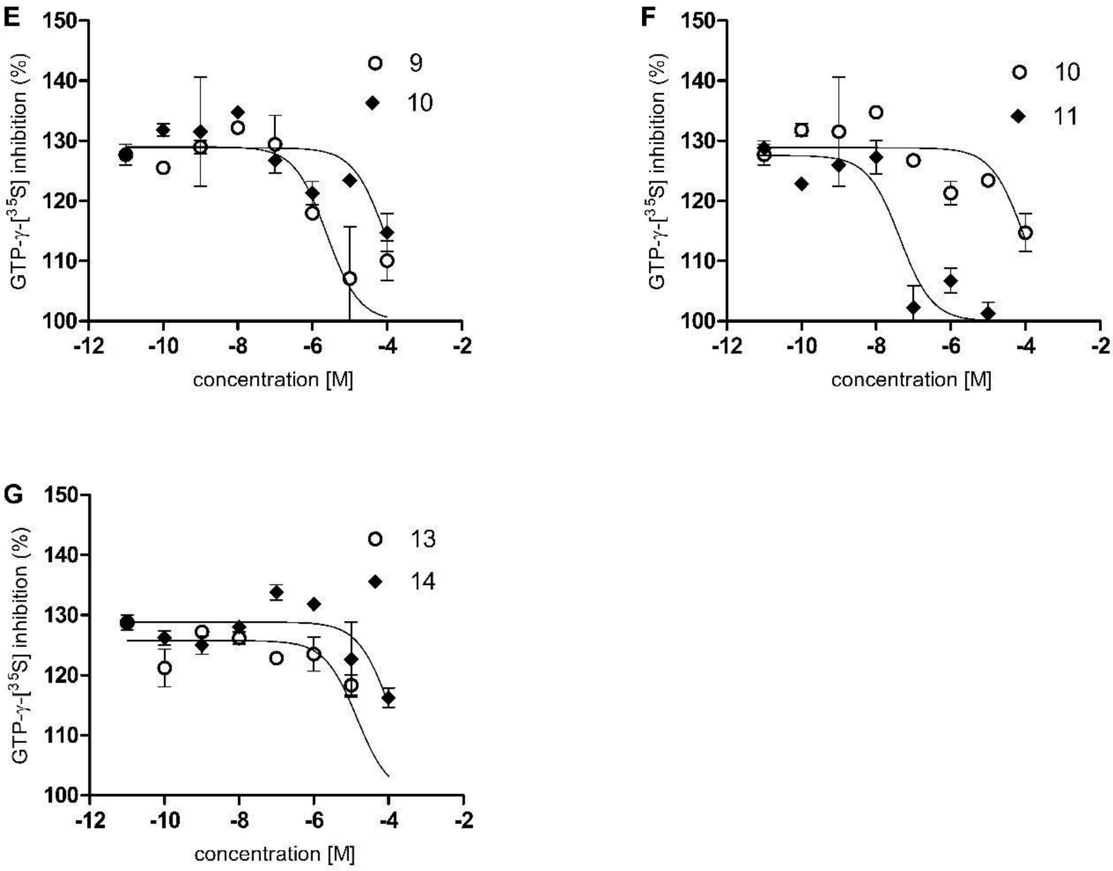

Supplement: Supplementary file 1 [file ijms-26-01946-s001.zip › ijms-3468931-supplementary.pdf]
